# User and Usability Testing of a Web-Based Genetics Education Tool for Parkinson Disease: Mixed Methods Study

**DOI:** 10.2196/45370

**Published:** 2023-08-30

**Authors:** Noah Han, Rachel A Paul, Tanya Bardakjian, Daniel Kargilis, Angela R Bradbury, Alice Chen-Plotkin, Thomas F Tropea

**Affiliations:** 1 Department of Neurology Perelman School of Medicine University of Pennsylvania Philadelphia, PA United States; 2 Department of Neurology Pennsylvania Hospital Philadelphia, PA United States; 3 Sarepta Therapeutics Cambridge, MA United States; 4 Johns Hopkins University School of Medicine Baltimore, MD United States; 5 Department of Medicine Perelman School of Medicine University of Pennsylvania Philadelphia, PA United States

**Keywords:** Parkinson disease, genetic testing, teleneurology, patient education, neurology, genetic, usability, user testing, web-based, internet-based, web-based resource, mobile phone

## Abstract

**Background:**

Genetic testing is essential to identify research participants for clinical trials enrolling people with Parkinson disease (PD) carrying a variant in the glucocerebrosidase (*GBA*) or leucine-rich repeat kinase 2 (*LRRK2*) genes. The limited availability of professionals trained in neurogenetics or genetic counseling is a major barrier to increased testing. Telehealth solutions to increase access to genetics education can help address issues around counselor availability and offer options to patients and family members.

**Objective:**

As an alternative to pretest genetic counseling, we developed a web-based genetics education tool focused on *GBA* and *LRRK2* testing for PD called the Interactive Multimedia Approach to Genetic Counseling to Inform and Educate in Parkinson’s Disease (IMAGINE-PD) and conducted user testing and usability testing. The objective was to conduct user and usability testing to obtain stakeholder feedback to improve IMAGINE-PD.

**Methods:**

Genetic counselors and PD and neurogenetics subject matter experts developed content for IMAGINE-PD specifically focused on *GBA* and *LRRK2* genetic testing. Structured interviews were conducted with 11 movement disorder specialists and 13 patients with PD to evaluate the content of IMAGINE-PD in user testing and with 12 patients with PD to evaluate the usability of a high-fidelity prototype according to the US Department of Health and Human Services Research-Based Web Design & Usability Guidelines. Qualitative data analysis informed changes to create a final version of IMAGINE-PD.

**Results:**

Qualitative data were reviewed by 3 evaluators. Themes were identified from feedback data of movement disorder specialists and patients with PD in user testing in 3 areas: content such as the topics covered, function such as website navigation, and appearance such as pictures and colors. Similarly, qualitative analysis of usability testing feedback identified additional themes in these 3 areas. Key points of feedback were determined by consensus among reviewers considering the importance of the comment and the frequency of similar comments. Refinements were made to IMAGINE-PD based on consensus recommendations by evaluators within each theme at both user testing and usability testing phases to create a final version of IMAGINE-PD.

**Conclusions:**

User testing for content review and usability testing have informed refinements to IMAGINE-PD to develop this focused, genetics education tool for *GBA* and *LRRK2* testing. Comparison of this stakeholder-informed intervention to standard telegenetic counseling approaches is ongoing.

## Introduction

Parkinson disease (PD) is the second commonest neurodegenerative disease and the fastest-growing neurological disease worldwide [1,2]. Variants in leucine-rich repeat kinase 2 (*LRRK2*), glucocerebrosidase (*GBA*), *Parkin*, Parkinsonism-associated deglycase (*DJ-1*), VPS35 retromer complex component (*VPS35*), PTEN-induced kinase 1 (*PINK1*), and α-synuclein (*SNCA*) are identified in 10%-12% of PD cases [3-9]. Despite a genetic mutation frequency similar to some cancer syndromes where germline genetic testing is common [10,11], genetic testing is not standard in the evaluation and management of PD and is rarely conducted as part of clinical care [12]. However, knowing one’s genetic status is already of key importance for research, as therapies targeting carriers of *GBA* and *LRRK2* variants are in clinical trials [13,14]. Additionally, patients with PD have expressed interest in learning their genetic information [15,16]. Research programs such as the multisite PDGENEration study sponsored by the Parkinson Foundation (ClinicalTrials.gov NCT04057794) and the University of Pennsylvania (UPenn) Molecular Integration in Neurological Diagnosis (MIND) Initiative [17] have been developed to increase genetic testing and counseling.

Recently proposed recommendations would expand clinical or research genetic testing to nearly all patients with PD [18]. However, the standard service delivery model for genetic testing includes pre- and posttest genetic counseling, which is limited by the availability of specialized genetic counselors or physicians with sufficient genetics training [19]. Indeed, there are only 125 genetic counselors specialized in neurogenetics offering in-person visits, and 82 genetic counselors offering telehealth visits listed on the National Society of Genetic Counselors’ public directory for genetic counselors. We developed a web-based education tool focused on *GBA* and *LRRK2* genetic testing called the Interactive, Multimedia Approach to Genetic Counseling to Inform and Educate in Parkinson’s Disease (IMAGINE-PD) to address this gap. IMAGINE-PD could be made available in research studies to increase genetics education prior to *GBA* and *LRRK2* testing to identify research-eligible patients with PD.

The goal of this work is to create a genetics education tool for *GBA* and *LRRK2* testing incorporating key stakeholder input. Structured interviews were conducted with movement disorder specialists (MDSs) and patients with PD to evaluate the content of IMAGINE-PD in user testing and with patients with PD to evaluate website usability according to the US Department of Health and Human Services (DHHS) Research-Based Web Design & Usability Guidelines [20]. Qualitative data analysis informed changes to create a final version of IMAGINE-PD.

## Methods

### IMAGINE-PD Website Development

**Figure 1:**
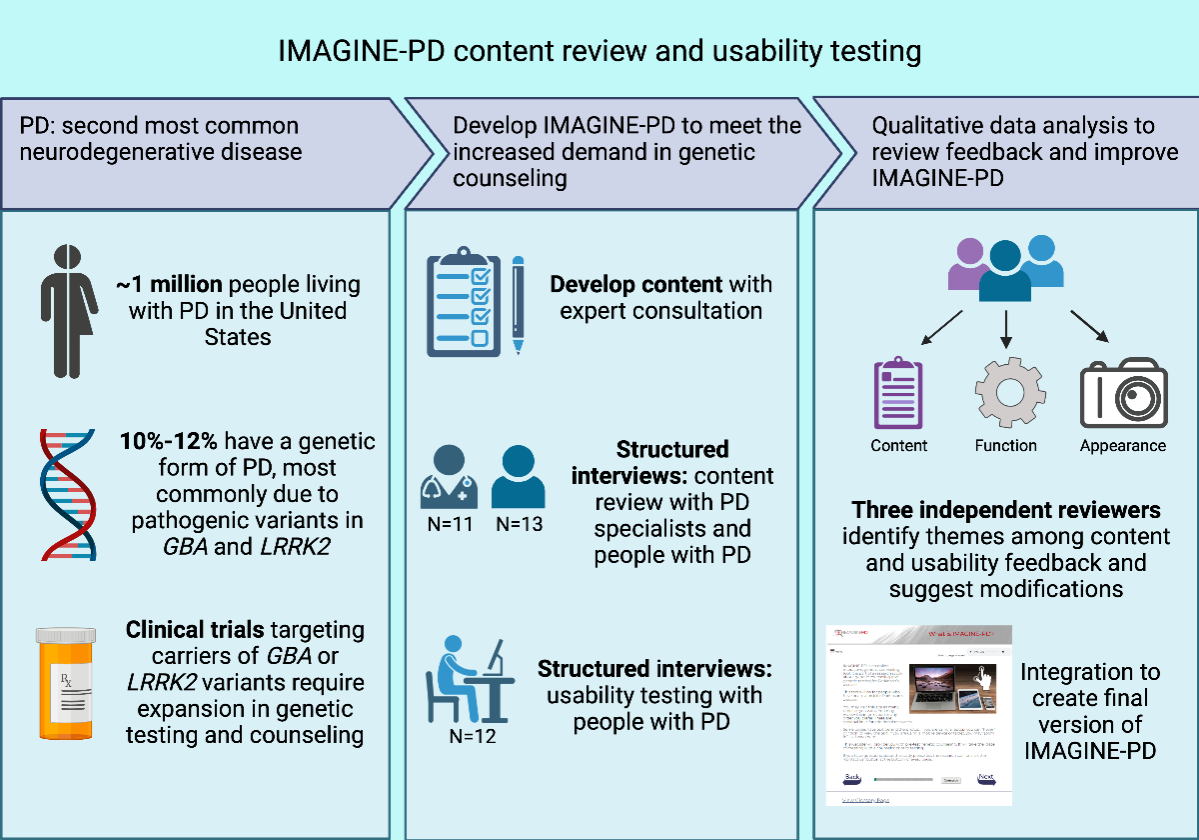
IMAGINE-PD website development flowchart. GBA: glucocerebrosidase; IMAGINE-PD: Interactive Multimedia Approach to Genetic Counseling to Inform and Educate in Parkinson’s Disease; LRRK2: leucine-rich repeat kinase 2; PD: Parkinson disease.

Website development and usability testing were conducted in accordance with the DHHS research-based web design and usability guidelines [20]. Core content of a genetic counseling tool for *GBA* and *LRRK2* variant genetic testing of PD was developed by the authors (TB, ACP, and TFT). *GBA* and *LRRK2* variant testing information was included to align with the MIND Initiative, which is a whole-clinic biobanking effort at UPenn that offers optional genetic counseling and clinical confirmation testing [17]. Content was then assigned to “primary” or essential, “secondary” or important but not essential, or “optional” categories based on their level of importance according to expert input from genetic counselors and movement disorder physicians (RAP, TB, ACP, and TFT). Five primary concepts included a review of PD, concepts of basic genetics, genetics of PD, disclosure of genetic results, and limitations and implications of genetic testing. For each primary concept, an audiovisual recording of a movement disorder physician or genetic counselor describing the concept was created. Secondary information included diagnosis, symptoms, and treatment of PD, an introduction to genetic counseling, a review of genetic testing (GT), types of genetic tests and results, risks and benefits of GT, a review of *GBA* including Gaucher disease and *LRRK2*, and a review of other genetic causes of PD that are not tested in the study (limitations). One or more slides were created to capture the secondary content important for genetic counseling including text, visuals, and an optional audio recording of the text presented. Links to outside reading were identified for optional material. All content was organized into a high-fidelity prototype website using the Wix website [21]. Once the preliminary set of material was created, we solicited feedback on content and website organization from genetic counselors and physician-scientists with expertise in neurogenetics. Changes were made to address points of feedback. A new website was created with assistance from The UPenn Center for Clinical Epidemiology and Biostatistics Clinical Research Computing Unit. This new website included the content from the prototype and was made compatible with common web browsers and operating systems to be accessible on typical connection speeds across a range of different resolutions and orientations to account for use on a computer, smartphone, or tablet computer. Number of visits and time spent on each page are captured. A computer graphics specialist with experience in medical artwork created animations [22]. With this prototype, user testing was first conducted, and changes were then incorporated into IMAGINE-PD after reviewing feedback from patients with PD. These changes included content revisions on both primary and secondary topics. Videos with movement disorders and neurogenetics specialists were professionally recorded after implementing content changes from user testing. Readability of each page was evaluated using Readable [23] to ensure a Flesch-Kincaid readability index of below 9. Usability testing was conducted subsequently, and again feedback was reviewed, and refinements were made to the content, organization, and web interface. In the final version, the basic genetics video was broken into 2 videos for a total of 6 videos. Videos range from 51 to 92 seconds in length. A link was available below the video to read a transcript of the audio recording. The final version is hosted at UPenn [24]. A flowchart of the IMAGINE-PD web development and testing process can be viewed in Figure 1.

### Participants

In the user testing phase, 13 cognitively normal patients with PD who receive care at UPenn, referred by their physicians, were enrolled. MDSs at the UPenn Parkinson’s Disease and Movement Disorders Center and the Philadelphia Veterans Affairs Parkinson’s Disease Research, Education, and Clinical Center were asked to participate (excluding authors ACP and TFT) for a total of 11 neurologists or psychiatrists. In the usability testing phase, 12 cognitively normal patients with PD who receive care at UPenn, referred by their physicians, who did not participate in the user testing phase were enrolled. No patient or physician declined participation. All patients were previously enrolled in the MIND Initiative. In both phases, sample sizes exceeded the suggestions outlined in the DHHS usability testing guidelines [20]. Identified participants were approached in person, by phone, or by email. Informed consent was obtained, and study visits were performed in person by a single evaluator (NH).

### User Testing

Structured interviews were conducted between a single evaluator with experience in conducting clinical research visits (NH) and a participant between October 31, 2019, and December 11, 2019. The evaluator neither had a prior relationship with any participant nor any assumptions or presuppositions about the outcomes of the interviews. First, participants were asked 11 questions about their general internet use, 4 questions about email and internet use for health information (adapted from Baker et al [25]), and 3 questions assessing background knowledge in genetics, PD, and genetic testing. During the interview, the evaluator asked questions of each participant, and data were entered directly into Research Electronic Data Capture (REDCap; Vanderbilt University) [26,27]. The evaluator navigated to each web page in the prototype allowing the participant to view and listen to all material on that page with unlimited time. Prompts were given to ensure all participants interacted with all aspects of each page. Feedback was solicited using 4 open-ended questions, a 1-10 rating of usefulness, and a 1-4 rating of clarity of presentation. After all web pages were reviewed, 7 additional questions were asked pertaining to the entire series of web pages and to solicit overall comments. Questionnaires are available in Multimedia Appendix 1.

### Usability Testing

The second iteration of IMAGINE-PD was created after the results of user testing were reviewed, and changes were implemented. For usability testing, structured videoconference interviews were conducted between evaluator (NH) and participant between August 18, 2020, and September 14, 2020. Again, the evaluator neither had a prior relationship with any participant nor any assumptions or presuppositions about the outcomes of the interviews. First, participants were asked 8 questions about their internet use. The participant navigated to each web page with unlimited time to view all content. Participants were asked to use the “share-screen” function so the evaluator could observe their navigation of the website. Prompts were given to ensure all participants interacted with all aspects of each page. Feedback was solicited using 8 open-ended questions asked for each page. Questionnaires are available in Multimedia Appendix 1.

### Statistical Analysis

Descriptive statistics are presented for all demographic data and scales. A qualitative content analysis plan was developed in advance of data review in consultation with Judy Shea, PhD. For both user testing review and usability testing phases, all data were collected into a spreadsheet excluding identifying information. Three evaluators (NH, RAP, and TFT) were given the following rules: (1) review the data from each web page for PD providers and patients with PD separately and identify 1 or more key themes per page, if a theme is apparent, and (2) if more than 1 theme is apparent, order themes for each page in order of importance or frequency. Data were independently reviewed by each evaluator to improve the trustworthiness of the analysis. Subsequently, the 3 evaluators met to establish a consensus on the key themes. Suggestions for changes to the website were made after all themes were reviewed and discussed among the evaluators.

### Ethics Approval

Institutional review board (IRB) approval (UPenn IRB 834311) was obtained before initiating the study, and informed consent was obtained from all participants prior to any study activities. This research adheres to the principles set out in the Declaration of Helsinki.

## Results

### Website Development

The final version includes 27 web pages, including 3 video-only pages and 3 video pages with animations. Audiovisual pages also include accessible text for participants with hearing impairment. Each nonvideo page included audio recording of the text for auditory learners. The website is accessible by and formatted for computer browsers, mobile devices, or tablets. The length of time to view all videos is 7 minutes 42 seconds, and the estimated time to review all website content is 25-40 minutes. The Flesch-Kincaid reading level for all pages was a median of 8.3 (IQR 7.375-8.25) indicating all pages on the website maintained approximately an eighth-grade leading level. Screenshots of each web page are available in Multimedia Appendix 2.

### User Testing

**Figure 2:**
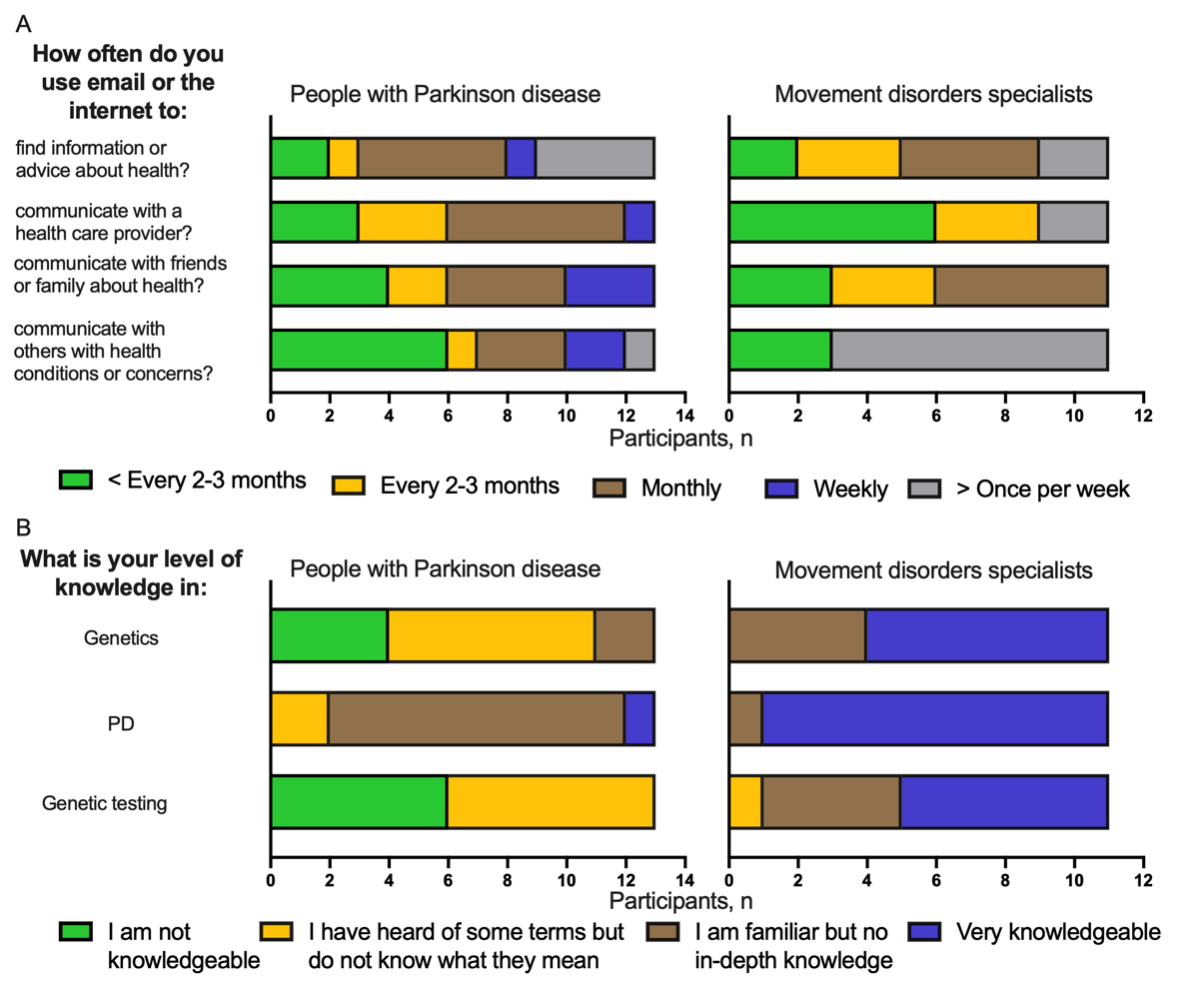
(A) Patients with PD and MDS self-reported use of the internet and email for health information. (B) PD patients and MDS self-reported knowledge in genetics, PD, and genetic testing (GT). MDS: movement disorder specialist; PD: Parkinson disease.

Participant demographics are summarized in Table 1. Email and internet use for health information is described in Figure 2A and Table S1 in Multimedia Appendix 1. PD, genetics, and genetic testing knowledge are reported in Figure 2B.

**Table 1:** Cohort description.

		Content review			Usability
		PD^a^ (N=13)	Physicians (N=11)	PD (N=12)	
Age at test (years), median (IQR)		64 (63-69)	N/A^b^	63 (59-72)	
**Sex, n (%)**					
	Female	3 (23)	4 (36)	5 (42)	
	Male	10 (77)	7 (64)	7 (58)	
Disease duration (years), median (IQR)		11 (7-13)	N/A	8 (5-13)	
Education (years), median (IQR)		17 (15-18)	N/A	16 (16-17)^c^	

^a^PD: patients with Parkinson disease.

^b^N/A: not applicable.

^c^Two values excluded due to missing data.

For each web page, participants were asked to rate the usefulness (1 being the least useful and 10 being the most useful). The average usefulness across all pages for patients with PD was 8.49 (SD 0.65) and for MDS was 8.85 (SD 0.64). The results of usefulness for each page are found in Table S2 in Multimedia Appendix 1.

In user testing, content feedback pertained to the volume and level of detail of information (too much information), the complexity of the content (wording is too technical), and the focus of the content (make it more related to PD). Specifically, the amount of information was reported to be too much by patients on 13 occasions by 3 different physicians and 4 different patients pertaining to 11 different pages. Representative comments included “Gaucher's disease may be a little confusing. May not need to go into so much detail” and “alpha synuclein was confusing. Maybe too much content here.” On 6 different occasions, 6 different patients reported the topic, and the language used to be too technical including words like “bradykinesia” and “brain imaging.” Content was mentioned on 3 occasions by 2 different physicians and 1 patient pertaining to 3 different pages. Representative comments included “focus more on PD related genetic testing,” and “mention these are two genes we are looking at and this is why it is related to PD.” To address these points, we made changes to focus and simplify the content and to use plain language at a Flesch-Kincaid reading level 9 or below.

Three domains of feedback were identified on user testing analysis: content, function, and appearance. These domains were consistent between patient and physician participants, and suggestions for change were made based on the combined review of both sets of responses. Changes were made on 20 pages in response to content feedback, 4 pages in response to function feedback, and 4 pages in response to appearance feedback. A summary of the user testing qualitative analysis and recommendations for change are shown in Tables 2-4.

**Table 2:** Summary of content changes made in response to user testing.

Page	Content feedback	Changes made
1. Introduction	Want more information on this page	Short summary was added to introduction
3. What is IMAGINE-PD^a^	Not sure how the website is relevant to PD^b^	Clarified study relevance to PD
4. Summary	Wording is too technical	Simplified vocabulary
5. What is PD	Wording is too technical	Simplified vocabulary
6. How is PD diagnosed	Want more information on nonmotor symptoms	Information on nonmotor symptoms included
7. What causes PD	Too much information on page	Information was cut down and simplified
8. How is PD treated	Want less information on drugs, more information on other treatments	Drug section shortened and added other PD treatments
9. What is genetics	Too much information on page	Information was shortened and simplified
10. Basics of genetics	Too much information on page and too technical	Information was shortened and simplified
11. What is GT^c^	Wording is too technical and not sure how information is related to PD	Simplified vocabulary, clarified relevance
12.5. Reasons for GT	Page should be included as a separate page	Separate page was made for information
13. Benefits and risks	Make it more related to PD and more information on benefits	Clarified PD relevance
14. GT process	Too much detail	Simplified a shortened page
15. How genetics affects PD	Too much information	Shortened page
16. *GBA*^d^ and PD	Information too technical and want to know why this gene is being studied	Simplified vocabulary and clarified the relevance of *GBA*
17. *LRRK2*^e^ and PD	Information too technical and want to know why this gene is being studied	Simplified vocabulary and clarified the relevance of *LRRK2*
18. Other rare genes for PD	Language seems too technical, why only *GBA* and *LRRK2* being tested	Simplified language, provides more resources for additional information on other genes
19. How do I get results	Language seems too technical, want more information	Clarified next steps and reviewed previously introduced topics to clarify information
20. VUS^f^ and unexpected results	Wording too technical	Simplified language
21. Implications and limitations	Confused about previously introduced topics	Added information to clarify

^a^IMAGINE-PD: Interactive Multimedia Approach to Genetic Counseling to Inform and Educate in Parkinson’s Disease.

^b^PD: Parkinson disease.

^c^GT: genetic testing.

^d^GBA: glucocerebrosidase.

^e^LRRK2: leucine-rich repeat kinase 2.

^f^VUS: variant of uncertain significance.

**Table 3:** Summary of function changes made in response to user testing.

Page	Function feedback	Changes made
2. Instructions	Did not know that there was more information at the bottom and needed to scroll	Shortened page to minimize scrolling
4. Summary	Picture was clickable but did not lead to any new page	Fixed error so picture was no longer clickable
5. What is PD^a^	Already knew information, wanted a skip function	Shortened page, so not as much time would be spent on it
6. How is PD diagnosed	In page slides were confusing	Made in page slides more obvious

^a^PD: Parkinson disease.

**Table 4:** Summary of appearance changes made in response to user testing.

Page	Appearance feedback	Changes made
1. Introduction	Make title clearer and more obvious	Title was enlarged and bolded
3. What is IMAGINE-PD^a^	Use bullet points to make page easier to read	Used bullet points
4. Summary	Picture was not relevant	Used a different picture
12. When to consider GT^b^	Did not like picture	Used a different picture

^a^IMAGINE-PD: Interactive Multimedia Approach to Genetic Counseling to Inform and Educate in Parkinson’s Disease.

^b^GT: genetic testing.

At the conclusion, participants were asked to provide summary feedback. A summary of responses is found in Table S3 in Multimedia Appendix 1. Comments regarding the order of the presented information, the web-based tools such as the buttons, links, and menus, and overall comments are provided in Table S4 in Multimedia Appendix 1.

### Usability Testing

Participant demographics are summarized in Table 1. All participants reported using the internet or email within the past 12 months. Overall, 1 (8.3%) participant reported dial-up network use, 9 (75%) participants reported broadband network use, 7 (58%) participants reported using smartphones, and 8 (67%) participants reported accessing the internet via a Wi-Fi network. All participants reported using the internet to communicate with a health care provider, and 9 (75%) participants reported using the internet to search for health or medical information.

In usability testing, technical language was reported on 10 occasions, while the volume of information was reported on 6 occasions. Representative comments included “technical and a little difficult to understand” and “it was a little too much info in one video.” Based on usability testing feedback, further refinements were made as outlined in Tables 5-7. In addition to simplifying language, we included a glossary page, accessible via a link on every page, that defined key terms organized by concept. We also included a “Contact Us” page allowing participants to request additional information from a genetic counselor. Key points of feedback about website function included requests for a progress bar and mobile compatibility, which were both addressed for the final version. Improved audio quality was also suggested. Additionally, feedback on the difficulty of navigation through the website was mentioned by patients with PD and was addressed by creating clear instructions for navigation at the beginning of the website and large labels for website progression. Feedback about appearance included font color and size as well as picture choice. Final pictures were selected to ensure the representation of individuals of diverse race, ethnicity, age, and sex.

**Table 5:** Summary of content changes made in response to usability testing.

Page	Content feedback	Changes made
1. Introduction	Want more information on this page	Short summary was added to introduction
2. What is IMAGINE-PD^a^	Too much information	Cut down on information
6. Genetics introduction	Too much information all at once	Added animations and broke page to 2 pages
7. VUS^b^	Wording is too technical	Removed to focus on the relevant testing and simplify material
12. Implications and limitations to GT^c^	Accent was hard to follow at first	Added page with script of video
14. *GBA*^d^ page 1	Confused about *GBA* in Ashkenazi Jews, information too technical	Information was cut down and simplified
15. *GBA* page 2	Information too technical	Simplified information
16. *GBA* page 3	Want more relevance to PD^e^	Clarified the relevance of *GBA* to PD
17. *LRRK2*^f^ page 1	Information too technical	Simplified information
18. *LRRK2* page 2	Information too technical	Simplified information

^a^IMAGINE-PD: Interactive Multimedia Approach to Genetic Counseling to Inform and Educate in Parkinson’s Disease.

^b^VUS: variant of uncertain significance.

^c^GT: genetic testing.

^d^GBA: glucocerebrosidase.

^e^PD: Parkinson disease.

^f^LRRK2: leucine-rich repeat kinase 2.

**Table 6:** Summary of function changes made in response to usability testing.

Page	Function feedback	Changes made
1. Title page	Did not know what to do	Clarified next steps, enlarged arrow to move to next page
2. What is IMAGINE-PD^a^	Hovering function was not compatible with mobile device	Made function easier to use with mobile device
3. What is PD^b^	Want a progress bar to see the length of the website	Added a progress bar to the website
4. PD diagnosis or treatment	In page slides were confusing	Removed in page slides
8. What is GT^c^	Page incompatible with mobile	Improved mobile compatibility
9. When to consider GT	Hard to navigate between external resources page	Provide instructions on navigation

^a^IMAGINE-PD: Interactive Multimedia Approach to Genetic Counseling to Inform and Educate in Parkinson’s Disease.

^b^PD: Parkinson disease.

^c^GT: genetic testing.

**Table 7:** Summary of appearance changes made in response to usability testing.

Page	Appearance feedback	Changes made
1. Title page	Make title acronym clearer and change font color	Title acronym was made more obvious and changed font color
4. PD^a^ diagnosis or treatment	Some of the text was too small	Increased text font
7. VUS^b^	Graphic was confusing	Clarified graphic
9. When to consider GT^c^	Want more diverse pictures	Included more diverse pictures
10. Benefits and risks	Colors were too vibrant and hurt eyes	Toned down color scheme
15. *GBA*^d^ page 2	Did not like picture	Changed the picture
20. Conclusion	Did not like red font	Changed font color

^a^PD: Parkinson disease.

^b^VUS: variant of uncertain significance.

^c^GT: genetic testing.

^d^GBA: glucocerebrosidase.

The same 3 themes were again identified on usability testing qualitative analysis: content, function, and appearance. Changes were made on 10 pages in response to content feedback, 6 pages in response to function feedback, and 7 pages in response to appearance feedback. A summary of the usability qualitative analysis and recommendations for change are shown in Tables 5-7.

## Discussion

### Principal Results

In this study, we evaluated a web-based genetics education tool for PD using evidence-based research methods to refine the content and conduct usability testing. First, content was developed based on expert opinion, and a high-fidelity prototype was created. Next, user testing was conducted through structured interviews with MDS and patients with PD to evaluate website content. Subsequently, usability testing was conducted via structured interviews with patients with PD. Using qualitative data analysis in both phases, we identified 3 domains of feedback (content, function, and appearance) and addressed feedback by incorporating changes to IMAGINE-PD to create a final version.

### Comparison With Prior Work

Some studies in PD and Alzheimer disease have used alternative media forms as pretest education tools. For instance, the PDGENEration study, which offers genetic testing and counseling for PD, provides a pretest education tool that is a prerecorded video covering essential topics in PD genetics and genetic testing that was created by experts in neurogenetics and genetic counseling for PD (ClinicalTrials.gov NCT04057794). Additionally, the Alzheimer’s Prevention Initiative, which conducts apolipoprotein E testing in cognitively unimpaired people 60 years or older, uses a self-directed learning technique providing a brochure and a video covering content typically addressed in a pretest counseling session coupled with multiple-choice questions to reinforce learning [28]. To our knowledge, neither approach has undergone usability testing to incorporate the input of the end user as we demonstrate in this study.

Beyond neurogenetics, these results can also be viewed in the context of alternative genetic education tools, where more robust efforts to develop alternate education and disclosure methods are underway. A novel, web-based genetics education for a polygenic risk score of alcohol use disorders was evaluated in a randomized clinical trial of 325 college students. The tool was shown to improve user knowledge compared to general alcohol-use education alone [29]. In another study, a tool named Decision-Aid and E-Counselling for Inherited Disorder Evaluation [30] was developed to educate about genome-wide sequencing. Decision-Aid and E-Counselling for Inherited Disorder Evaluation was compared to pretest genetic counseling with a counselor; the genetics education methods were equivalent in conveying knowledge and were highly satisfactory to participants [31]. In the ongoing Communication and Education in Tumor Profiling and the Returning Genetic Research Panel Results for Breast Cancer Susceptibility studies, a web-based educational tool for pretest education was developed. These served as a guide for user testing and usability testing of IMAGINE-PD [32,33]. Furthermore, in the Study of an eHealth Delivery Alternative for Cancer Genetic Testing for Hereditary Predisposition in Metastatic Cancer Patients, web-based alternatives to traditional provider–mediated counseling and results disclosure are being evaluated. The outcomes of these studies will be informative for understanding the use of web-based genetic education tools even beyond inherited cancer syndromes. However, differences in the target populations, genetic testing performed, and the implications of the genetic test results between IMAGINE-PD and the Communication and Education in Tumor Profiling, Returning Genetic Research Panel Results for Breast Cancer Susceptibility, and Study of an eHealth Delivery Alternative for Cancer Genetic Testing for Hereditary Predisposition in Metastatic Cancer Patients studies necessitated the rigorous user testing and usability testing that we report here.

This study has several strengths that should be noted. First, the content for IMAGINE-PD was developed by experts in genetic counseling in PD, neurogenetics experts, and movement disorder physicians. Second, user testing included referring providers for neurogenetic services for patients with PD (movement disorder physicians) and the intended end user (patients with PD). Third, this evaluation followed the DHHS guidelines for user testing and usability testing, nearly doubling the recommended sample size in each phase for this type of research.

### Limitations

Some limitations of this study should be acknowledged. First, the content of IMAGINE-PD is focused on targeted variant testing in *GBA* and *LRRK2*, limiting its scope and generalizability to other PD genetic testing. This was intentional to match the research-based *GBA* and *LRRK2* screening being performed in the MIND Initiative at UPenn [17]. IMAGINE-PD may serve as the genetics education tool for MIND participants. Additionally, the MIND Initiative conducts the same genetic test for all involved study participants. As a result, the pretest education in the IMAGINE-PD tool deviates from a typical pretest genetic counseling session that would involve obtaining a family history and making decisions about test choices (family variant testing, multigene panel testing, exome, or genome sequencing). Instead, this is a scalable approach to screen everyone in the UPenn PD clinic for variants within the 2 most common genes associated with PD and identify potentially eligible participants for clinical trials enrolling carriers of variants in *GBA* or *LRRK2*. During a separate disclosure visit for *GBA* or *LRRK2,* a personal and family history could be reviewed in detail, and additional testing could be pursued afterward if indicated. Additionally, specialized content could be developed and added to subsequent versions of IMAGINE-PD to accommodate other specific types of diagnostic genetic testing. Second, all participants (physicians and patients) had a high level of education and experience with technology and computer use and interest in using a web-based pretest education tool, which probably does not capture the breadth of patients with PD and may overestimate the user experience. Ongoing evaluation of this tool will be necessary to determine which patients will be able to successfully use a web-based educational platform and who would be better served by other methods such as in-person or live telemedicine counseling with a genetic counselor. Although patients with PD were not involved in content creation, the content was derived from principals based on genetic counseling expertise. Patient feedback on content was solicited during both user and usability testing, where participants were given free answer choices to provide input on any additional topics they would want to have included. In the future, supplementary content could be developed to address deficits in patient or family-member comprehension, low literacy or education level, identifying potential psychosocial concerns to prompt additional counseling or comfort with technology. Making this tool accessible while in clinic on a smartphone, tablet, or computer screen may help to address limitations in access to technology. Third, participants elected to be enrolled in a genetic biobanking study and therefore may not represent the community with PD more broadly.

### Conclusions

In summary, we present our findings from user testing and usability testing for the development of IMAGINE-PD, a web-based pretest genetic education tool. We describe a phased review and iterative process of refining the content, appearance, and functionality based on expert review as well as physician and patient feedback according to DHHS guidelines. The final version [24], which can be made available by request to the authors, will undergo further evaluation to compare it to standard telegenetic counseling with a genetic counselor (ClinicalTrials.gov NCT04527146) measuring satisfaction, impact, and comprehension. As a web-based learning tool accessible by internet, IMAGINE-PD has the potential to improve access to neurogenetic services for patients with PD interested in learning about their eligibility for *LRRK2*- or *GBA*-directed clinical trials.

## Appendix

Multimedia Appendix 1 Supplemental tables and supplemental questionnaires for user and usability testing of IMAGINE-PD.

Multimedia Appendix 2 Screenshots of all web pages in IMAGINE-PD. IMAGINE-PD: Interactive Multimedia Approach to Genetic Counseling to Inform and Educate in Parkinson’s Disease.

## Abbreviations

DHHS: US Department of Health and Human Services
DJ-1: Parkinsonism-associated deglycase
GBA: glucocerebrosidase
GT: genetic testing
IMAGINE-PD: Interactive Multimedia Approach to Genetic Counseling to Inform and Educate in Parkinson’s Disease
IRB: institutional review board
LRRK2: leucine-rich repeat kinase 2
MDS: movement disorder specialist
MIND: Molecular Integration in Neurological Diagnosis
PD: Parkinson disease
Pink1: PTEN-induced kinase 1
REDCap: Research Electronic Data Capture
SNCA: α-synuclein
UPenn: University of Pennsylvania
VPS35: VPS35 retromer complex component

## Data Availability

The data sets generated and analyzed during this study are available from the corresponding author upon reasonable request. All deidentified data can be made available upon request of the authors.

## References

[1] Marras C, Beck JC, Bower JH, Roberts E, Ritz B, Ross GW, Abbott RD, Savica R, Van Den Eeden SK, Willis AW, Tanner CM, Parkinson’s Foundation P4 Group (2018). Prevalence of Parkinson's disease across North America. NPJ Parkinsons Dis.

[2] GBD 2016 Parkinson's Disease Collaborators (2018). Global, regional, and national burden of Parkinson's disease, 1990-2016: a systematic analysis for the Global Burden of Disease Study 2016. Lancet Neurol.

[3] Kitada T, Asakawa S, Hattori N, Matsumine H, Yamamura Y, Minoshima S, Yokochi M, Mizuno Y, Shimizu N (1998). Mutations in the parkin gene cause autosomal recessive juvenile parkinsonism. Nature.

[4] Valente EM, Bentivoglio AR, Dixon PH, Ferraris A, Ialongo T, Frontali M, Albanese A, Wood NW (2001). Localization of a novel locus for autosomal recessive early-onset parkinsonism, PARK6, on human chromosome 1p35-p36. Am J Hum Genet.

[5] Bonifati V, Rizzu P, Squitieri F, Krieger E, Vanacore N, van Swieten JC, Brice A, van Duijn CM, Oostra B, Meco G, Heutink P (2003). DJ-1( PARK7), a novel gene for autosomal recessive, early onset parkinsonism. Neurol Sci.

[6] Polymeropoulos MH, Lavedan C, Leroy E, Ide SE, Dehejia A, Dutra A, Pike B, Root H, Rubenstein J, Boyer R, Stenroos ES, Chandrasekharappa S, Athanassiadou A, Papapetropoulos T, Johnson WG, Lazzarini AM, Duvoisin RC, Di Iorio G, Golbe LI, Nussbaum RL (1997). Mutation in the alpha-synuclein gene identified in families with Parkinson's disease. Science.

[7] Lesage S, Brice A (2009). Parkinson's disease: from monogenic forms to genetic susceptibility factors. Hum Mol Genet.

[8] Aharon-Peretz J, Rosenbaum H, Gershoni-Baruch R (2004). Mutations in the glucocerebrosidase gene and Parkinson's disease in Ashkenazi Jews. N Engl J Med.

[9] Sidransky E, Lopez G (2012). The link between the GBA gene and parkinsonism. Lancet Neurol.

[10] Norquist BM, Harrell MI, Brady MF, Walsh T, Lee MK, Gulsuner S, Bernards SS, Casadei S, Yi Q, Burger RA, Chan JK, Davidson SA, Mannel RS, DiSilvestro PA, Lankes HA, Ramirez NC, King MC, Swisher EM, Birrer MJ (2016). Inherited mutations in women with ovarian carcinoma. JAMA Oncol.

[11] Ma H, Brosens LAA, Offerhaus GJA, Giardiello FM, de Leng WWJ, Montgomery EA (2018). Pathology and genetics of hereditary colorectal cancer. Pathology.

[12] Alcalay RN, Kehoe C, Shorr E, Battista R, Hall A, Simuni T, Marder K, Wills AM, Naito A, Beck JC, Schwarzschild MA, Nance M (2020). Genetic testing for Parkinson disease: current practice, knowledge, and attitudes among US and Canadian movement disorders specialists. Genet Med.

[13] Mullin S, Smith L, Lee K, D'Souza G, Woodgate P, Elflein J, Hällqvist J, Toffoli M, Streeter A, Hosking J, Heywood WE, Khengar R, Campbell P, Hehir J, Cable S, Mills K, Zetterberg H, Limousin P, Libri V, Foltynie T, Schapira AHV (2020). Ambroxol for the treatment of patients with Parkinson disease with and without glucocerebrosidase gene mutations: a nonrandomized, noncontrolled trial. JAMA Neurol.

[14] Our pipeline. Denali Therapeutics.

[15] Falcone DC, Wood EM, Xie SX, Siderowf A, Van Deerlin VM (2011). Genetic testing and Parkinson disease: assessment of patient knowledge, attitudes, and interest. J Genet Couns.

[16] Gupte M, Alcalay RN, Mejia-Santana H, Raymond D, Saunders-Pullman R, Roos E, Orbe-Reily M, Tang MX, Mirelman A, Ozelius L, Orr-Urtreger A, Clark L, Giladi N, Bressman S, Marder K (2015). Interest in genetic testing in Ashkenazi Jewish Parkinson's disease patients and their unaffected relatives. J Genet Couns.

[17] Tropea TF, Amari N, Han N, Rick J, Suh E, Akhtar RS, Dahodwala N, Deik A, Gonzalez-Alegre P, Hurtig H, Siderowf A, Spindler M, Stern M, Thenganatt MA, Weintraub D, Willis AW, Van Deerlin V, Chen-Plotkin A (2021). Whole clinic research enrollment in Parkinson's disease: the molecular integration in neurological diagnosis (MIND) study. J Parkinsons Dis.

[18] Cook L, Schulze J, Kopil C, Hastings T, Naito A, Wojcieszek J, Payne K, Alcalay RN, Klein C, Saunders-Pullman R, Simuni T, Foroud T (2021). Genetic testing for Parkinson disease: are we ready?. Neurol Clin Pract.

[19] Rubanovich CK, Cheung C, Mandel J, Bloss CS (2018). Physician preparedness for big genomic data: a review of genomic medicine education initiatives in the United States. Hum Mol Genet.

[20] Research-based web design & usability guidelines. United States General Services Administration.

[21] Wix.

[22] SP Motion Design.

[23] Readable.

[24] IMAGINE-PD. Penn Medicine.

[25] Baker L, Wagner TH, Singer S, Bundorf MK (2003). Use of the Internet and e-mail for health care information: results from a national survey. JAMA.

[26] Harris PA, Taylor R, Thielke R, Payne J, Gonzalez N, Conde JG (2009). Research electronic data capture (REDCap)—a metadata-driven methodology and workflow process for providing translational research informatics support. J Biomed Inform.

[27] Harris PA, Taylor R, Minor BL, Elliott V, Fernandez M, O'Neal L, McLeod L, Delacqua G, Delacqua F, Kirby J, Duda SN, REDCap Consortium (2019). The REDCap consortium: building an international community of software platform partners. J Biomed Inform.

[28] Langlois CM, Bradbury A, Wood EM, Roberts JS, Kim SYH, Riviere ME, Liu F, Reiman EM, Tariot PN, Karlawish J, Langbaum JB (2019). Alzheimer's Prevention Initiative Generation Program: development of an genetic counseling and disclosure process in the context of clinical trials. Alzheimers Dement (N Y).

[29] Driver MN, Kuo SIC, Petronio L, Brockman D, Dron JS, Austin J, Dick DM (2022). Evaluating the impact of a new educational tool on understanding of polygenic risk scores for alcohol use disorder. Front Psychiatry.

[30] Birch P, Adam S, Bansback N, Coe RR, Hicklin J, Lehman A, Li KC, Friedman J M (2016). DECIDE: a decision support tool to facilitate parents' choices regarding genome-wide sequencing. J Genet Couns.

[31] Adam S, Birch PH, Coe RR, Bansback N, Jones AL, Connolly MB, Demos MK, Toyota EB, Farrer MJ, Friedman JM (2018). Assessing an interactive online tool to support parents' genomic testing decisions. J Genet Couns.

[32] Bradbury AR, Lee JW, Gaieski JB, Li S, Gareen IF, Flaherty KT, Herman BA, Domchek SM, DeMichele AM, Maxwell KN, Onitilo AA, Virani S, Park S, Faller BA, Grant SC, Ramaekers RC, Behrens RJ, Nambudiri GS, Carlos RC, Wagner LI (2022). A randomized study of genetic education versus usual care in tumor profiling for advanced cancer in the ECOG-ACRIN Cancer Research Group (EAQ152). Cancer.

[33] Gaieski JB, Patrick-Miller L, Egleston BL, Maxwell KN, Walser S, DiGiovanni L, Brower J, Fetzer D, Ganzak A, McKenna D, Long JM, Powers J, Stopfer JE, Nathanson KL, Domchek SM, Bradbury AR (2019). Research participants' experiences with return of genetic research results and preferences for web-based alternatives. Mol Genet Genomic Med.

