# Supplementary material for: User and Usability Testing of a Web-Based Genetics Education Tool for Parkinson Disease: Mixed Methods Study
Source: JMIR Bioinform Biotechnol. 2023 Aug 30;4:e45370. doi: 10.2196/45370 (PMC11135229; doi:10.2196/45370)
Supplement: Multimedia Appendix 1 [file bioinform_v4i1e45370_app1.pdf]

Multimedia Appendix

Includes:

Table S1.

Table S2.

Table S3.

Table S4.

Questionnaire 1.

Questionnaire 2.

Table S1. Internet use reported by movement disorders specialists (N=11) and people with Parkinson's disease (N=13) in the content review (Phase 1). Available data presented as precent of total cohort.

	Movement Disorders Specialist	Person with Parkinson's Disease
**Internet Use**		
>12 hours per week	9 (69%)	7 (54%)
10-12 hours/week	1 (9%)	3 (23%)
7-9 hours/week	1 (9%)	3 (23%)
**What device do you use for internet access?** (more than once response allowed)		
Smartphone	11 (100%)	13 (100%)
Desktop or Laptop	11 (100%)	11 (85%)
Tablet	6 (55%)	6 (46%)
**Type of internet access** (more than once response allowed)		
Dial-up	0 (0%)	0 (0%)
DSL or Broadband (wired or wi-fi)	11 (100%)	13 (100%)
Cellular network	7 (64%)	6 (46%)
**Comfort with internet use**		
Confident/no need for help	8 (73%)	5 (35%)
Comfortable/rare assistance	3 (27%)	4 (31%)
Need some assistance	0 (0%)	3 (23%)
Uncomfortable/needs assistance	0 (0%)	1 (8%)

Table S2. Average usefulness scale for each page reported by movement disorders specialists (N=11) and people with Parkinson's disease (N=13) in the content review (Phase 1). Usefulness is reported from 1-10 with 1 being the lowest and 10 being the highest. PD = Parkinson's disease. GT = Genetic Testing. VUS = Variant of uncertain significance.

Page	Movement Disorders Specialist	Person with Parkinson's Disease
1. Title	8.36	6.85
2 How to use the website	9.27	8.77
3. Introduction	9.45	8.62
4. Content Summary	9.70	9.15
5. What is PD?	8.55	9.08
6. PD Signs and Symptoms	8.18	8.00
7. Causes of PD	8.64	8.62
8. PD Treatments	7.20	8.62
9. Inheritance	8.36	8.77
10. When to consider GT	9.20	8.23
11. Additional info about GT	7.20	9.17
12. Genetics Introduction	8.73	9.38
13. Types of GT	9.30	8.31
14 Risks and Benefits of GT	9.50	8.62
15. GT Process	9.30	7.62
16. Role of Genetics in PD	9.00	8.85
17. GBA	9.11	8.54
18. LRRK2	9.11	8.82
19. Other PD Genes	8.89	7.82
20 VUS	8.70	8.54
21 How do I get my results	9.22	7.85
22. Implications/limitations	9.10	7.46
23. Conclusion	9.44	9.73
**Average**	8.85	8.49
**Standard Deviation**	0.64	0.65

Table S3. Summary feedback reported by movement disorders specialists (N=11) and people with Parkinson's disease (N=13) in the content review (Phase 1). Available data presented as percent of total cohort.

	Movement Disorders Specialist	Person with Parkinson's Disease
**Website navigation was:**		
'Intuitive and easy to learn and had no problem accessing the information on this site'	6 (55%)	10 (77%)
'the instructions were clear, and I had little to no trouble using this site'	4 (36%)	3 (23%)
**The information found on external links was:**		
Definitely helpful	6 (55%)	7 (54%)
Somewhat helpful	0 (0%)	2 (15%)
Interesting but hard to understand	1 (9%)	0 (15%)
Not very helpful and/or difficult to understand	2 (18%)	0 (15%)
**Access to the 'contact us' page was:**		
Feasible	9 (82%)	10 (77%)
Difficult	0 (0%)	2 (15%)
I was not aware I could make contact to ask questions	1 (9%)	1 (8%)

Table S4. Final remarks from movement disorders specialists (N=11) and people with Parkinson's disease (N=13) in the content review (Phase 1).

Movement Disorders Specialist Feedback				
Page	**Did you feel like the order of the pages made sense? Which order would you like the information presented to you?**	**How do you feel about the interactive tools? Did they help with understanding the material? Do they distract from the material?**	**Did you click on the external links providing additional information on the material presented on a page? If so, how helpful did you think the additional information was? (Scale of 1-4)**	**Please provide any additional feedback**
1	See above, for the most part yes.	They were helpful	4 - definitely helpful	It's great!
2	look above	helpful because it breaks up the info	4 - definitely helpful	No
3	order made sense	overall was helpful but needs tweaks.	1 - not very helpful at all and/or difficult to understand	nope
4	See above.	helpful.	4 - definitely helpful	Have a progress bar. have direction on first page directing the order of actions, listen then read or vice versa.
5	Yes	Helpful	4 - definitely helpful	for pics have 1 AA, 1 Hispanic, 1 SE Asian, 1 East Asian. may want to ask whether someone had to help them navigate through the site and that its okay to have someone there helping through the site
6	Just one page, look above	more helpful, can be more interactive		talk a little more about how to get results back. maybe more obvious method of contact mentions the more personal side of why people choose or don't choose to get genetic testing (testimonials)
7	They made sense	helpful	2 - interesting but hard to understand	main point of this website is to focus on genetics and work backwards from the main point to streamline the website.
8	Yes. autosomal and dominant and recessive should be	helpful	4 - definitely helpful	maybe make contact us more obvious

	moved up earlier, the nature of the testing may also need to move up earlier			
9	Yes	at times they were good but need to be synced	4 - definitely helpful	Make sure purpose is clear, is it for PD genetic testing in general or just for MIND
10				
11	Yes	Helpful	1 - not very helpful at all and/or difficult to understand	make sure we aren't overwhelming patients, let them know how long it's going to take
People with Parkinson's Disease Feedback				
1	Yes	distracting if the text didn't match the audio or if it was hard to read	3 - somewhat helpful	No
2	Yes	Helped		No
3	order made sense	helpful	4 - definitely helpful	have audio more obvious on the page.
4	Yes	a little bit of both		be more selective on information. focus more on privacy
5	Yes	helpful		make the purpose of this study clearer
6	Yes	helpful	4 - definitely helpful	No
7	Yes	Helpful tools	4 - definitely helpful	Make the play button for audio and video bigger
8	Made sense	helpful		make the website flow more smoothly.
9	It made sense	Yes	3 - somewhat helpful	Shorter. May need to cut some out.
10	look above, add a summary	they were fine	4 - definitely helpful	No
11	made sense	helpful but should be more obvious when to scroll to next piece of info	4 - definitely helpful	No
12	Look above	helpful	4 - definitely helpful	No
13	Order was fine	more distracting	4 - definitely helpful	make it more visual appealing

Content Review Questions

Record ID ______________________

INDDID ______________________

First Name ______________________

Last Name ______________________

To start, I want to ask you a couple of general questions about how you use the internet and then more specific questions about the web-based eHealth tool we plan to use

Do you ever go on-line to access the Internet or World Wide Web, or to send and receive e-mail?
- ◯ Yes
- ◯ No

On average, how many hours per week do you spend on or using the internet?
- ◯ 0 - 3 hours per week
- ◯ 4 - 6 hours per week
- ◯ 7 - 9 hours per week
- ◯ 10 - 12 hours per week
- ◯ 12+ hours per week

Please rate your comfort level of using the internet
- ◯ 1 - I often feel confused when navigating online and cannot access the information I want and do not understand what is happening even with assistance
- ◯ 2 - I am uncomfortable using a computer and cannot access information without the assistance of someone else
- ◯ 3 - I can manage to find things on the internet with some assistance
- ◯ 4 - I am comfortable navigating online and rarely need assistance finding information
- ◯ 5 - I feel confident and know how to access information that I want without any assistance

Where do you go to use the Internet? ______________________

What device do you use to access the internet? (please select all that apply)
- ☐ computer (desktop or laptop)
- ☐ smartphone
- ☐ tablet
- ☐ other (please specify below)

please specify what other device you use ______________________

Do you use the Internet from home? ______________________

Where do you use the Internet from most often? ______________________

Which of the following, if any, are the reasons you do not access the Internet?	☐ Because you are not interested.<br>☐ Because it costs too much.<br>☐ Because it is too complicated to use.<br>☐ Because you do not think it is useful.<br>☐ not applicable (i.e. no reasons for not accessing internet)
In the last 12 months, have you used the Internet for any of the following reasons:	☐ Used e-mail or the Internet to communicate with friends or family?<br>☐ Used e-mail or the Internet to communicate with a doctor or doctor's office?<br>☐ Looked for health or medical information for yourself?
When you use the Internet, do you access it through:	☐ A regular dial-up telephone line<br>☐ Broadband such as DSL, cable or FiOS<br>☐ A cellular network (i.e., telephone, 3G/4G)<br>☐ A wireless network (Wi-Fi)<br>☐ Other: Please Specify
please specify if you clicked 'other'	__________________________
If you marked more than one, which do you use most frequently?	○ A regular dial-up telephone line<br>○ Broadband such as DSL, cable or FiOS<br>○ A cellular network (i.e., telephone, 3G/4G)<br>○ A wireless network (Wi-Fi)<br>○ Other: Please Specify
please specify if you clicked 'other'	__________________________

Please complete the table using the scale provided: (adapted from Baker et al, JAMA. 2003;289:2400-2406)
In the Past Year, About How Often Did You:

	Ever in the last Year	More than once a week	about once a week	Once a month	once every 2-3 months	less than once every 2-3 months
Look on the Internet for information or advice about health or health care?	○	○	○	○	○	○
Use e-mail or the Internet to communicate with a doctor or other health care provider?	○	○	○	○	○	○
Use e-mail or the Internet to communicate with a family member or friend about health or health care?	○	○	○	○	○	○

Use e-mail or the Internet to communicate with other people who have health conditions or concerns ○ ○ ○ ○ ○ ○

Next, before we review the site, we would like to gauge your knowledge of genetics and heritability as well as your knowledge on Parkinson's Disease.

What is your level of knowledge of genetics?
- ○ 1 I am not knowledgeable
- ○ 2 I have heard of some terms but don't know what they mean
- ○ 3 I am familiar but no in depth knowledge
- ○ 4 very knowledgeable

What if your level of knowledge on Parkinson's Disease?
- ○ 1 I am not knowledgeable
- ○ 2 I have heard of some terms but don't know what they mean
- ○ 3 I am familiar but no in depth knowledge
- ○ 4 very knowledgeable

What is your level of knowledge on genetic testing?
- ○ 1 I am not knowledgeable
- ○ 2 I have heard of some terms but don't know what they mean
- ○ 3 I am familiar but no in depth knowledge
- ○ 4 very knowledgeable

Thanks. Now I will ask specific questions about the web-based IMAGINE-PD tool we plan to use for the IMAGINE-PD study. We are going to go through each webpage, one at a time. On each, I will ask if there is anything that you don't understand, additional information you think should be included and any ways you think we should modify the screen.

Please take a moment to read the screen. Is there anything that you don't understand? Any words or sections you think other participants might find confusing? ______________________________

Please rate the usefulness of this screen (1 being the least useful and 10 being very useful)
- ○ 1
- ○ 2
- ○ 3
- ○ 4
- ○ 5
- ○ 6
- ○ 7
- ○ 8
- ○ 9
- ○ 10

After looking at this screen, is there any additional information you would want to see? Either on this screen or on the next screen? ______________________________

Is there anything else we should change about the content on this screen? Is there anything we should change about the appearance or the graphics? ______________________________

Do you feel like the page clearly delivered the material?	◯ 1 - Not at all<br>◯ 2 - The information was presented in a confusing way was hard to understand<br>◯ 3 - The information was presented in a confusing way but I was able to Understand clearly<br>◯ 4 - the page clearly portrayed the information

Page 2 (How do I use this site?)

Please take a moment to read the screen. Is there anything that you don't understand? Any words or sections you think other participants might find confusing?	__________________________________
Please rate the usefulness of this screen<br>(1 being the least useful and 10 being very useful)	◯ 1<br>◯ 2<br>◯ 3<br>◯ 4<br>◯ 5<br>◯ 6<br>◯ 7<br>◯ 8<br>◯ 9<br>◯ 10
After looking at this screen, is there any additional information you would want to see? Either on this screen or on the next screen?	__________________________________
Is there anything else we should change about the content on this screen? Is there anything we should change about the appearance or the graphics?	__________________________________
Do you feel like the page clearly delivered the material?	◯ 1 - Not at all<br>◯ 2 - The information was presented in a confusing way was hard to understand<br>◯ 3 - The information was presented in a confusing way but I was able to Understand clearly<br>◯ 4 - the page clearly portrayed the information
What would you expect to hear from the audio based on the title of the page? How useful was the audio to you? Why or why not?	__________________________________

Page 3 (What is IMAGINE PD? Auto Audio)

Please take a moment to read the screen. Is there anything that you don't understand? Any words or sections you think other participants might find confusing?	__________________________________

Please rate the usefulness of this screen<br>(1 being the least useful and 10 being very useful)	◯ 1<br>◯ 2<br>◯ 3<br>◯ 4<br>◯ 5<br>◯ 6<br>◯ 7<br>◯ 8<br>◯ 9<br>◯ 10
After looking at this screen, is there any additional information you would want to see? Either on this screen or on the next screen?	__________________________________
Is there anything else we should change about the content on this screen? Is there anything we should change about the appearance or the graphics?	__________________________________
Do you feel like the page clearly delivered the material?	◯ 1 - Not at all<br>◯ 2 - The information was presented in a confusing way was hard to understand<br>◯ 3 - The information was presented in a confusing way but I was able to Understand clearly<br>◯ 4 - the page clearly portrayed the information
What would you expect to hear from this audio based on the title of the page? How useful was this audio to you? Why or why not?	__________________________________

Page 4 (Summary)

Please take a moment to read the screen. Is there anything that you don't understand? Any words or sections you think other participants might find confusing?	__________________________________
Please rate the usefulness of this screen<br>(1 being the least useful and 10 being very useful)	◯ 1<br>◯ 2<br>◯ 3<br>◯ 4<br>◯ 5<br>◯ 6<br>◯ 7<br>◯ 8<br>◯ 9<br>◯ 10
After looking at this screen, is there any additional information you would want to see? Either on this page or the next?	__________________________________
Is there anything else we should change about the content on this page? Is there anything we should change about the appearance or the graphics?	__________________________________

Do you feel like the page clearly delivered the material?	◯ 1 - Not at all<br>◯ 2 - The information was presented in a confusing way was hard to understand<br>◯ 3 - The information was presented in a confusing way but I was able to Understand clearly<br>◯ 4 - the page clearly portrayed the information
What would you expect to hear from this audio based on the title of the page? How useful was this audio to you? Why or why not?	________________________________

Page 5 (What is PD? Video)

Please take a watch the video. Is there anything that you don't understand? Any words or parts you think other participants might find confusing?	________________________________
Please rate the usefulness of this Video (1 being the least useful and 10 being very useful)	◯ 1 ◯ 2 ◯ 3 ◯ 4<br>◯ 5 ◯ 6 ◯ 7 ◯ 8<br>◯ 9 ◯ 10
After watching the video, is there any additional information you would want to see? Either in the video or on the screen?	________________________________
Is there anything else we should change about the content on the video? Is there anything we should change about the appearance or the graphics?	________________________________
Do you feel like the video clearly delivered the material?	◯ 1 - Not at all<br>◯ 2 - The information was presented in a confusing way was hard to understand<br>◯ 3 - The information was presented in a confusing way but I was able to Understand clearly<br>◯ 4 - the video clearly portrayed the information
What would you expect to see if you clicked on this video? How useful would this video be for you? Why or why not? How likely would it be that you would open and watch this video? Why or why not?	________________________________

Page 6 (How is PD Diagnosed?)

Please take a moment to read the screen. Is there anything that you don't understand? Any words or sections you think other participants might find confusing?	________________________________
Please rate the usefulness of this screen (1 being the least useful and 10 being very useful)	◯ 1<br>◯ 2<br>◯ 3<br>◯ 4<br>◯ 5<br>◯ 6<br>◯ 7<br>◯ 8<br>◯ 9<br>◯ 10

After looking at this screen, is there any additional information you would want to see? Either on this screen or on the next screen?	__________________________________
Is there anything else we should change about the content on this screen? Is there anything we should change about the appearance or the graphics?	__________________________________
Do you feel like the page clearly delivered the material?	◯ 1 - Not at all<br>◯ 2 - The information was presented in a confusing way was hard to understand<br>◯ 3 - The information was presented in a confusing way but I was able to Understand clearly<br>◯ 4 - the page clearly portrayed the information
What would you expect to hear from this audio based on the title of the page? How useful was this audio to you? Why or why not?	__________________________________

Page 7 (What causes PD? Video)

Please take a moment to watch the video. Is there anything that you don't understand? Any words or parts you think other participants might find confusing?	__________________________________
Please rate the usefulness of this screen (1 being the least useful and 10 being very useful)	◯ 1<br>◯ 2<br>◯ 3<br>◯ 4<br>◯ 5<br>◯ 6<br>◯ 7<br>◯ 8<br>◯ 9<br>◯ 10
After watching the video, is there any additional information you would want to see? Either in the video or on the screen?	__________________________________
Is there anything else we should change about the content on the video? Is there anything we should change about the appearance or the graphics?	__________________________________
Do you feel like the video clearly delivered the material?	◯ 1 - Not at all<br>◯ 2 - The information was presented in a confusing way was hard to understand<br>◯ 3 - The information was presented in a confusing way but I was able to Understand clearly<br>◯ 4 - the video clearly portrayed the information
What would you expect to see if you clicked on this video? How useful would this video be for you? Why or why not? How likely would it be that you would open and watch this video? Why or why not?	__________________________________

Page 8 (How is PD Treated?)

Please take a moment to read the screen. Is there anything that you don't understand? Any words or sections you think other participants might find confusing?

____

Please rate the usefulness of this screen
(1 being the least useful and 10 being very useful)

- ◯ 1
- ◯ 2
- ◯ 3
- ◯ 4
- ◯ 5
- ◯ 6
- ◯ 7
- ◯ 8
- ◯ 9
- ◯ 10

After looking at this screen, is there any additional information you would want to see? Either on this screen or on the next screen?

____

Is there anything else we should change about the content on this screen? Is there anything we should change about the appearance or the graphics?

____

Do you feel like the page clearly delivered the material?

- ◯ 1 - Not at all
- ◯ 2 - The information was presented in a confusing way was hard to understand
- ◯ 3 - The information was presented in a confusing way but I was able to Understand clearly
- ◯ 4 - the page clearly portrayed the information

What would you expect to hear from this audio based on the title of the page? How useful was this audio to you? Why or why not?

____

Page 9 (What is Genetics? Video)

Please take a moment to watch the video. Is there anything that you don't understand? Any words or sections you think other participants might find confusing?

____

Please rate the usefulness of this screen
(1 being the least useful and 10 being very useful)

- ◯ 1
- ◯ 2
- ◯ 3
- ◯ 4
- ◯ 5
- ◯ 6
- ◯ 7
- ◯ 8
- ◯ 9
- ◯ 10

After watching the video, is there any additional information you would want to see? Either in the video or on the screen?

____

Is there anything else we should change about the content on the video? Is there anything we should change about the appearance or the graphics?

____

Do you feel like the video clearly delivered the material?

- ◯ 1 - Not at all
- ◯ 2 - The information was presented in a confusing way was hard to understand
- ◯ 3 - The information was presented in a confusing way but I was able to Understand clearly
- ◯ 4 - the video clearly portrayed the information

What would you expect to see if you clicked on this video? How useful would this video be for you? Why or why not? How likely would it be that you would open and watch this video? Why or why not?

____

Page 10 (Why should I have genetic testing?)

Please take a moment to read the screen. Is there anything that you don't understand? Any words or sections you think other participants might find confusing?

____

Please rate the usefulness of this screen
(1 being the least useful and 10 being very useful)

- ◯ 1
- ◯ 2
- ◯ 3
- ◯ 4
- ◯ 5
- ◯ 6
- ◯ 7
- ◯ 8
- ◯ 9
- ◯ 10

After looking at this screen, is there any additional information you would want to see? Either on this screen or on the next screen?

____

Is there anything else we should change about the content on this screen? Is there anything we should change about the appearance or the graphics?

____

Do you feel like the page clearly delivered the material?

- ◯ 1 - Not at all
- ◯ 2 - The information was presented in a confusing way was hard to understand
- ◯ 3 - The information was presented in a confusing way but I was able to Understand clearly
- ◯ 4 - the page clearly portrayed the information

What would you expect to hear from this audio based on the title of the page? How useful was this audio to you? Why or why not?

____

Page 10.5 (Types of Genetic Tests)

Please take a moment to read the screen. Is there anything that you don't understand? Any words or sections you think other participants might find confusing?

____

Please rate the usefulness of this screen
(1 being the least useful and 10 being very useful)

- ◯ 1
- ◯ 2
- ◯ 3
- ◯ 4
- ◯ 5
- ◯ 6
- ◯ 7
- ◯ 8
- ◯ 9
- ◯ 10

After looking at this screen, is there any additional information you would want to see? Either on this screen or on the next screen?

____

Is there anything else we should change about the content on this screen? Is there anything we should change about the appearance or the graphics?

____

Do you feel like the page clearly delivered the material?

- ◯ 1 - Not at all
- ◯ 2 - The information was presented in a confusing way was hard to understand
- ◯ 3 - The information was presented in a confusing way but I was able to Understand clearly
- ◯ 4 - the page clearly portrayed the information

Page 11 (Genetics 101. Video)

Please take a moment to watch the video. Is there anything that you don't understand? Any words or parts you think other participants might find confusing?

____

Please rate the usefulness of this screen
(1 being the least useful and 10 being very useful)

- ◯ 1
- ◯ 2
- ◯ 3
- ◯ 4
- ◯ 5
- ◯ 6
- ◯ 7
- ◯ 8
- ◯ 9
- ◯ 10

After watching the video, is there any additional information you would want to see? Either in the video or on the screen?

____

Is there anything else we should change about the content on this video? Is there anything we should change about the appearance or the graphics?

____

Do you feel like the video clearly delivered the material?	◯ 1 - Not at all<br>◯ 2 - The information was presented in a confusing way was hard to understand<br>◯ 3 - The information was presented in a confusing way but I was able to Understand clearly<br>◯ 4 - the video clearly portrayed the information
What would you expect to see if you clicked on this video? How useful would this video be for you? Why or why not? How likely would it be that you would open and watch this video? Why or why not?	__________________________________

Page 12 (What is genetic testing?)

Please take a moment to read the screen. Is there anything that you don't understand? Any words or sections you think other participants might find confusing?	__________________________________
Please rate the usefulness of this screen<br>(1 being the least useful and 10 being very useful)	◯ 1<br>◯ 2<br>◯ 3<br>◯ 4<br>◯ 5<br>◯ 6<br>◯ 7<br>◯ 8<br>◯ 9<br>◯ 10
After looking at this screen, is there any additional information you would want to see? Either on this screen or on the next screen?	__________________________________
Is there anything else we should change about the content on this screen? Is there anything we should change about the appearance or the graphics?	__________________________________
Do you feel like the page clearly delivered the material?	◯ 1 - Not at all<br>◯ 2 - The information was presented in a confusing way was hard to understand<br>◯ 3 - The information was presented in a confusing way but I was able to Understand clearly<br>◯ 4 - the page clearly portrayed the information
What would you expect to hear from this audio based on the title of the page? How useful was this audio to you? Why or why not?	__________________________________

Page 13 (Benefits and Risks)

Please take a moment to read the screen. Is there anything that you don't understand? Any words or sections you think other participants might find confusing?

____

Please rate the usefulness of this screen (1 being the least useful and 10 being very useful)

◯ 1
◯ 2
◯ 3
◯ 4
◯ 5
◯ 6
◯ 7
◯ 8
◯ 9
◯ 10

After looking at this screen, is there any additional information you would want to see? Either on this screen or on the next screen?

____

Is there anything else we should change about the content on this screen? Is there anything we should change about the appearance or the graphics?

____

Do you feel like the page clearly delivered the material?

◯ 1 - Not at all
◯ 2 - The information was presented in a confusing way was hard to understand
◯ 3 - The information was presented in a confusing way but I was able to Understand clearly
◯ 4 - the page clearly portrayed the information

What would you expect to hear from this audio based on the title of the page? How useful was this audio to you? Why or why not?

____

Page 14 (Genetic Testing Process)

Please take a moment to read the screen. Is there anything that you don't understand? Any words or sections you think other participants might find confusing?

____

Please rate the usefulness of this screen (1 being the least useful and 10 being very useful)

◯ 1
◯ 2
◯ 3
◯ 4
◯ 5
◯ 6
◯ 7
◯ 8
◯ 9
◯ 10

After looking at this screen, is there any additional information you would want to see? Either on this screen or on the next screen?

____

Is there anything else we should change about the content on this screen? Is there anything we should change about the appearance or the graphics?	__________________________________
Do you feel like the page clearly delivered the material?	◯ 1 - Not at all<br>◯ 2 - The information was presented in a confusing way was hard to understand<br>◯ 3 - The information was presented in a confusing way but I was able to Understand clearly<br>◯ 4 - the page clearly portrayed the information
What would you expect to hear from this audio based on the title of the page? How useful was this audio to you? Why or why not?	__________________________________

Page 15 (How does Genetics affect PD? Video)

Please take a moment to watch the video. Is there anything that you don't understand? Any words or parts you think other participants might find confusing?	__________________________________
Please rate the usefulness of this screen (1 being the least useful and 10 being very useful)	◯ 1<br>◯ 2<br>◯ 3<br>◯ 4<br>◯ 5<br>◯ 6<br>◯ 7<br>◯ 8<br>◯ 9<br>◯ 10
After watching the video, is there any additional information you would want to see? Either in the video or on screen?	__________________________________
Is there anything else we should change about the content on the video? Is there anything we should change about the appearance or the graphics?	__________________________________
Do you feel like the video clearly delivered the material?	◯ 1 - Not at all<br>◯ 2 - The information was presented in a confusing way was hard to understand<br>◯ 3 - The information was presented in a confusing way but I was able to Understand clearly<br>◯ 4 - the video clearly portrayed the information
What would you expect to see if you clicked on this video? How useful would this video be for you? Why or why not? How likely would it be that you would open and watch this video? Why or why not?	__________________________________

Page 16 (How does GBA affect PD?)

Please take a moment to read the screen. Is there anything that you don't understand? Any words or sections you think other participants might find confusing?	__________________________________
Please rate the usefulness of this screen (1 being the least useful and 10 being very useful)	◯ 1<br>◯ 2<br>◯ 3<br>◯ 4<br>◯ 5<br>◯ 6<br>◯ 7<br>◯ 8<br>◯ 9<br>◯ 10
After looking at this screen, is there any additional information you would want to see? Either on this screen or on the next screen?	__________________________________
Is there anything else we should change about the content on this screen? Is there anything we should change about the appearance or the graphics?	__________________________________
Do you feel like the page clearly delivered the material?	◯ 1 - Not at all<br>◯ 2 - The information was presented in a confusing way was hard to understand<br>◯ 3 - The information was presented in a confusing way but I was able to Understand clearly<br>◯ 4 - the page clearly portrayed the information
What would you expect to hear from this audio based on the title of the page? How useful was this audio to you? Why or why not?	__________________________________

Page 17 (How does LRRK2 affect PD?)

Please take a moment to read the screen. Is there anything that you don't understand? Any words or sections you think other participants might find confusing?	__________________________________
Please rate the usefulness of this screen (1 being the least useful and 10 being very useful)	◯ 1<br>◯ 2<br>◯ 3<br>◯ 4<br>◯ 5<br>◯ 6<br>◯ 7<br>◯ 8<br>◯ 9<br>◯ 10
After looking at this screen, is there any additional information you would want to see? Either on this screen or on the next screen?	__________________________________

Is there anything else we should change about the content on this screen? Is there anything we should change about the appearance or the graphics?	__________________________________
Do you feel like the page clearly delivered the material?	◯ 1 - Not at all<br>◯ 2 - The information was presented in a confusing way was hard to understand<br>◯ 3 - The information was presented in a confusing way but I was able to Understand clearly<br>◯ 4 - the page clearly portrayed the information
What would you expect to hear from this audio based on the title of the page? How useful was this audio to you? Why or why not?	__________________________________

Page 18 (Other rare genetic causes of PD)

Please take a moment to read the screen. Is there anything that you don't understand? Any words or sections you think other participants might find confusing?	__________________________________
Please rate the usefulness of this screen<br>(1 being the least useful and 10 being very useful)	◯ 1<br>◯ 2<br>◯ 3<br>◯ 4<br>◯ 5<br>◯ 6<br>◯ 7<br>◯ 8<br>◯ 9<br>◯ 10
After looking at this screen, is there any additional information you would want to see? Either on this screen or on the next screen?	__________________________________
Is there anything else we should change about the content on this screen? Is there anything we should change about the appearance or the graphics?	__________________________________
Do you feel like the page clearly delivered the material?	◯ 1 - Not at all<br>◯ 2 - The information was presented in a confusing way was hard to understand<br>◯ 3 - The information was presented in a confusing way but I was able to Understand clearly<br>◯ 4 - the page clearly portrayed the information
What would you expect to hear from this audio based on the title of the page? How useful was this audio to you? Why or why not?	__________________________________

Page 19 (How do I get my results? Video)

Please take a moment to watch the video. Is there anything that you don't understand? Any words or parts you think other participants might find confusing?

____

Please rate the usefulness of this screen
(1 being the least useful and 10 being very useful)

- ◯ 1
- ◯ 2
- ◯ 3
- ◯ 4
- ◯ 5
- ◯ 6
- ◯ 7
- ◯ 8
- ◯ 9
- ◯ 10

After watching the video, is there any additional information you would want to see? Either in the video or on the screen?

____

Is there anything else we should change about the content on the video? Is there anything we should change about the appearance or the graphics?

____

Do you feel like the video clearly delivered the material?

- ◯ 1 - Not at all
- ◯ 2 - The information was presented in a confusing way was hard to understand
- ◯ 3 - The information was presented in a confusing way but I was able to Understand clearly
- ◯ 4 - the video clearly portrayed the information

What would you expect to see if you clicked on this video? How useful would this video be for you? Why or why not? How likely would it be that you would open and watch this video? Why or why not?

____

Page 20 (Benign variants and VUS)

Please take a moment to read the screen. Is there anything that you don't understand? Any words or sections you think other participants might find confusing?

____

Please rate the usefulness of this screen
(1 being the least useful and 10 being very useful)

- ◯ 1
- ◯ 2
- ◯ 3
- ◯ 4
- ◯ 5
- ◯ 6
- ◯ 7
- ◯ 8
- ◯ 9
- ◯ 10

After looking at this screen, is there any additional information you would want to see? Either on this screen or on the next screen?

____

Is there anything else we should change about the content on this screen? Is there anything we should change about the appearance or the graphics?	______________________
Do you feel like the page clearly delivered the material?	◯ 1 - Not at all<br>◯ 2 - The information was presented in a confusing way was hard to understand<br>◯ 3 - The information was presented in a confusing way but I was able to Understand clearly<br>◯ 4 - the page clearly portrayed the information
What would you expect to hear from this audio based on the title of the page? How useful was this audio to you? Why or why not?	______________________

Page 21 (Implications of results. Video)

Please take a moment to watch the video. Is there anything that you don't understand? Any words or parts you think other participants might find confusing?	______________________
Please rate the usefulness of this screen<br>(1 being the least useful and 10 being very useful)	◯ 1<br>◯ 2<br>◯ 3<br>◯ 4<br>◯ 5<br>◯ 6<br>◯ 7<br>◯ 8<br>◯ 9<br>◯ 10
After watching the video, is there any additional information you would want to see? Either in the video or on the screen?	______________________
Is there anything else we should change about the content on the video? Is there anything we should change about the appearance or the graphics?	______________________
Do you feel like the video clearly delivered the material?	◯ 1 - Not at all<br>◯ 2 - The information was presented in a confusing way was hard to understand<br>◯ 3 - The information was presented in a confusing way but I was able to Understand clearly<br>◯ 4 - the video clearly portrayed the information
What would you expect to see if you clicked on this video? How useful would this video be for you? Why or why not? How likely would it be that you would open and watch this video? Why or why not?	______________________

Page 22 (Conclusion)

Please take a moment to read the screen. Is there anything that you don't understand? Any words or sections you think other participants might find confusing?

____

Please rate the usefulness of this screen
(1 being the least useful and 10 being very useful)

- ◯ 1
- ◯ 2
- ◯ 3
- ◯ 4
- ◯ 5
- ◯ 6
- ◯ 7
- ◯ 8
- ◯ 9
- ◯ 10

After looking at this screen, is there any additional information you would want to see? Either on this screen or on the next screen?

____

Is there anything else we should change about the content on this screen? Is there anything we should change about the appearance or the graphics?

____

Do you feel like the page clearly delivered the material?

- ◯ 1 - Not at all
- ◯ 2 - The information was presented in a confusing way was hard to understand
- ◯ 3 - The information was presented in a confusing way but I was able to Understand clearly
- ◯ 4 - the page clearly portrayed the information

Now we would like to ask you just a few more questions about your overall thoughts on the website

Please rate how difficult it was to navigate through this site:

- ◯ 1 - I had an extremely difficult time and could not get to the material I wanted to learn
- ◯ 2 - It was difficult to interact with the website and the instructions were unclear on how to navigate
- ◯ 3 - The instructions were clear but I still did not have an easy time navigating the site
- ◯ 4 - The instructions were clear and I had little to no trouble using this site
- ◯ 5 - The site was intuitive and easy to learn and had no problem accessing the information on this site

Did you feel like the instructions on how to use this website helped you navigate through the website?

- ◯ Yes
- ◯ No

Did you feel like the order of the pages made sense? Which order would you like the information presented to you?

____

How do you feel about the interactive tools (buttons, menus, links etc) on the screen? Do they help with understanding the material? Do they distract from the material?	__________________________________
Did you click on the external links providing additional information on the material presented on a page? If so, how helpful did you think the additional information was?	◯ 1 - not very helpful at all and/or difficult to understand<br>◯ 2 - interesting but hard to understand<br>◯ 3 - somewhat helpful<br>◯ 4 - definitely helpful
If you had questions while going through the website, how easy was it for you to get to the 'contact us' page and submit an inquiry?	◯ 1 - very difficult, I was not aware I could make contact to ask questions<br>◯ 2 - difficult, I wanted to ask questions but did not know how<br>◯ 3 - feasible, I wanted to ask questions and
Please provide any additional feedback	__________________________________

Usability Questions

Record ID	__________________________________
1. Do you ever go on-line to access the Internet or World Wide Web, or to send and receive e-mail?	◯ Yes<br>◯ No
Where do you go to use the Internet?	__________________________________
Do you use the internet from home?	__________________________________
Where do you use the internet from most often?	__________________________________
Which of the following, if any, are the reasons you do not access the Internet?	◯ Because you are not interested.<br>◯ Because it costs too much.<br>◯ Because it is too complicated to use.<br>◯ Because you do not think it is useful.<br>◯ not applicable (i.e. no reasons for not accessing internet)
2. In the last 12 months, have you used the Internet for any of the following reasons	☐ Used e-mail or the Internet to communicate with a doctor or doctor's office?<br>☐ Looked for health or medical information for yourself?
3. When you use the Internet, do you access it through:	☐ A regular dial-up telephone line<br>☐ Broadband such as DSL, cable or FiOS<br>☐ A cellular network (i.e., telephone, 3G/4G)<br>☐ A wireless network (Wi-Fi)<br>☐ Other: Please Specif
Please Specify	__________________________________
3a. If you marked more than one, which do you use most frequently?	◯ A regular dial-up telephone line<br>◯ Broadband such as DSL, cable or FiOS<br>◯ A cellular network (i.e., telephone, 3G/4G)<br>◯ A wireless network (Wi-Fi)<br>◯ Other: Please Specify
Please Specify	__________________________________

Thanks. Now I will ask for specific feedback on the usability of this portal. Can you click the link we've provided? As you go from page to page, I'm going to ask you some questions.

What did you like best about this page? What did you like least? __________________________________

Is the information understandable. If no, what should be clarified? __________________________________

How do you feel about the fonts, colors and images? __________________________________

Did the page load quickly enough? __________________________________

How easy is navigating around this page and/or using all the buttons? __________________________________

Did you feel like you knew what to do next from this page? __________________________________

How could we improve this page? __________________________________

Any other comments? __________________________________

Page 1

What did you like best about this page? What did you like least? __________________________________

Is the information understandable. If no, what should be clarified? __________________________________

How do you feel about the fonts, colors and images? __________________________________

Did the page load quickly enough? __________________________________

How easy is navigating around this page and/or using all the buttons? __________________________________

Did you feel like you knew what to do next from this page? __________________________________

How could we improve this page? __________________________________

Any other comments? __________________________________

Page 2

What did you like best about this page? What did you like least?	__________________________
Is the information understandable. If no, what should be clarified?	__________________________
How do you feel about the fonts, colors and images?	__________________________
Did the page load quickly enough?	__________________________
How easy is navigating around this page and/or using all the buttons?	__________________________
Did you feel like you knew what to do next from this page?	__________________________
How could we improve this page?	__________________________
Any other comments?	__________________________

Page 3

What did you like best about this page? What did you like least?	__________________________
Is the information understandable. If no, what should be clarified?	__________________________
How do you feel about the fonts, colors and images?	__________________________
Did the page load quickly enough?	__________________________
How easy is navigating around this page and/or using all the buttons?	__________________________
Did you feel like you knew what to do next from this page?	__________________________
How could we improve this page?	__________________________
Any other comments?	__________________________

Page 4

What did you like best about this page? What did you like least?	______________________________
Is the information understandable. If no, what should be clarified?	______________________________
How do you feel about the fonts, colors and images?	______________________________
Did the page load quickly enough?	______________________________
How easy is navigating around this page and/or using all the buttons?	______________________________
Did you feel like you knew what to do next from this page?	______________________________
How could we improve this page?	______________________________
Any other comments?	______________________________

Page 5

What did you like best about this page? What did you like least?	______________________________
Is the information understandable. If no, what should be clarified?	______________________________
How do you feel about the fonts, colors and images?	______________________________
Did the page load quickly enough?	______________________________
How easy is navigating around this page and/or using all the buttons?	______________________________
Did you feel like you knew what to do next from this page?	______________________________
How could we improve this page?	______________________________
Any other comments?	______________________________

Page 6

What did you like best about this page? What did you like least?	______________________
Is the information understandable. If no, what should be clarified?	______________________
How do you feel about the fonts, colors and images?	______________________
Did the page load quickly enough?	______________________
How easy is navigating around this page and/or using all the buttons?	______________________
Did you feel like you knew what to do next from this page?	______________________
How could we improve this page?	______________________
Any other comments?	______________________

Page 7

What did you like best about this page? What did you like least?	______________________
Is the information understandable. If no, what should be clarified?	______________________
How do you feel about the fonts, colors and images?	______________________
Did the page load quickly enough?	______________________
How easy is navigating around this page and/or using all the buttons?	______________________
Did you feel like you knew what to do next from this page?	______________________
How could we improve this page?	______________________
Any other comments?	______________________

**Page 8**	
What did you like best about this page? What did you like least?	______________________
Is the information understandable. If no, what should be clarified?	______________________
How do you feel about the fonts, colors and images?	______________________
Did the page load quickly enough?	______________________
How easy is navigating around this page and/or using all the buttons?	______________________
Did you feel like you knew what to do next from this page?	______________________
How could we improve this page?	______________________
Any other comments?	______________________

**Page 9**	
What did you like best about this page? What did you like least?	______________________
Is the information understandable. If no, what should be clarified?	______________________
How do you feel about the fonts, colors and images?	______________________
Did the page load quickly enough?	______________________
How easy is navigating around this page and/or using all the buttons?	______________________
Did you feel like you knew what to do next from this page?	______________________
How could we improve this page?	______________________
Any other comments?	______________________

Page 10

What did you like best about this page? What did you like least?	__________________
Is the information understandable. If no, what should be clarified?	__________________
How do you feel about the fonts, colors and images?	__________________
Did the page load quickly enough?	__________________
How easy is navigating around this page and/or using all the buttons?	__________________
Did you feel like you knew what to do next from this page?	__________________
How could we improve this page?	__________________
Any other comments?	__________________

Page 11

What did you like best about this page? What did you like least?	__________________
Is the information understandable. If no, what should be clarified?	__________________
How do you feel about the fonts, colors and images?	__________________
Did the page load quickly enough?	__________________
How easy is navigating around this page and/or using all the buttons?	__________________
Did you feel like you knew what to do next from this page?	__________________
How could we improve this page?	__________________
Any other comments?	__________________

Page 12

What did you like best about this page? What did you like least? ______________________

Is the information understandable. If no, what should be clarified? ______________________

How do you feel about the fonts, colors and images? ______________________

Did the page load quickly enough? ______________________

How easy is navigating around this page and/or using all the buttons? ______________________

Did you feel like you knew what to do next from this page? ______________________

How could we improve this page? ______________________

Any other comments? ______________________

Page 13

What did you like best about this page? What did you like least? ______________________

Is the information understandable. If no, what should be clarified? ______________________

How do you feel about the fonts, colors and images? ______________________

Did the page load quickly enough? ______________________

How easy is navigating around this page and/or using all the buttons? ______________________

Did you feel like you knew what to do next from this page? ______________________

How could we improve this page? ______________________

Any other comments? ______________________

Page 14

What did you like best about this page? What did you like least?	____________________
Is the information understandable. If no, what should be clarified?	____________________
How do you feel about the fonts, colors and images?	____________________
Did the page load quickly enough?	____________________
How easy is navigating around this page and/or using all the buttons?	____________________
Did you feel like you knew what to do next from this page?	____________________
How could we improve this page?	____________________
Any other comments?	____________________

Page 15

What did you like best about this page? What did you like least?	____________________
Is the information understandable. If no, what should be clarified?	____________________
How do you feel about the fonts, colors and images?	____________________
Did the page load quickly enough?	____________________
How easy is navigating around this page and/or using all the buttons?	____________________
Did you feel like you knew what to do next from this page?	____________________
How could we improve this page?	____________________
Any other comments?	____________________

Page 16

What did you like best about this page? What did you like least?	__________________
Is the information understandable. If no, what should be clarified?	__________________
How do you feel about the fonts, colors and images?	__________________
Did the page load quickly enough?	__________________
How easy is navigating around this page and/or using all the buttons?	__________________
Did you feel like you knew what to do next from this page?	__________________
How could we improve this page?	__________________
Any other comments?	__________________

Page 17

What did you like best about this page? What did you like least?	__________________
Is the information understandable. If no, what should be clarified?	__________________
How do you feel about the fonts, colors and images?	__________________
Did the page load quickly enough?	__________________
How easy is navigating around this page and/or using all the buttons?	__________________
Did you feel like you knew what to do next from this page?	__________________
How could we improve this page?	__________________
Any other comments?	__________________

Page 18

What did you like best about this page? What did you like least?	______________________
Is the information understandable. If no, what should be clarified?	______________________
How do you feel about the fonts, colors and images?	______________________
Did the page load quickly enough?	______________________
How easy is navigating around this page and/or using all the buttons?	______________________
Did you feel like you knew what to do next from this page?	______________________
How could we improve this page?	______________________
Any other comments?	______________________

Page 19

What did you like best about this page? What did you like least?	______________________
Is the information understandable. If no, what should be clarified?	______________________
How do you feel about the fonts, colors and images?	______________________
Did the page load quickly enough?	______________________
How easy is navigating around this page and/or using all the buttons?	______________________
Did you feel like you knew what to do next from this page?	______________________
How could we improve this page?	______________________
Any other comments?	______________________
