# Supplementary material for: User and Usability Testing of a Web-Based Genetics Education Tool for Parkinson Disease: Mixed Methods Study
Source: JMIR Bioinform Biotechnol. 2023 Aug 30;4:e45370. doi: 10.2196/45370 (PMC11135229; doi:10.2196/45370)
Supplement: Multimedia Appendix 2 [file bioinform_v4i1e45370_app2.pdf]

IMAGINEPD

Please Login:

Participant ID:

Access Code:

Login

Forgot your login information? Contact a study staff member at 215-829-8560 for assistance. Thank you.

IMAGINE-PD
Interactive Multimedia Approach to Genetic Counseling to INform and Educate in Parkinson's Disease

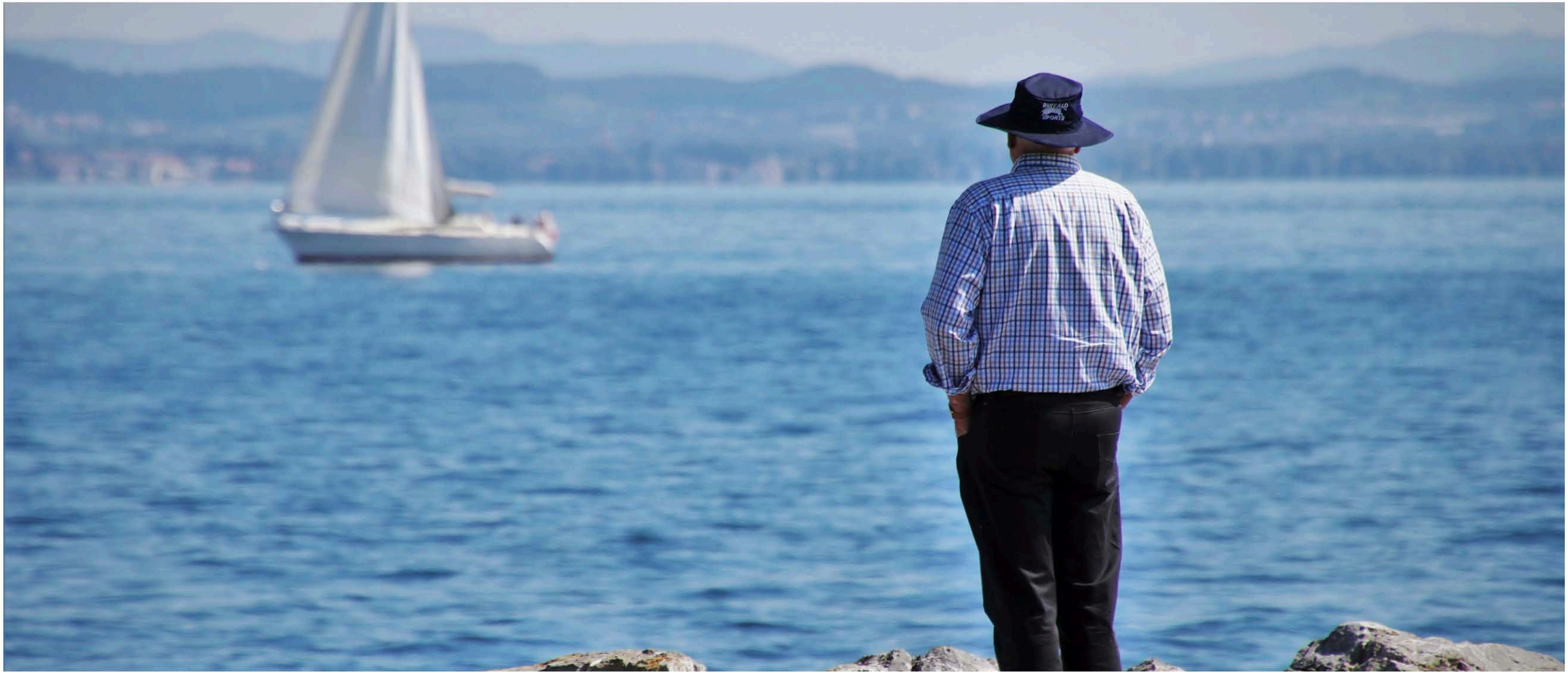

View Glossary Page

What is IMAGINE-PD?

IMAGINE-PD is an online, interactive, genetic counseling tool. It is part of a research study about genetic counseling and genetic testing for Parkinson's disease.

This tool will be for people who have or are at risk for Parkinson's disease.

You may visit this site as many times as you want. You may review the information in any order you prefer. There are optional links for additional resources.

Some pages have text behind the photos. If you are using a laptop, you can "hover" or "click" to view the text. If you are using a mobile device or tablet, you may "zoom in" as shown here.

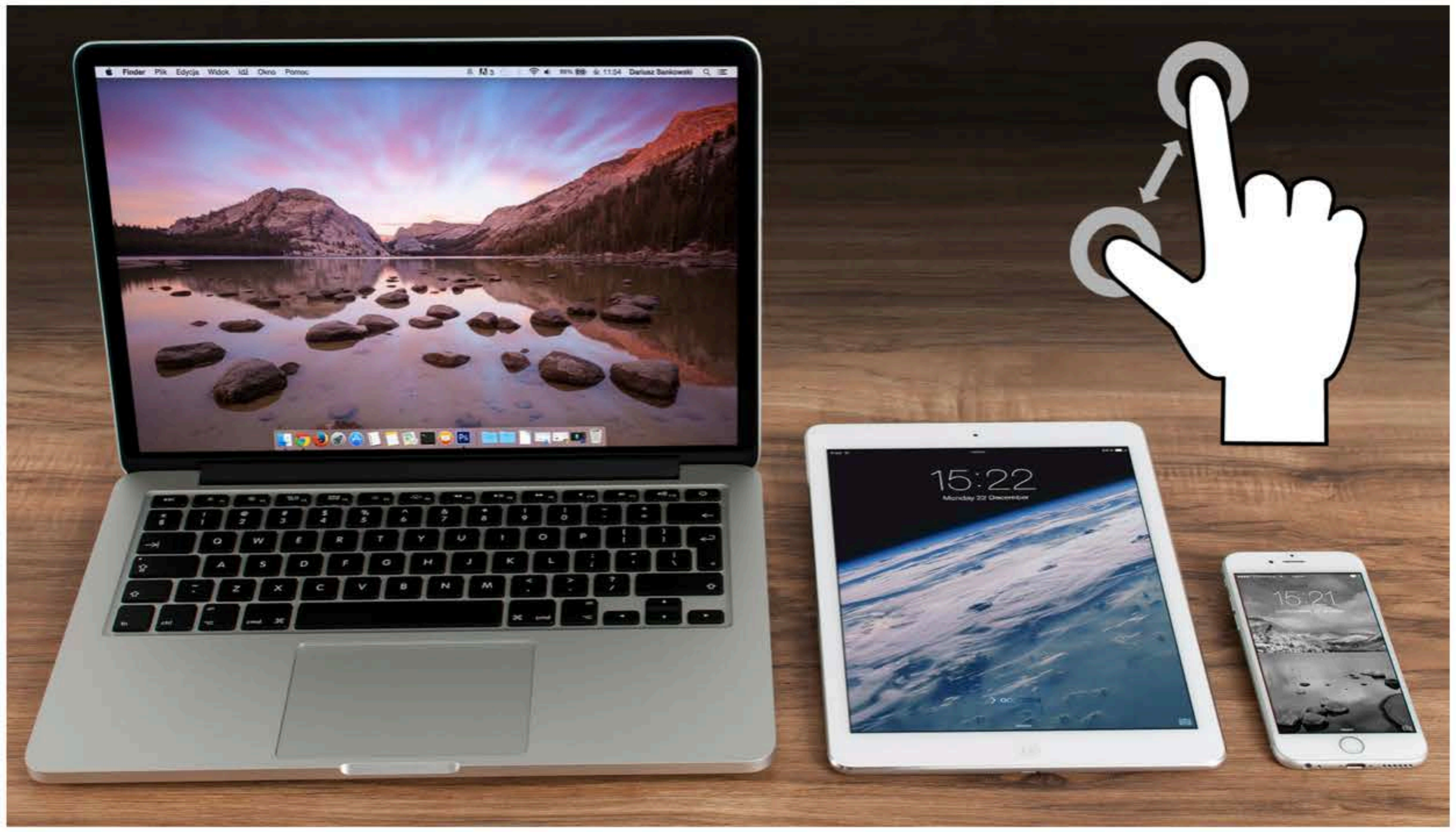

This website will provide you with pre-test genetic counseling. It will take the place of meeting with a counselor prior to testing.

If you have questions about the study please ask the research staff or click the "contact us" button at the bottom of every page.

What is Genetic Counseling?

Genetic counseling typically involves meeting with genetic counselors. Genetic Counselors are healthcare professionals that are trained in genetics.

Genetic counselors and patients may discuss:

- What **type of genetic testing** would be most informative.
- What are some **benefits** and **risks** of genetic testing.
- What genetic testing results **can (and cannot) tell you**.
- What are some **implications** of the test results.
- And later (if you choose to have testing), **what the results are and what they could mean** for you and your family.

Click here to learn more about genetic counseling.

As part of the GET-VIRTUAL PD research study, participants will be randomly assigned to different types of genetic counseling. Then, they can decide whether they would like to learn their genetic test results. **This website will provide you with pretest genetic counseling.** It will take the place of meeting with a genetic counselor prior to genetic testing.

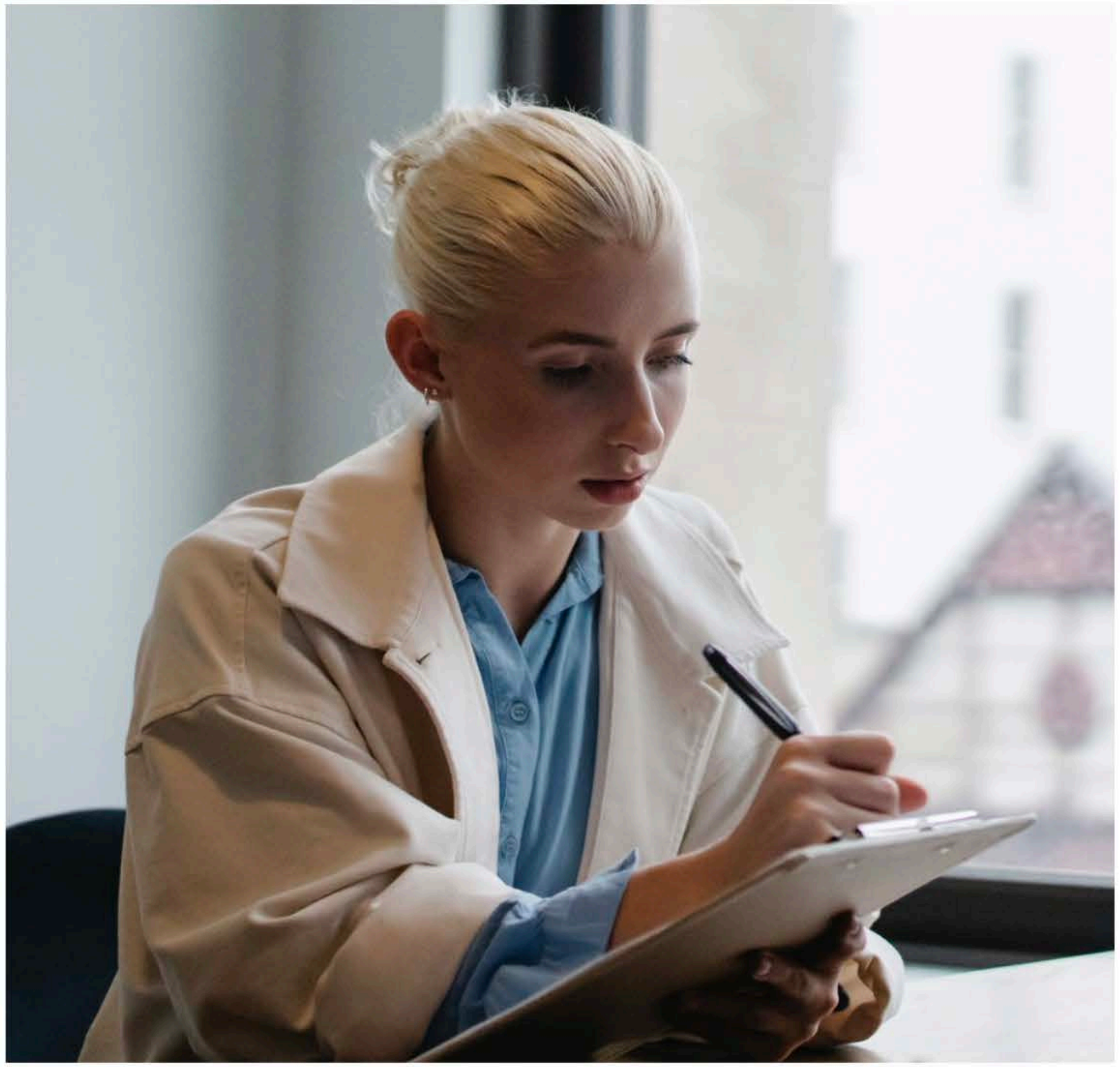

What Is Parkinson's Disease?

This slide is considered essential information for genetic counseling.

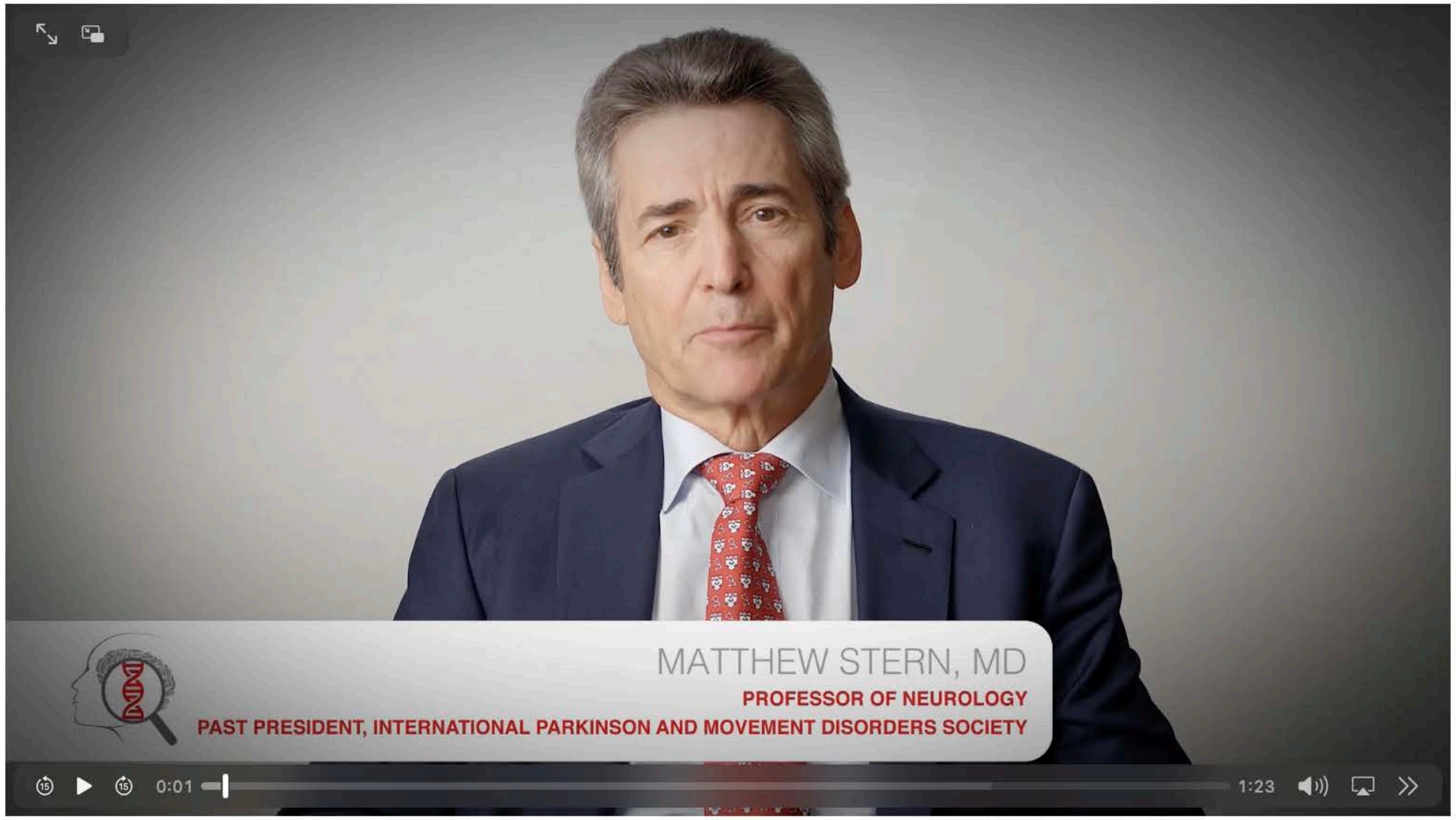

If you would like to read the information provided click *HERE*

Diagnosis, Symptoms, and Treatment

Hover over the pictures below to read about the diagnosis, symptoms, and treatment of Parkinson's disease. If you are using a mobile device or tablet, you may touch the image to reveal the text.

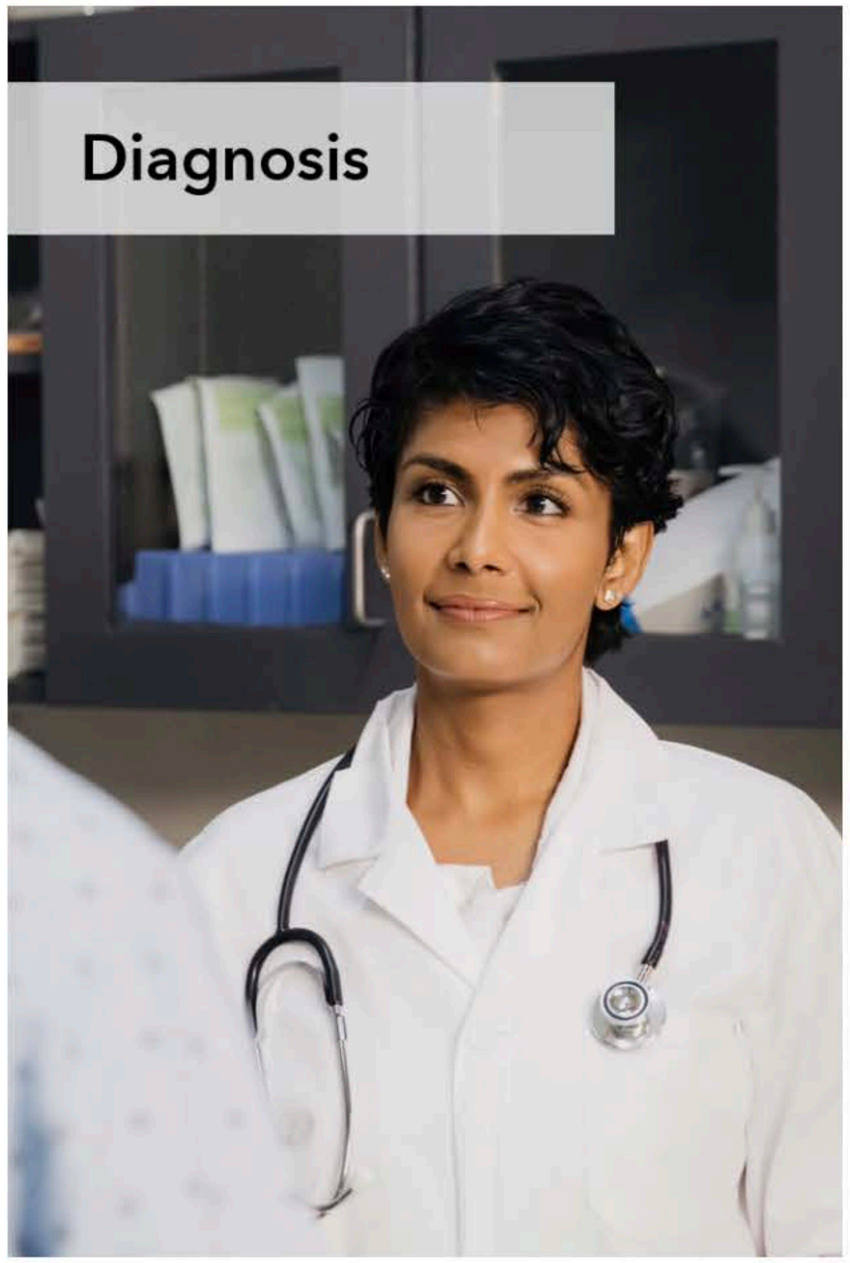

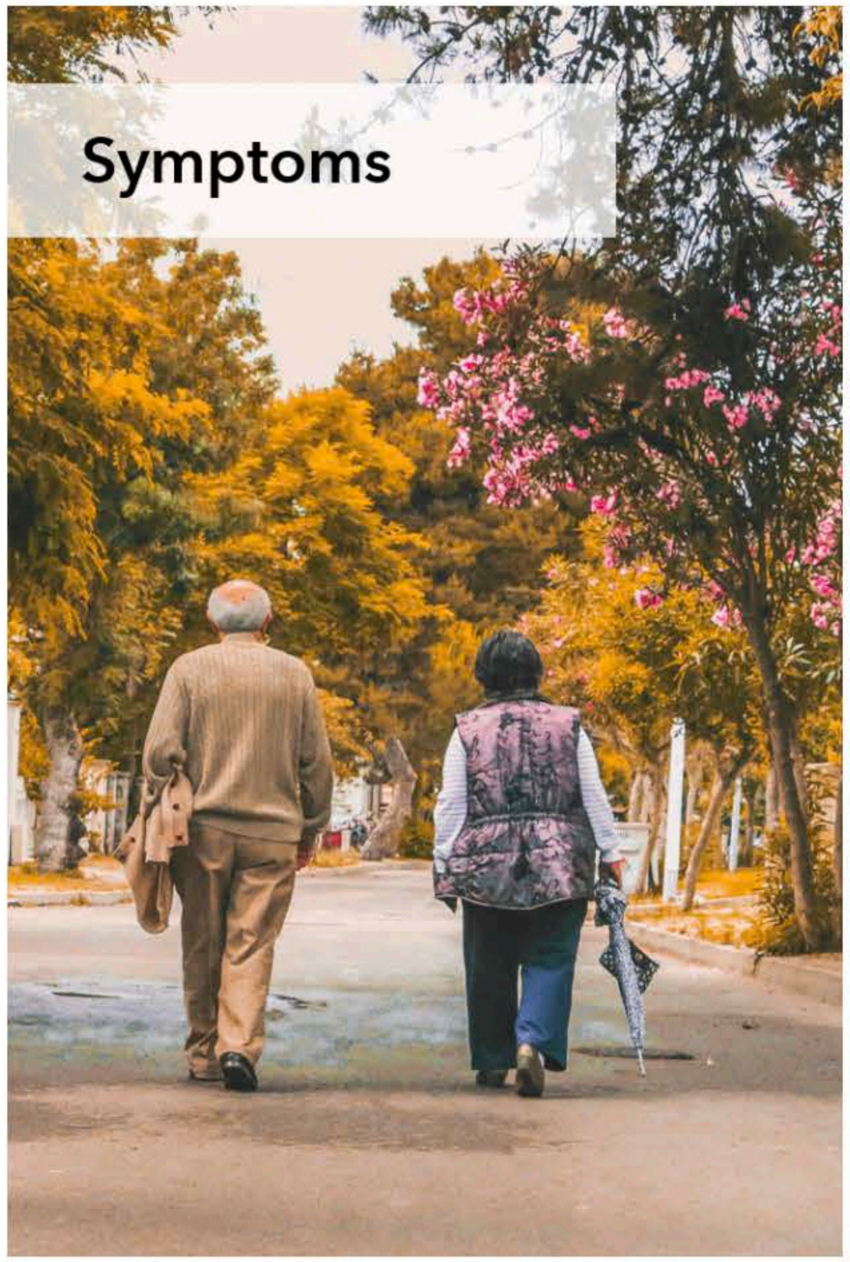

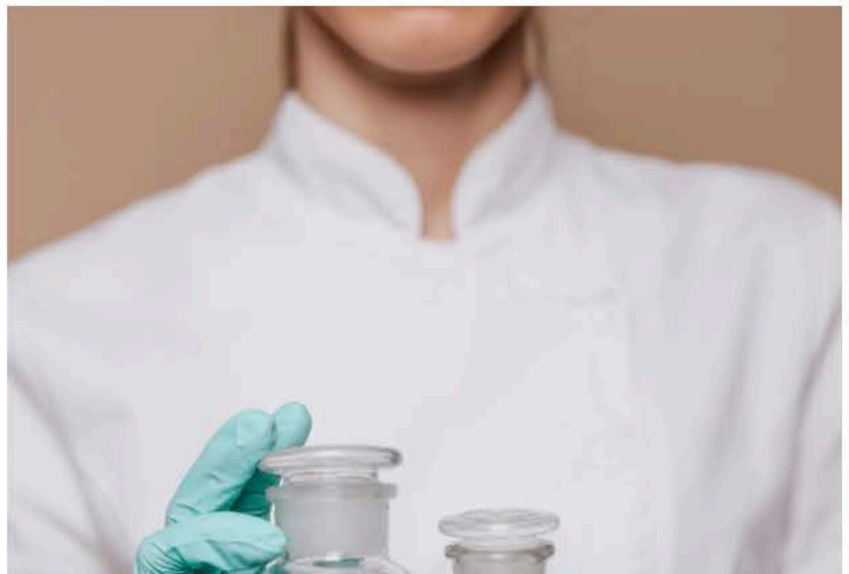

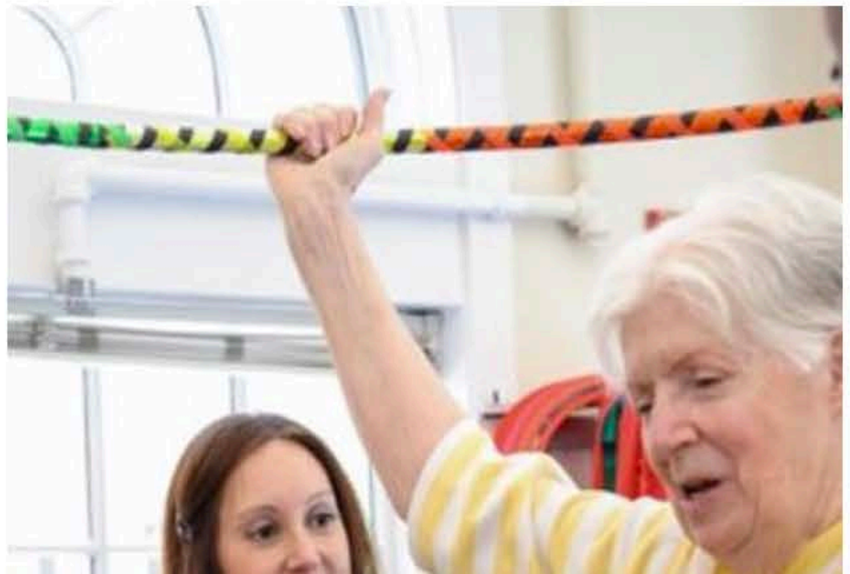

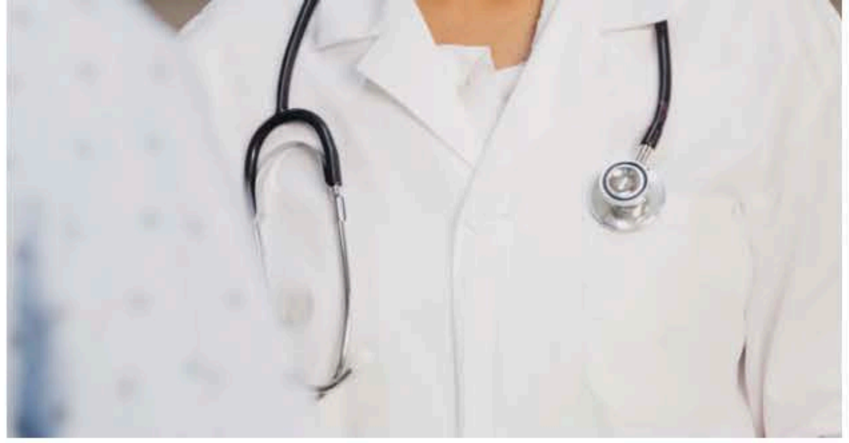

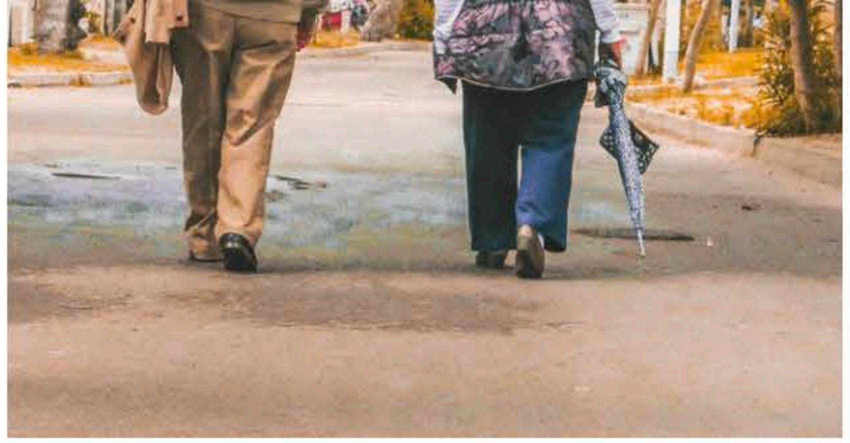

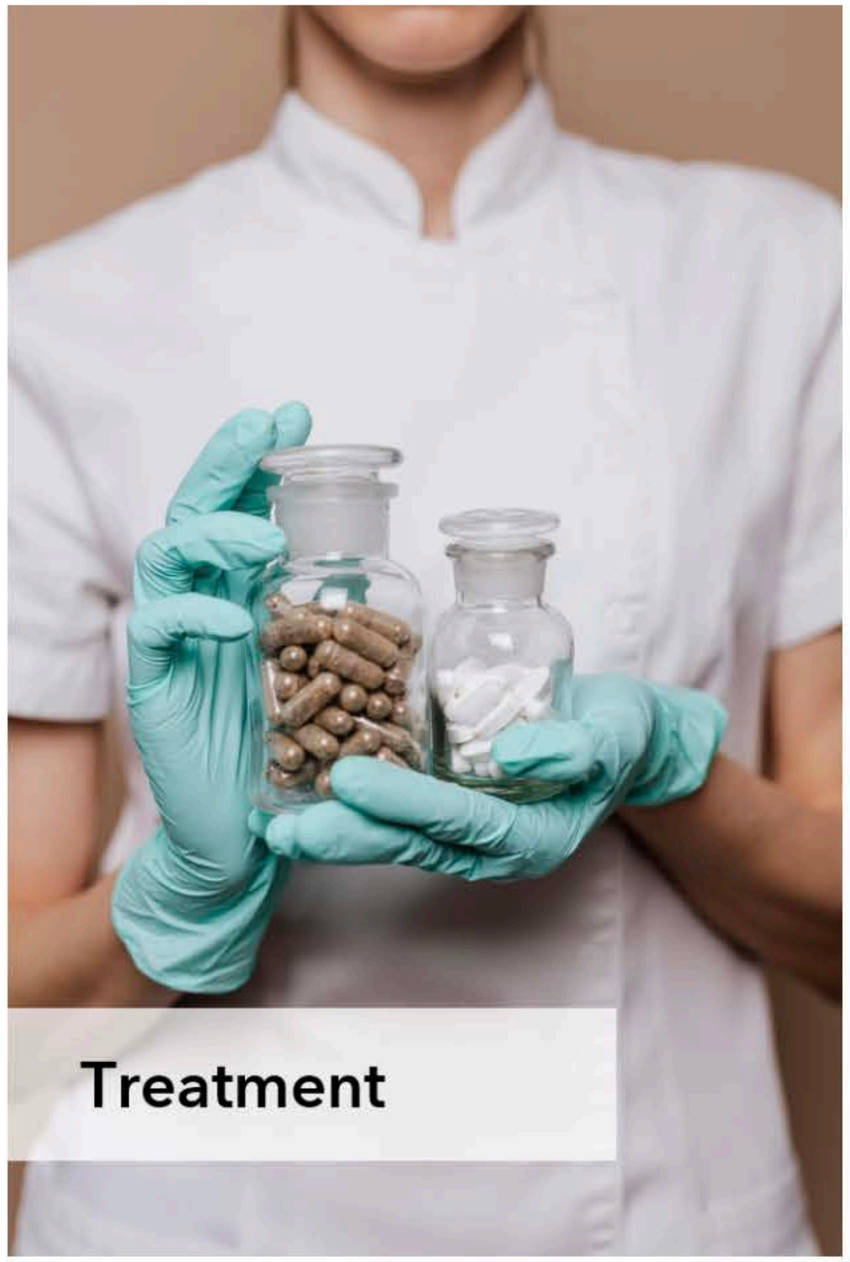

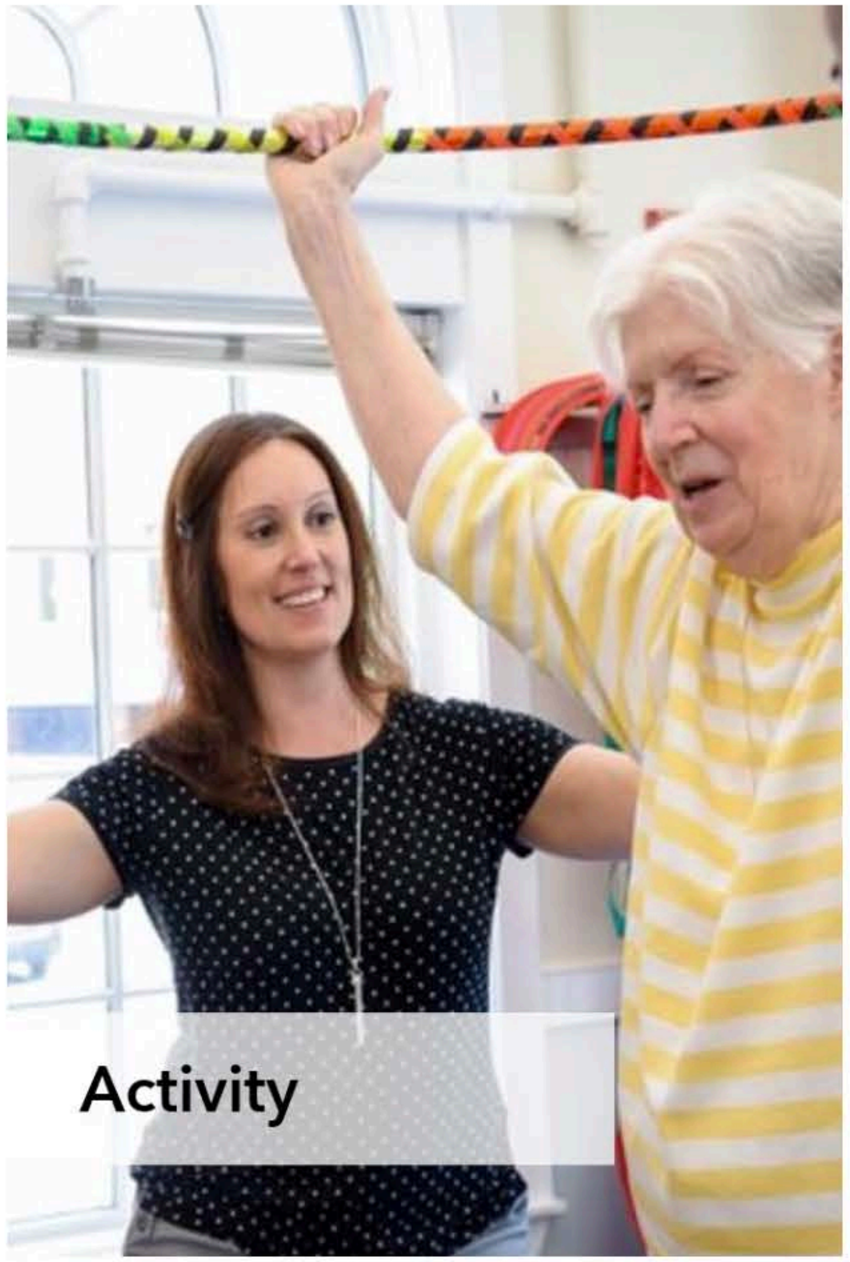

View Glossary Page

Menu

This slide is considered essential information for genetic counseling.

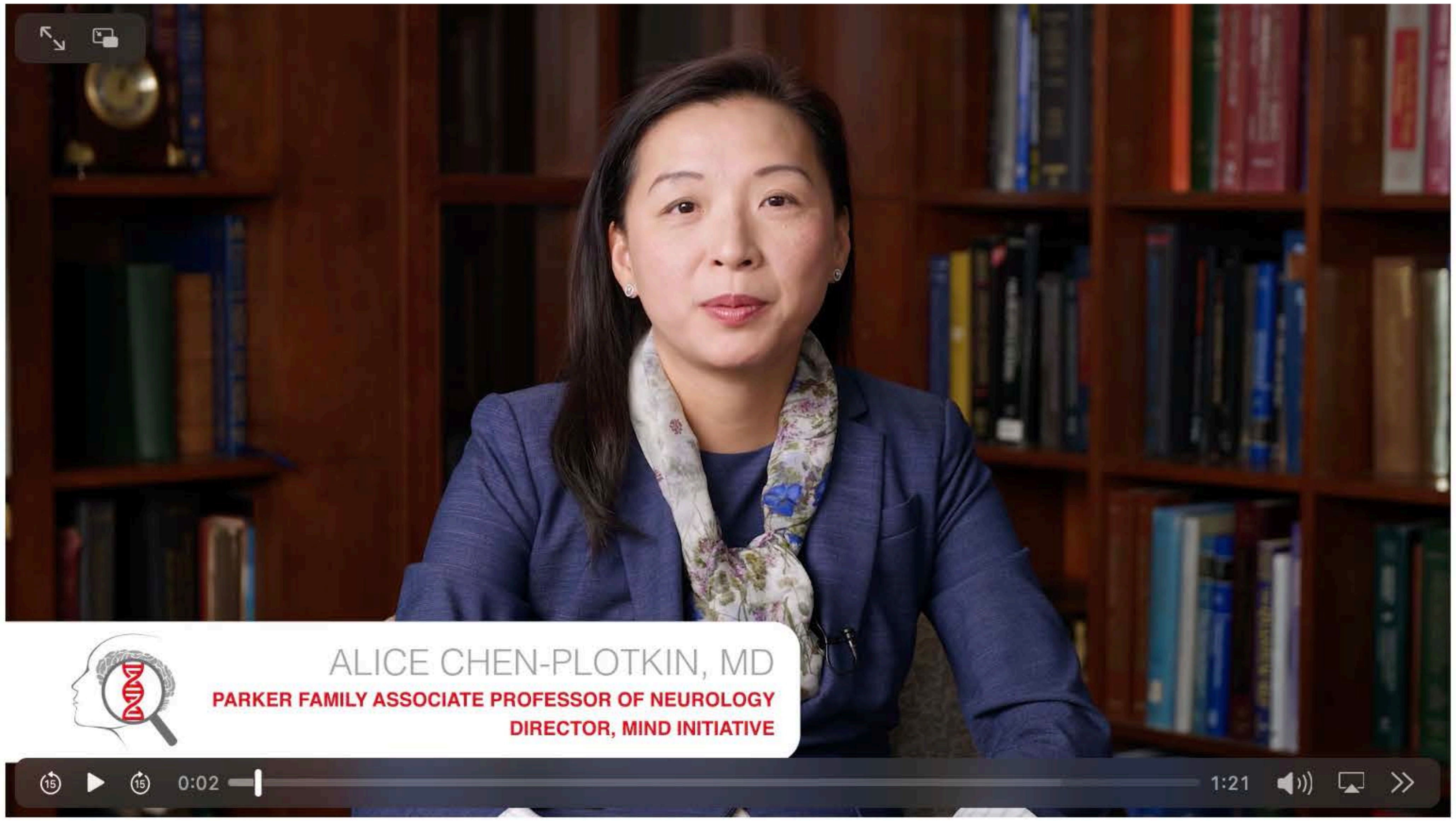

If you would like to read the information provided click *HERE*.

This slide is considered essential information for genetic counseling.

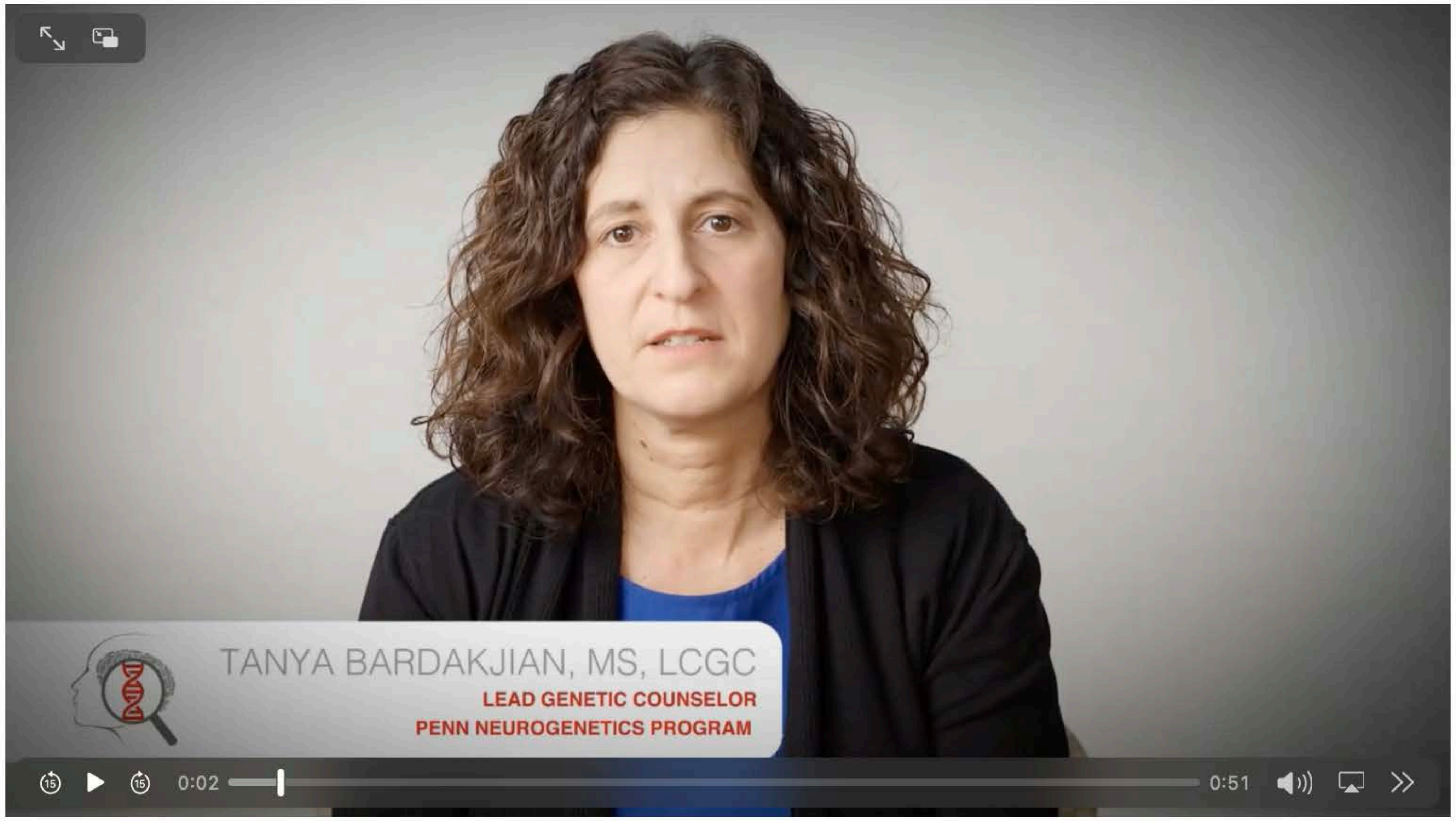

If you would like to read the information provided click *HERE*.

Types Of DNA Changes

Our genes are made up of a series of letters, like the letters in the familiar words shown below.

When one letter is changed below, the meaning of the word does not change. We would still know it is the color gray or grey. Similarly, some changes in the "spelling" of our DNA may not cause disease. These are considered **benign variants**:

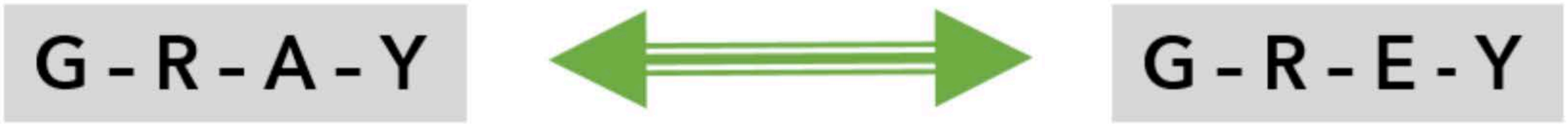

Other times, changing one letter can change the meaning of the word. In genetics, **disease-causing mutations** CAN change how the body functions:

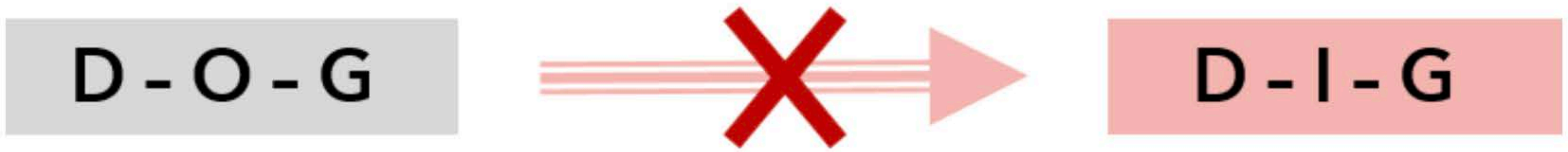

Menu

This slide is considered essential information for genetic counseling.

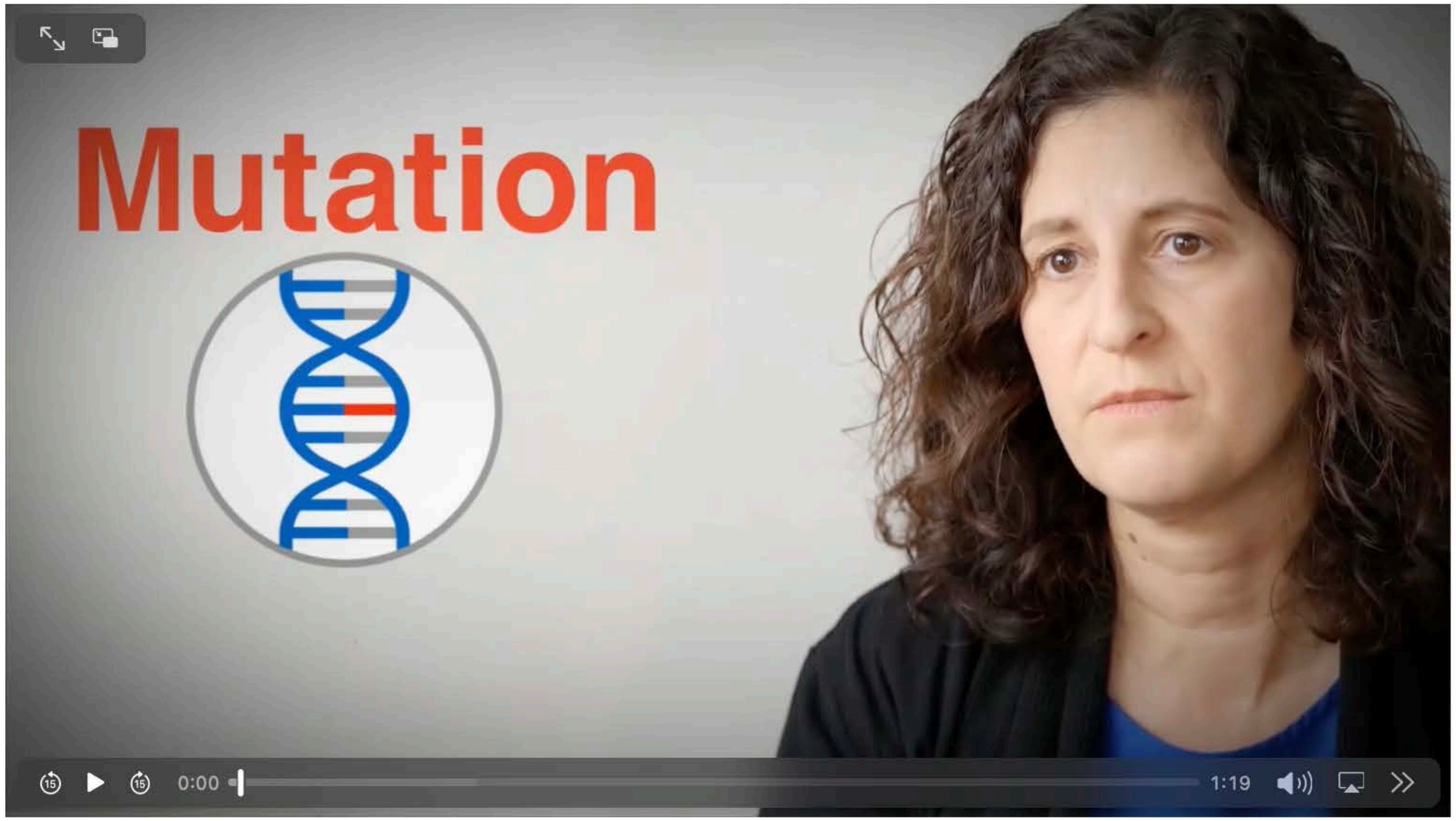

If you would like to read the information provided click *HERE*.

View Glossary Page

What Is Genetic Testing?

Genetic testing is a medical test performed in a laboratory. It requires a sample of blood or saliva.

Genetic testing can find mutations that are linked to Parkinson's disease.

Genetic testing is **not a single test**. There are many different types of genetic testing.

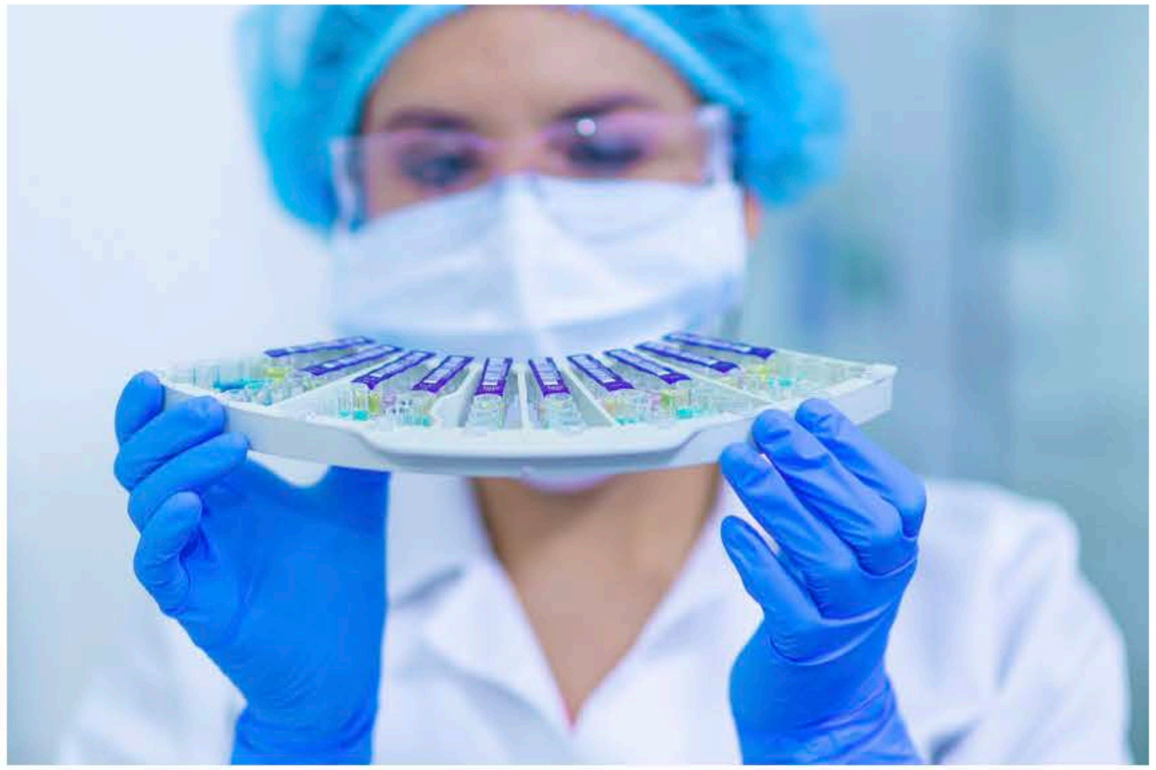

Menu

Listen to page content.

Hover over the pictures below to learn more about the specific types of genetic tests that can be done.

Targeted Genetic Testing

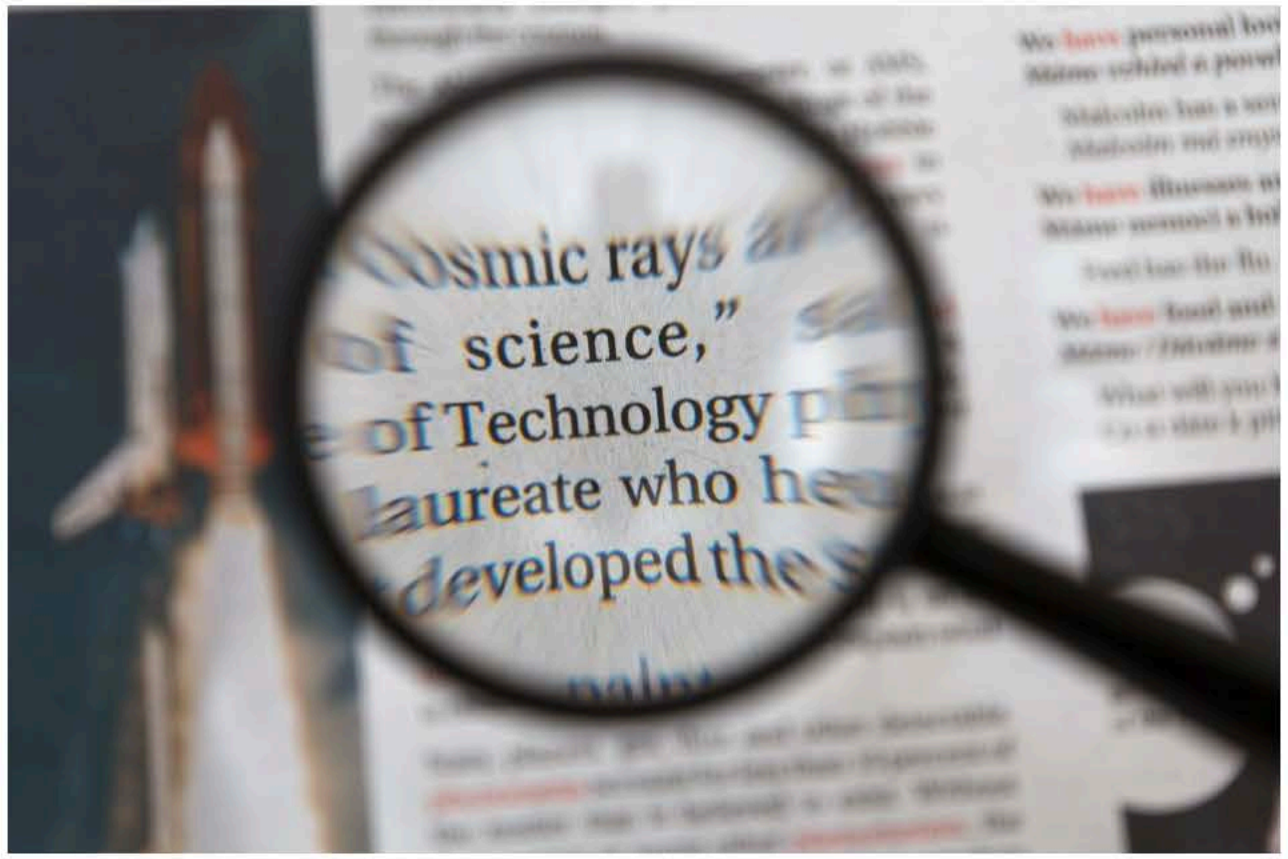

Full Gene Testing

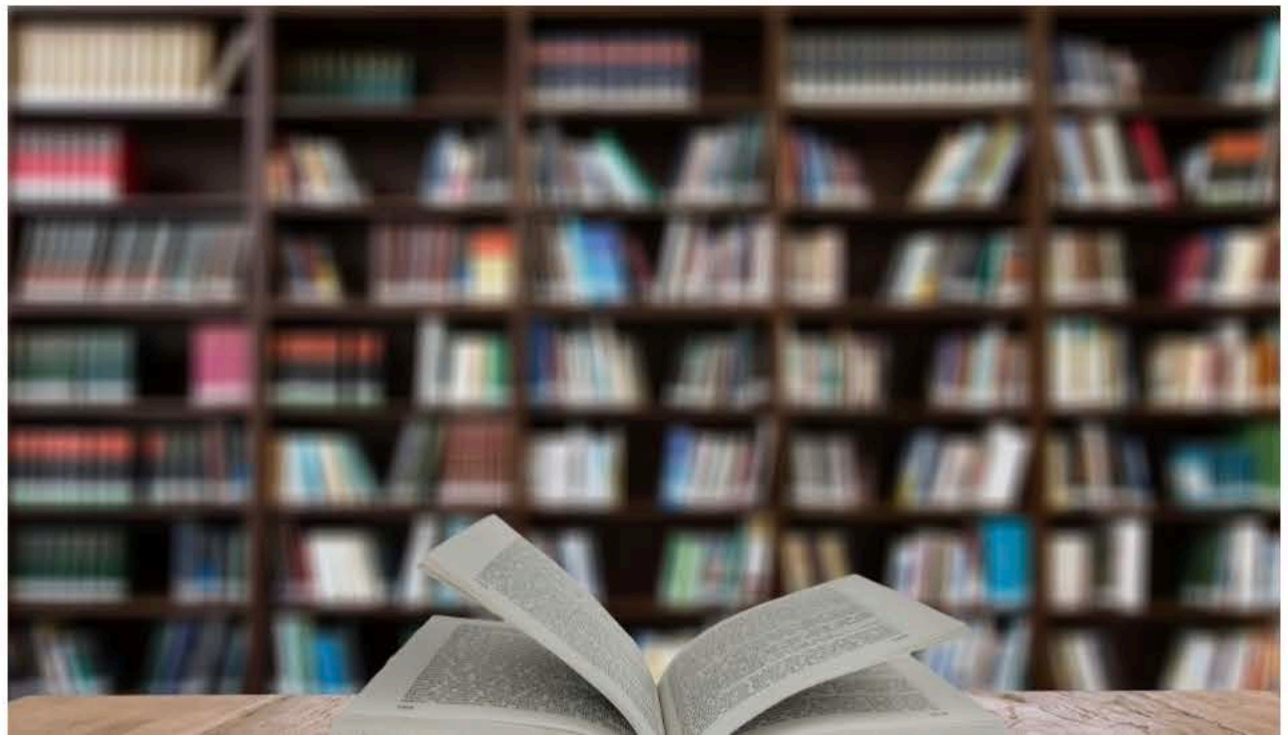

Full Gene Testing

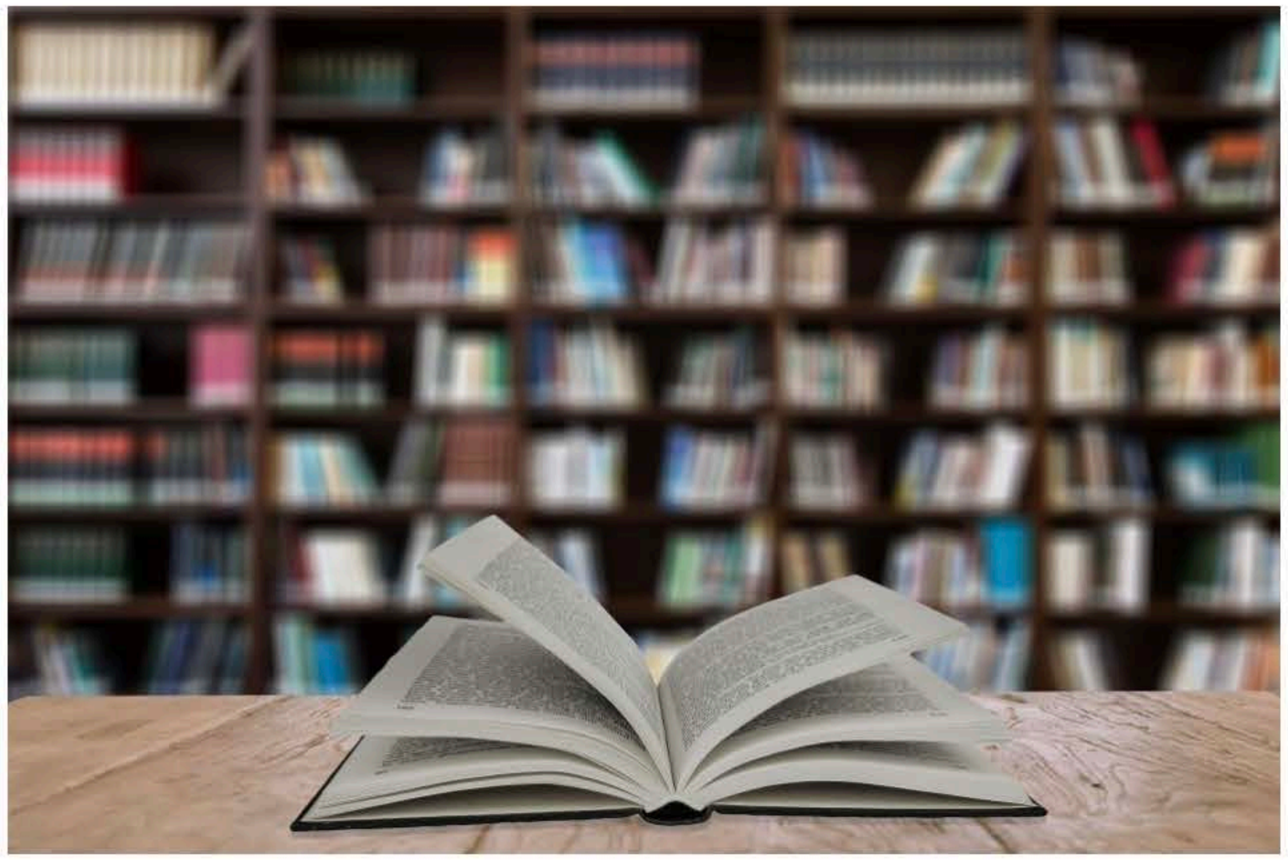

Multi-Gene Testing

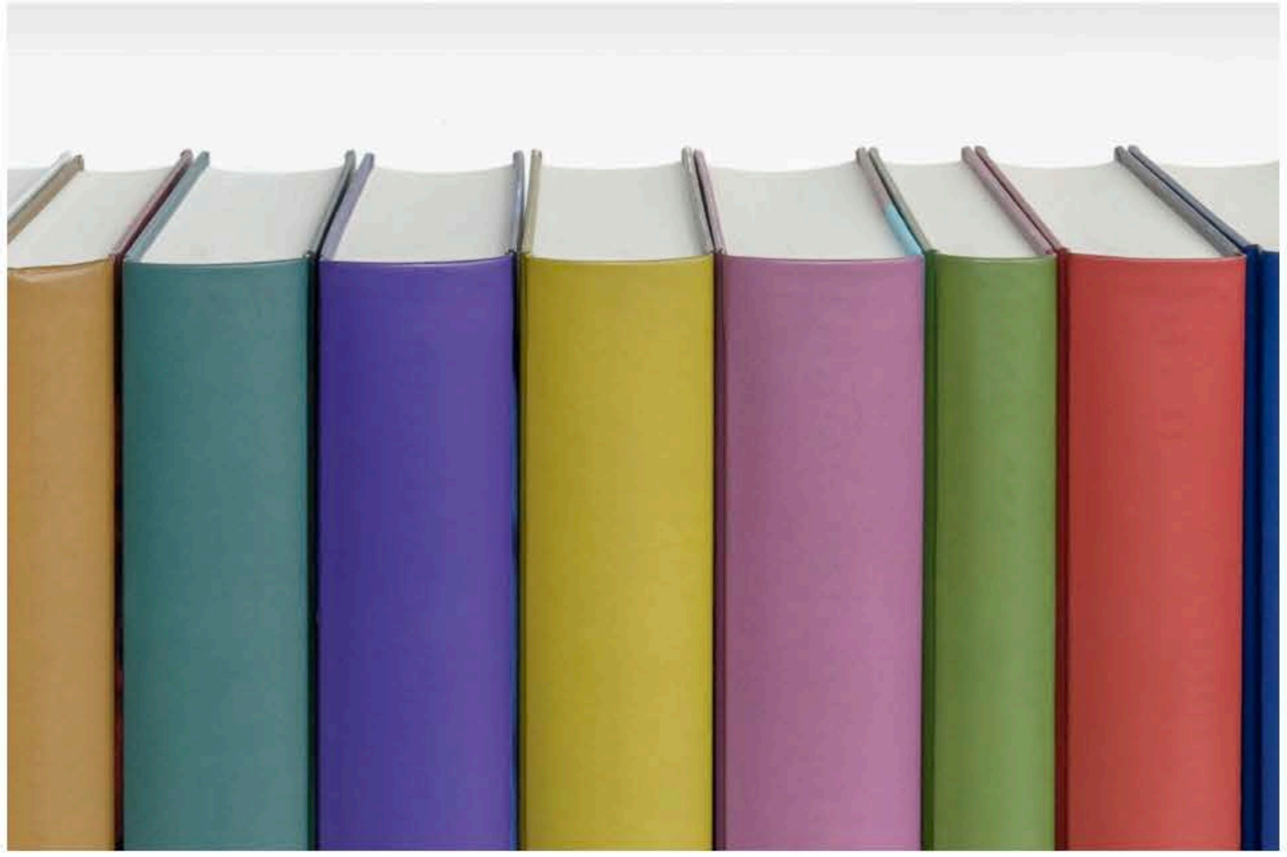

Click *HERE* if you would like to learn more about genetic testing.

Back

10

Contact Us

Next

Reasons To Consider Genetic Testing

There are many reasons to consider genetic testing, including:

- You want to know if you have a gene mutation that caused Parkinson's disease.
- You want to know if you are eligible for research studies.
- You want to know if you are at increased risk of developing Parkinson's disease in the future.
- You are in a research study related to learning more about the genetics of Parkinson's disease.

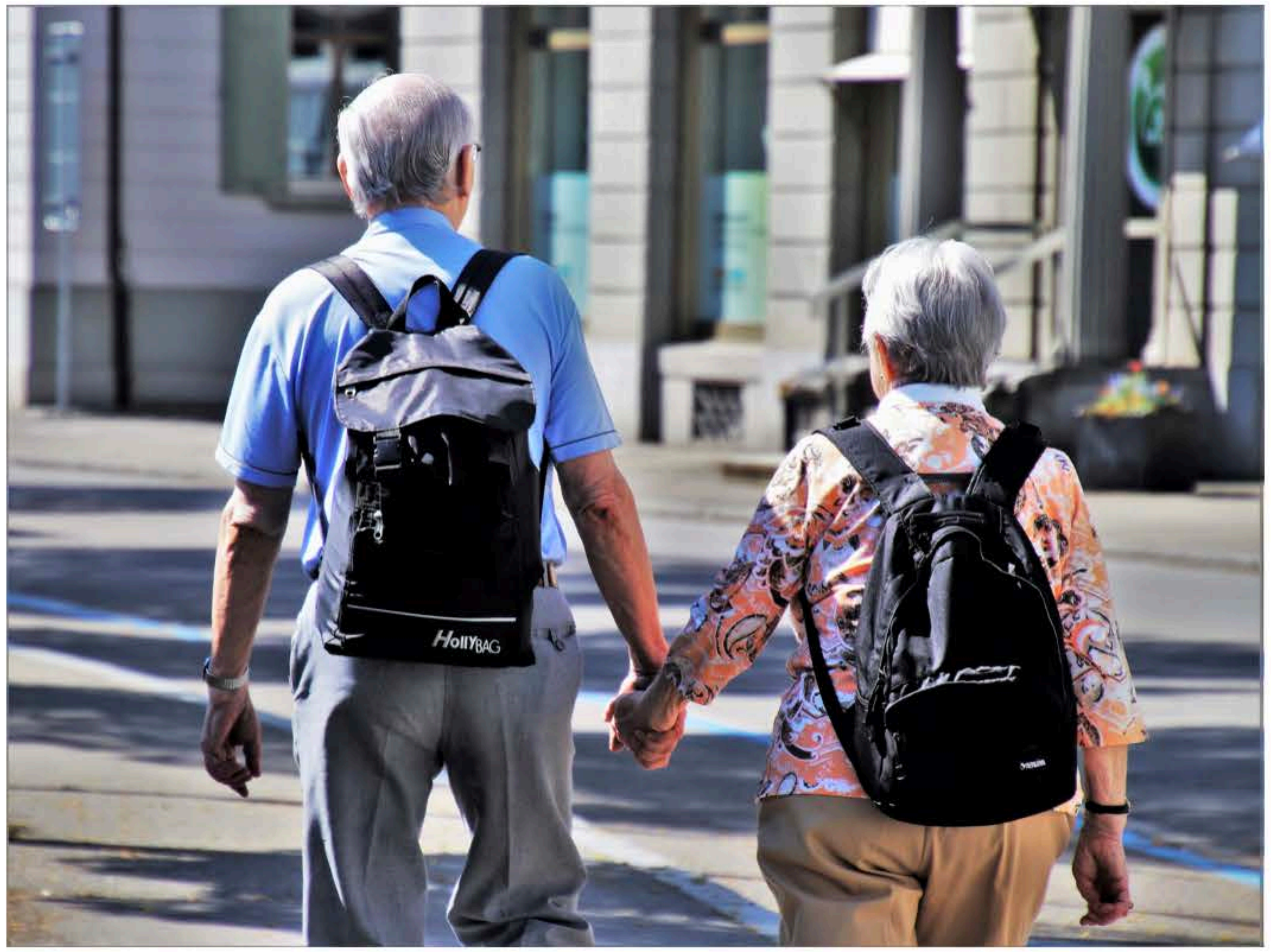

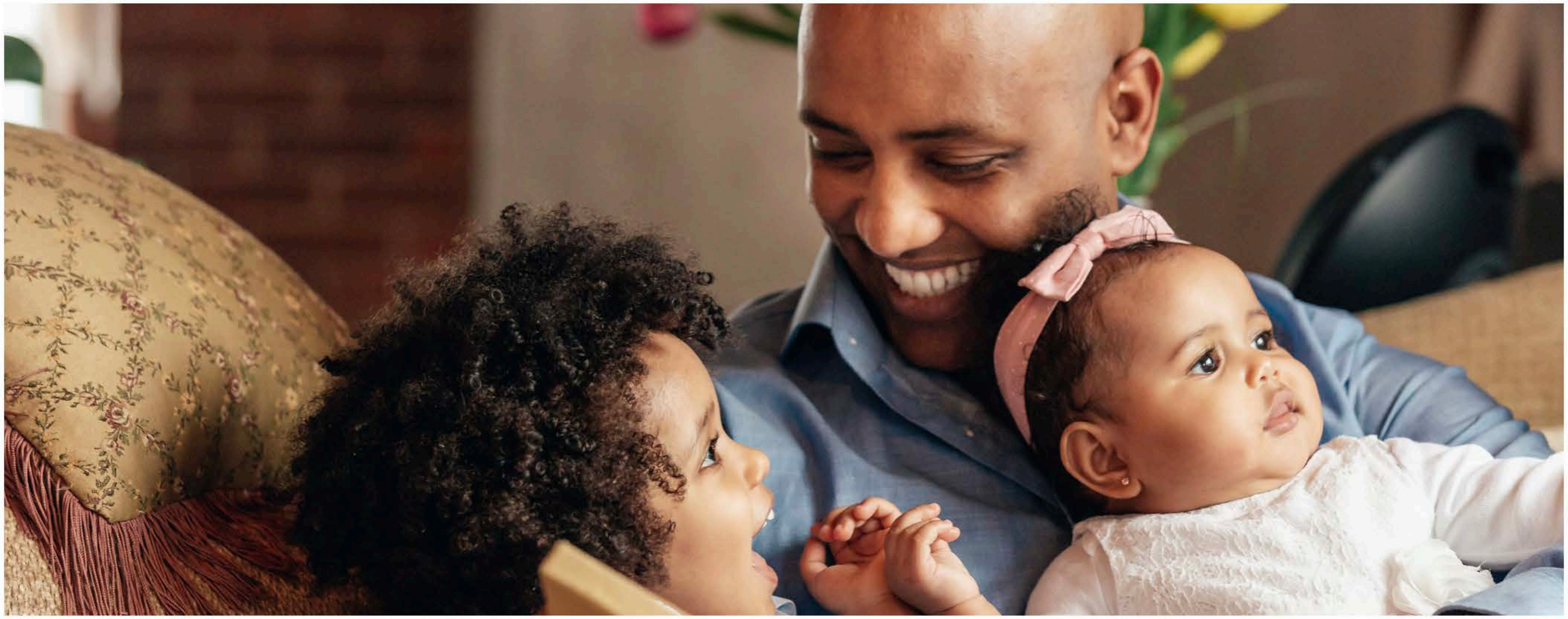

This study is only looking at specific genetic mutations in Parkinson's disease.

Other reasons people may have genetic testing could include:

- Specific testing to see if you will develop a disease in the future.
- Genetic testing for family planning.
- Newborn screening.
- Other research testing related to the genetics of Parkinson's disease.
- Clinical testing for other diseases in the family.
- **These types of testing will NOT be part of this current research study.**

If you have questions, you may discuss with the genetic counselor. You may also select "contact us" below to contact the study team.

It is important that you consider these risks and benefits when deciding if genetic testing is appropriate for you.

Potential Benefits

1. Testing may be able to provide an answer about why you developed Parkinson's disease.
2. You may benefit emotionally from learning the results. Some people may feel relief from uncertainty.
3. Results may be used to help you and your doctor make informed decisions about your health.
4. Some results may allow you to participate in other research studies. Some research is focused on specific genetic mutations.
5. Genetic results may provide more information for your family. Genetic counseling is available for you as well as your family.
6. Some genetic results can be used in family planning.

Potential Risks

1. You may have negative emotions when learning the results. Some people can feel angry, sad, or anxious. Some may experience guilt from their results.
2. Your genetic results may affect your family members. Genetic counseling is available for you and your family members.
3. Even after genetic testing, there may be some uncertainty for you and your family.
4. You may be concerned about discrimination. Click *HERE* to learn more about federal laws in place to protect you.
5. For those who do not have Parkinson's disease, finding out that you carry a genetic mutation that increases your risk of developing Parkinson's disease is information that cannot be "unlearned" once you have learned it.

Hover over the pictures below to read about the process of genetic testing. If you are using a mobile device or tablet, you may touch the image to reveal the text.

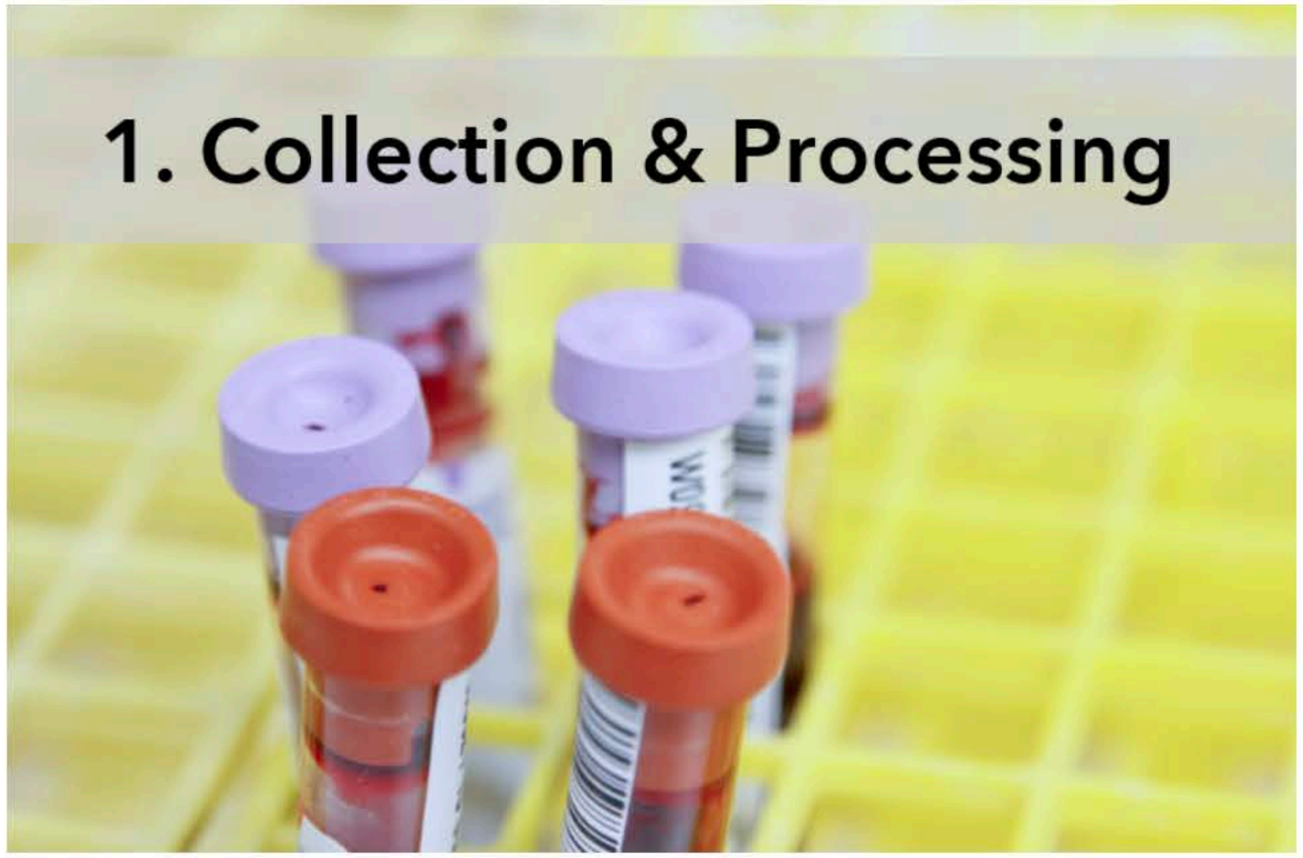

The laboratory sequences and studies the DNA. They will only interpret and report what was requested at the time of the testing. If you have more genetic testing in the future, it could require another sample.

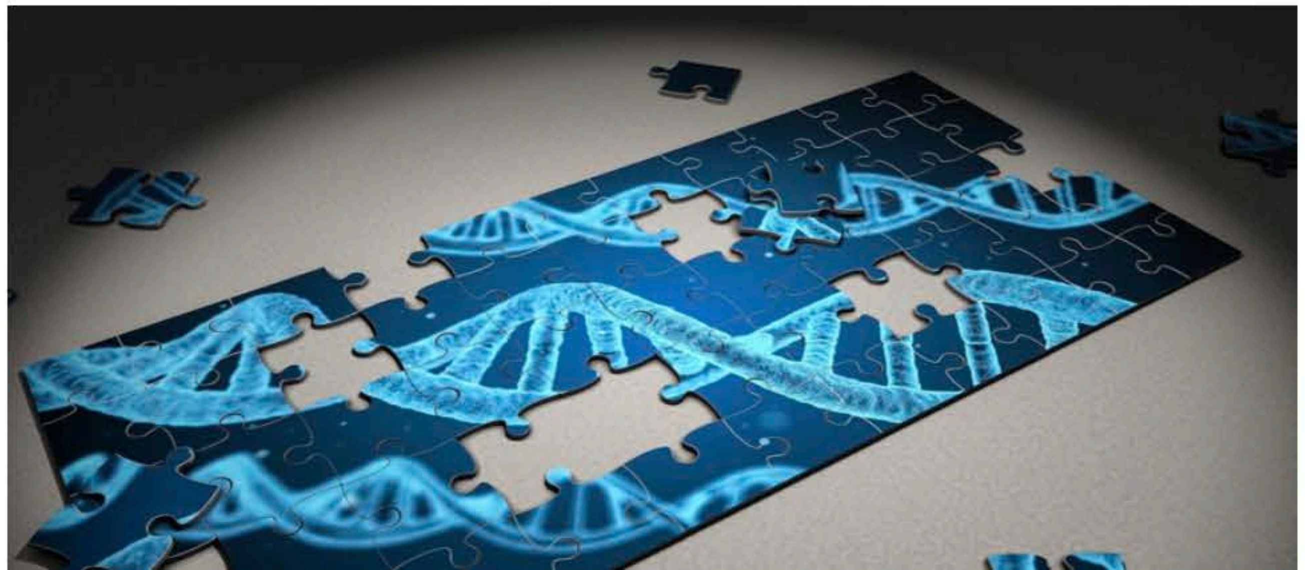

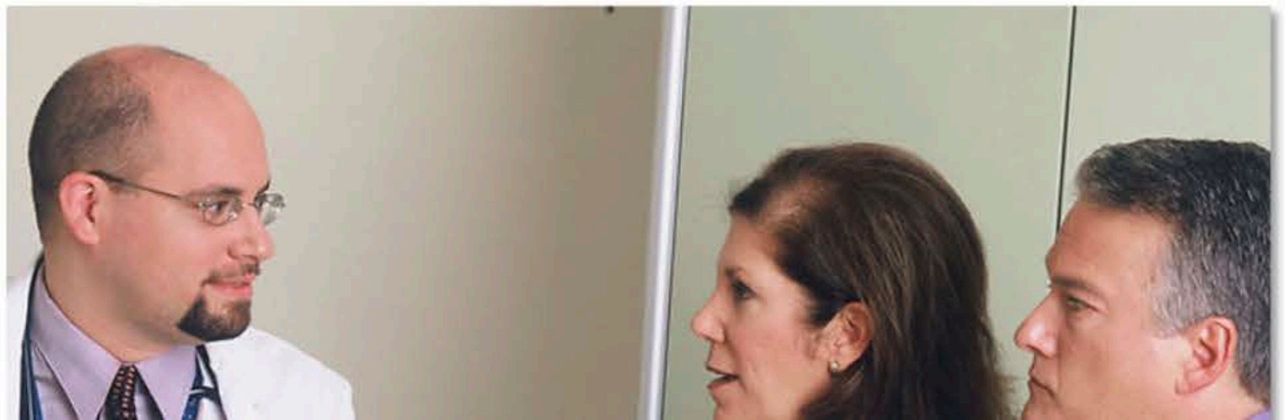

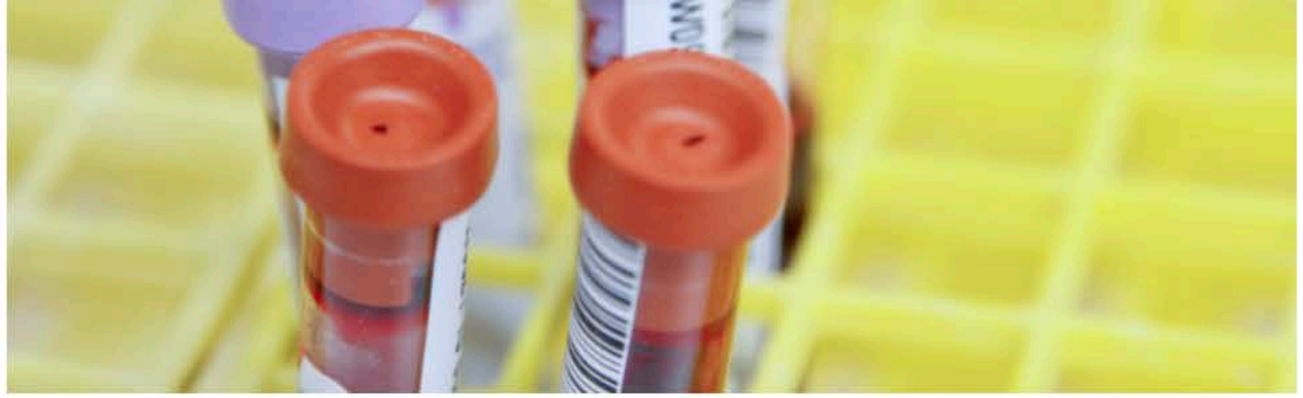

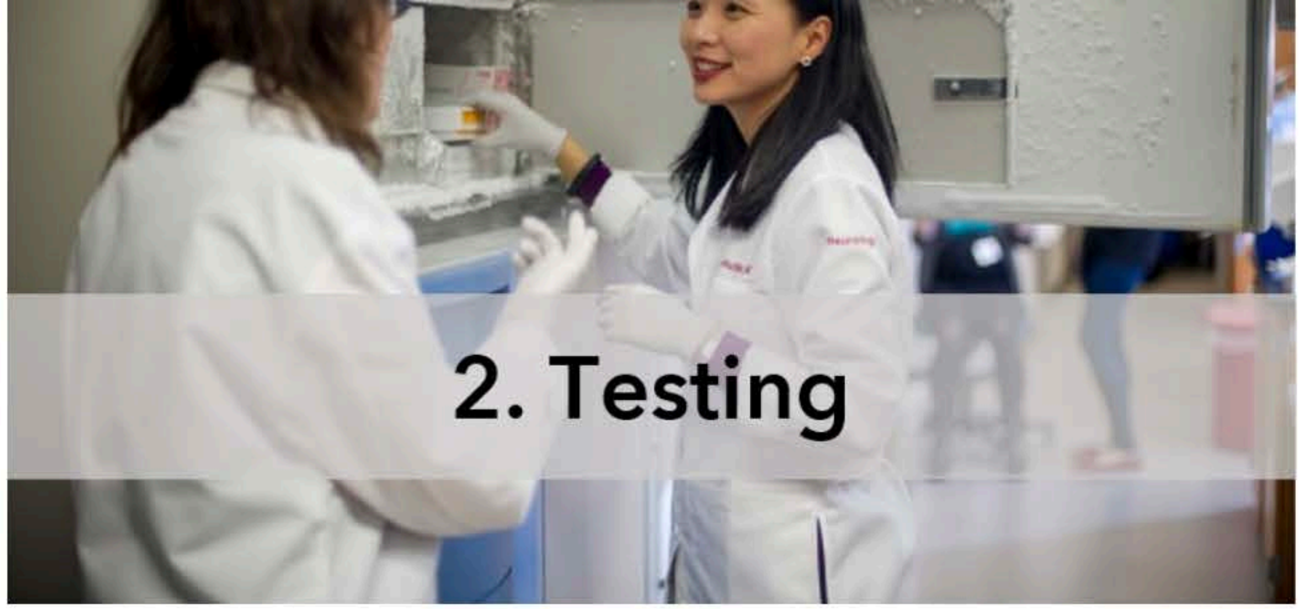
2. Testing

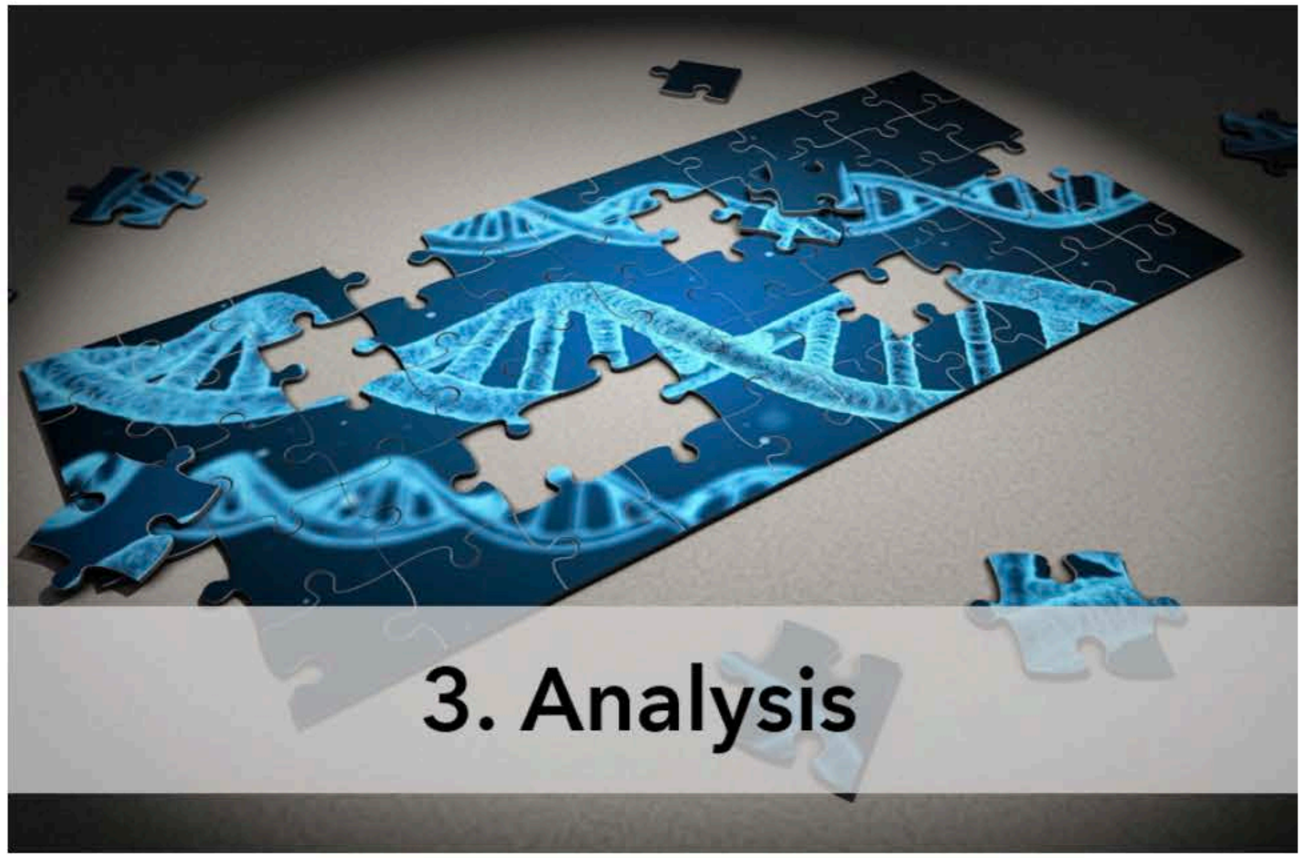
3. Analysis

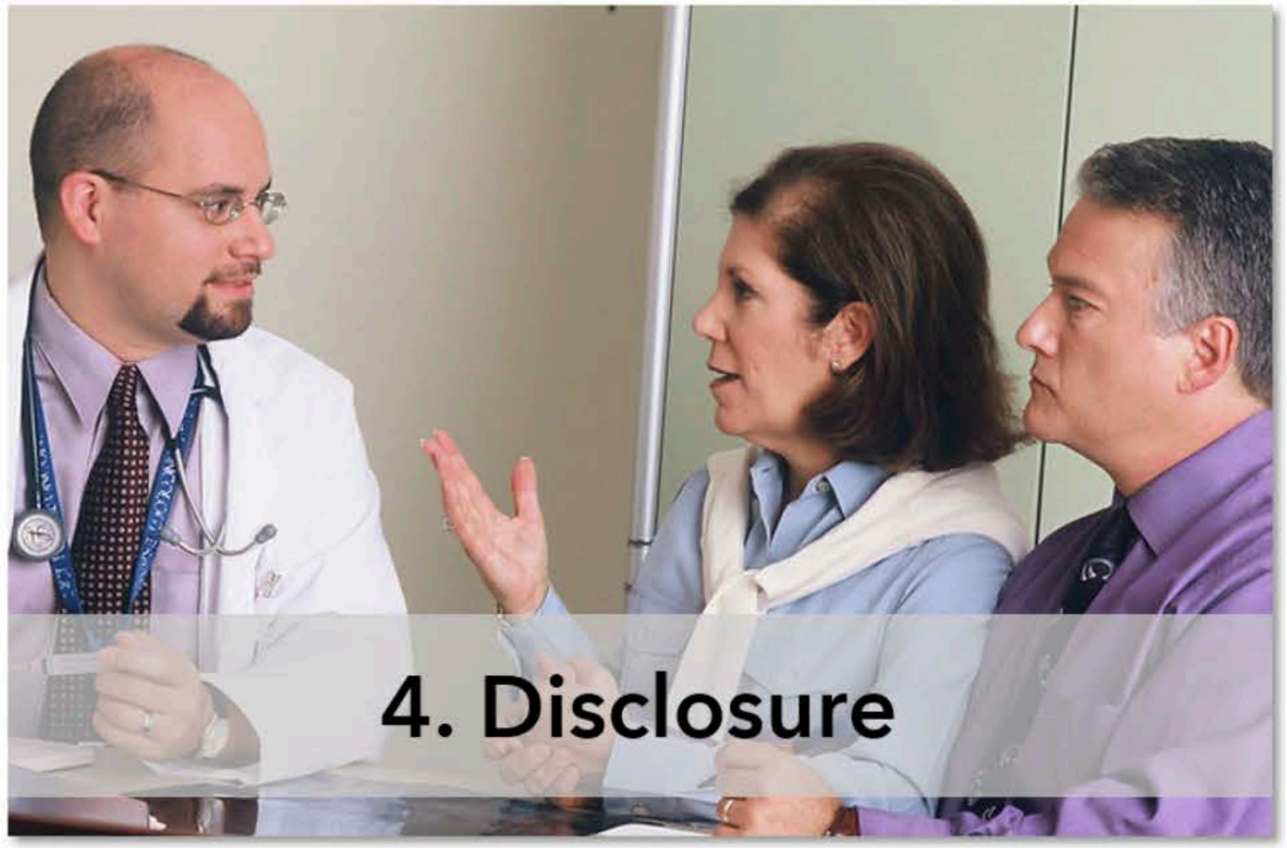
4. Disclosure

Getting Genetic Testing Results

This slide is considered essential information for genetic counseling.

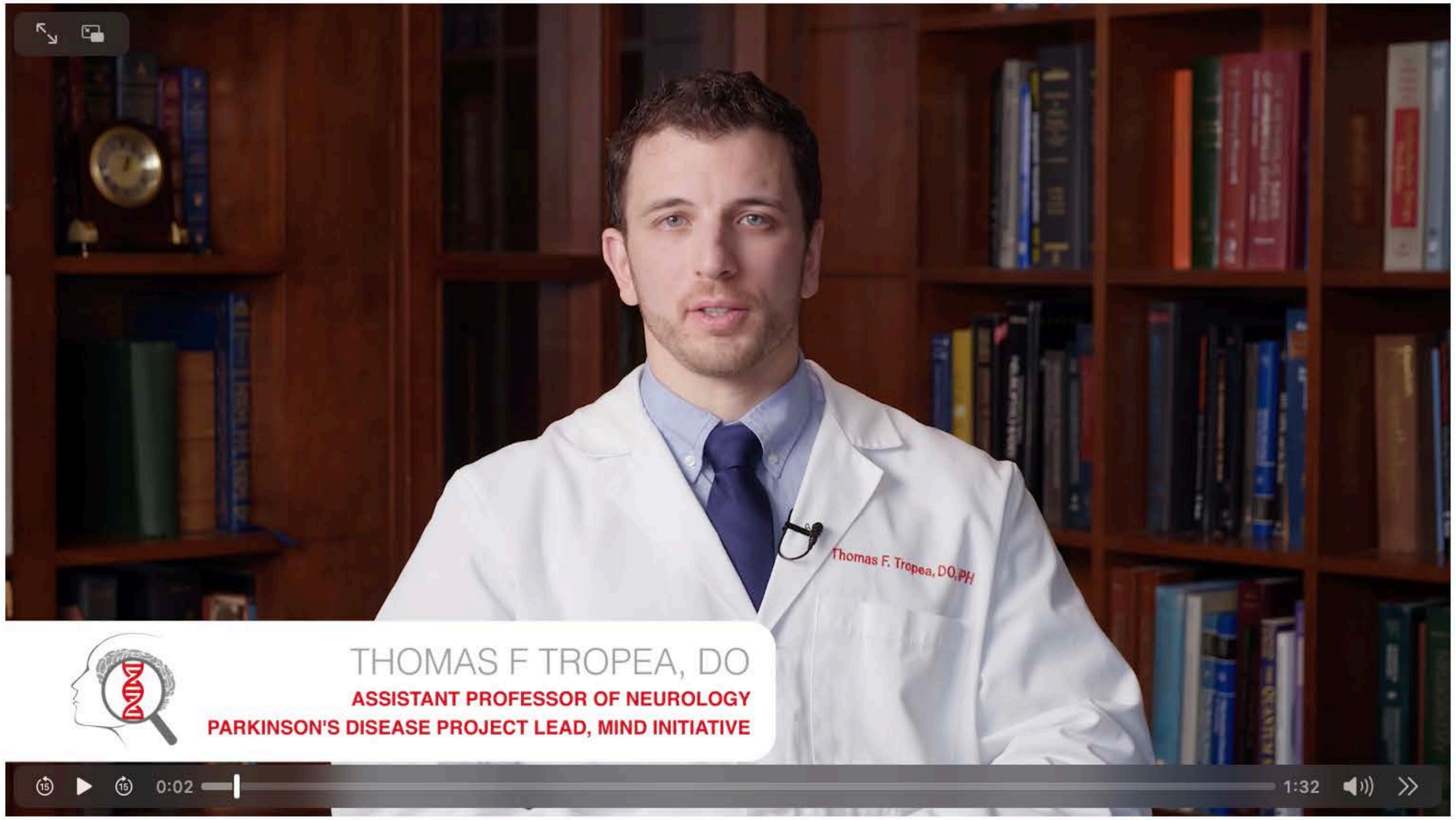

If you would like to read the information provided click *HERE*.

The *GBA* gene is the most common genetic risk factor for Parkinson's disease.

- Overall, about 5% of people with Parkinson's disease have a *GBA* mutation.
- In people of Ashkenazi Jewish descent, this is more common. About 10-15% of Ashkenazi Jewish patients with Parkinson's disease have a *GBA* mutation.
- Having one (1) mutation in *GBA* increases the risk of Parkinson's disease. This is compared to those without a *GBA* mutation.

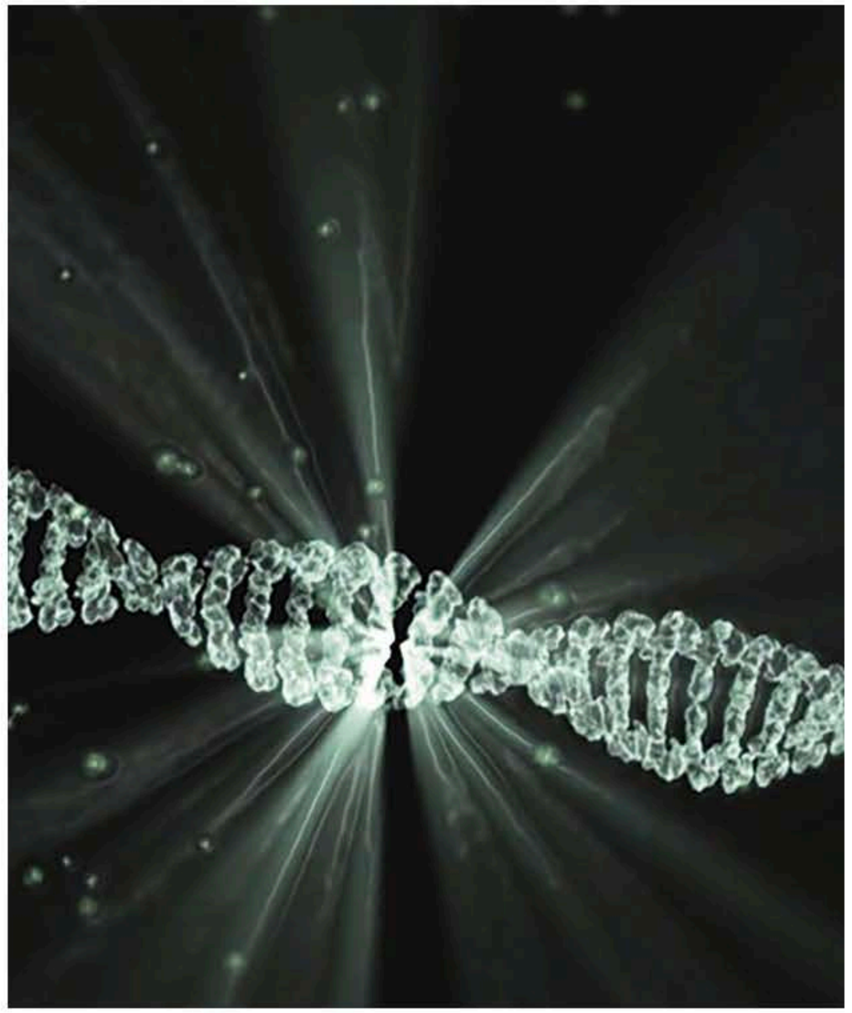

The *GBA* gene gives the instructions to make a protein called beta-glucocerebrosidase.

- The normal function of this protein is to process waste in your cells. A mutation in the *GBA* gene can affect the function of this protein.
- Researchers are still learning about how *GBA* mutations may affect the risk for Parkinson's disease.
- *GBA* mutations show reduced penetrance for Parkinson's disease. This means that some people with a *GBA* mutation will NOT have Parkinson's disease.
- Genetic counseling is available for people with a *GBA* mutation and their family members. This will be included during this study for those who have genetic testing done.

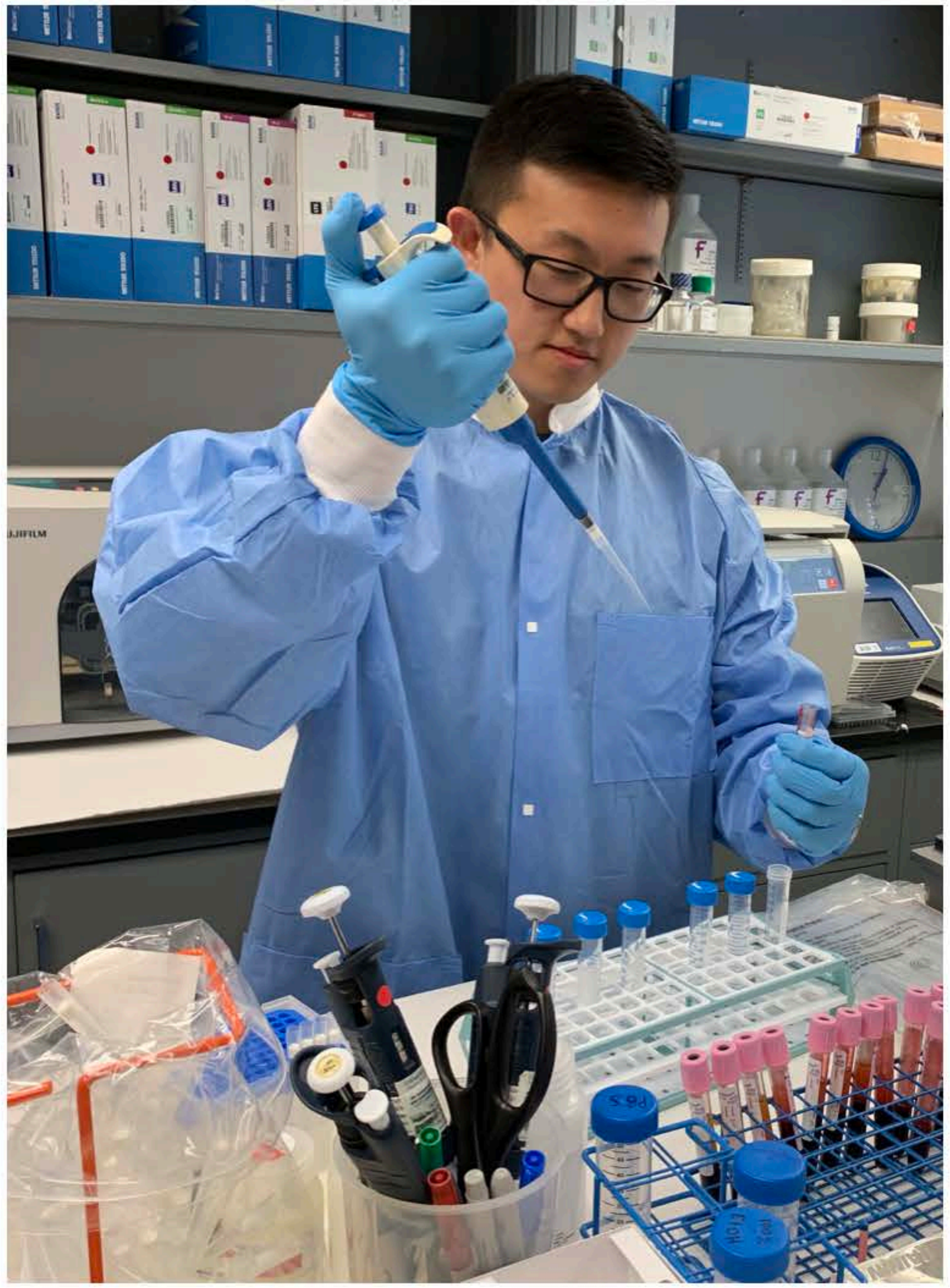

Mutations in *GBA* are related to another health condition called Gaucher's disease.

- Gaucher's disease is a disorder that affects many different body parts. It causes low blood counts, fatigue, and easy bruising among other things.
- People with Gaucher's disease may also develop Parkinson's Disease.
- Click *HERE* if you would like to learn more about Gaucher's disease.
- People with Gaucher disease have two (2) mutations in the *GBA* gene. One was inherited from their mother and the other from their father.
- Thus, individuals with a *GBA* mutation may also be at risk to have a child or grandchild with Gaucher disease.

If you have questions about Gaucher disease, you may discuss this with the genetic counselor after your results. If you have questions now, you may select "contact us" below to contact the study team.

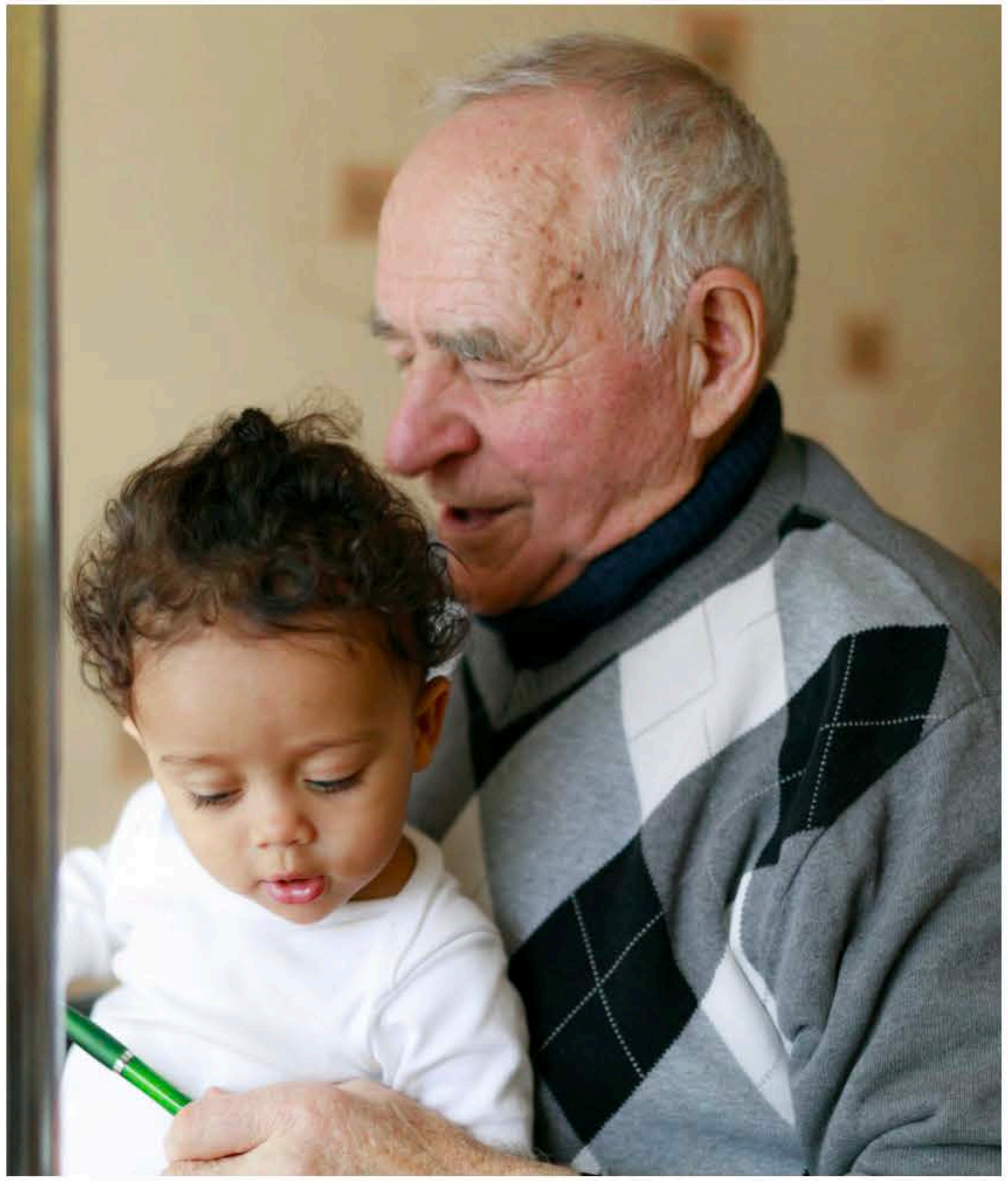

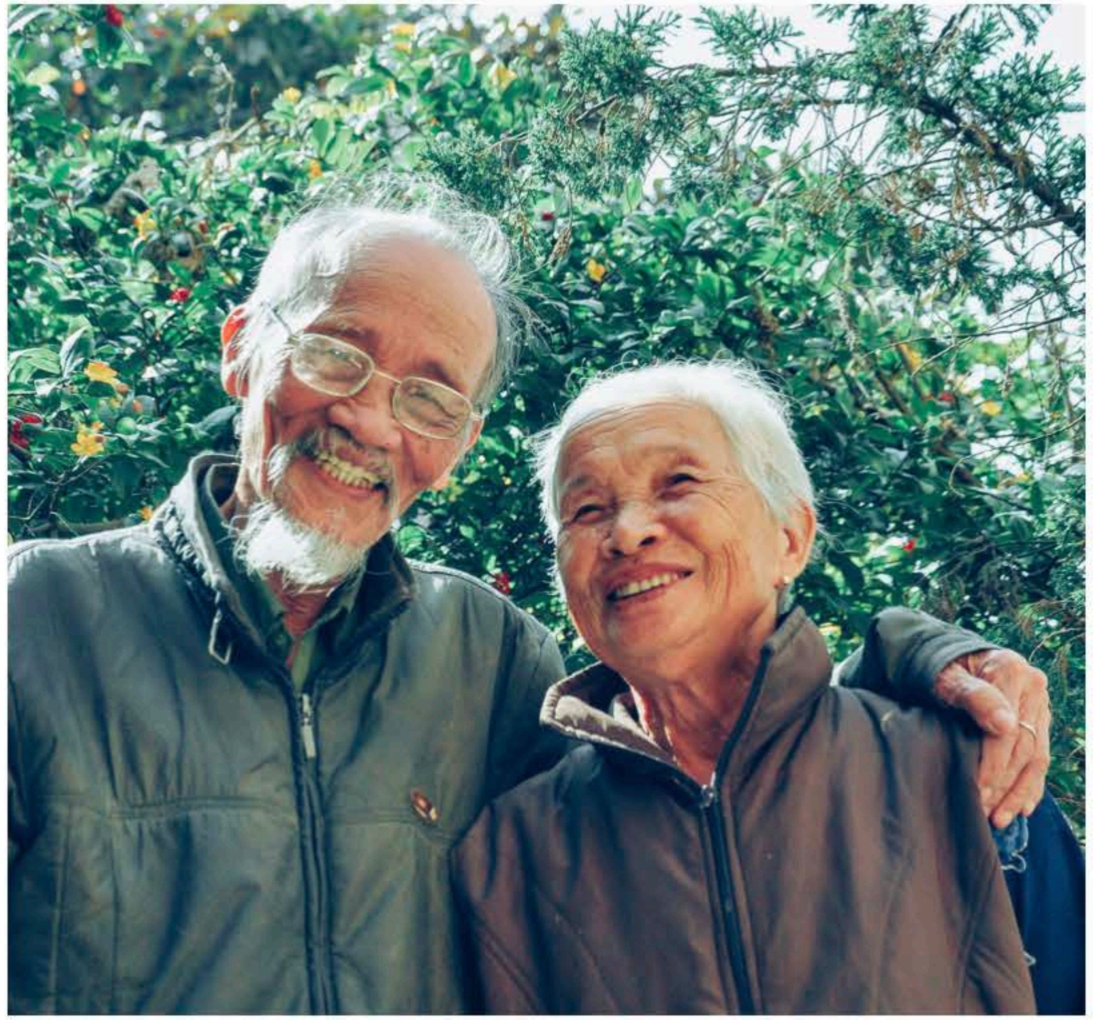

The *LRRK2* gene is also a common genetic factor in Parkinson's disease.

- 1-2% of all people with Parkinson's disease have a *LRRK2* mutation.
- This is more common in people of Ashkenazi Jewish or North African descent.
- Having one (1) mutation in *LRRK2* increases the risk of Parkinson's disease. This is compared to those without a *LRRK2* mutation.

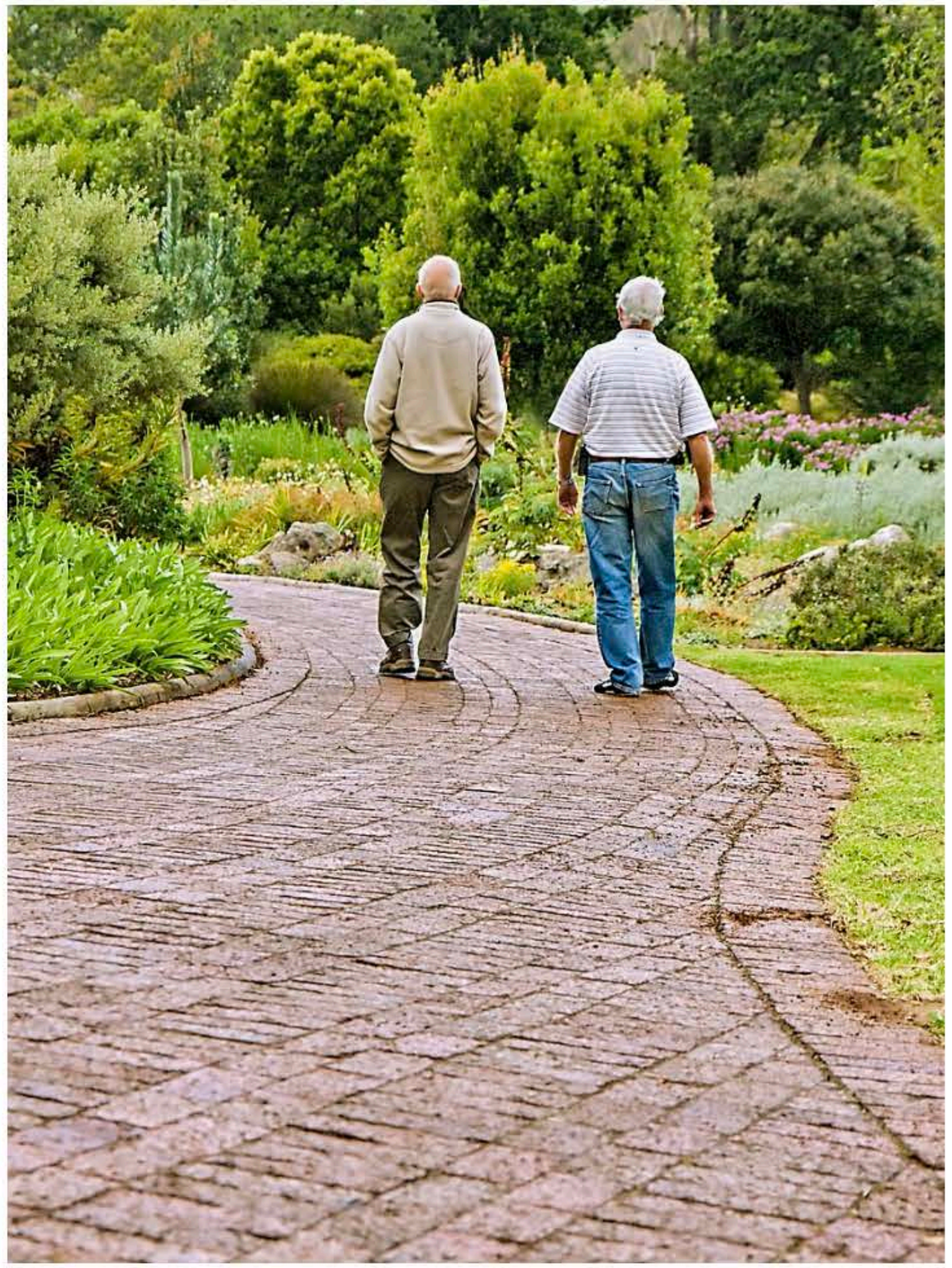

LRRK2 gives instructions to make a protein called leucine-rich repeat kinase 2. This protein is also called dardarin.

- The *LRRK2* protein has many different functions in the cells of your body.
- A mutation in the *LRRK2* gene can affect the function of this protein.
- Researchers are still learning about how *LRRK2* mutations increase the risk for Parkinson's disease.

Genetic counseling is available for people with a *LRRK2* mutation and their family members. This will be included as part of this study for those who have genetic testing done.

LRRK2 may also be associated with an increased risk of Chrohn's disease. A referral to a specialized physician may be recommended.

- Having 1 or 2 mutations in *LRRK2* carries the same risk of Parkinson's disease.
- Unlike *GBA, LRRK2* is NOT known to be related to a recessive disease.

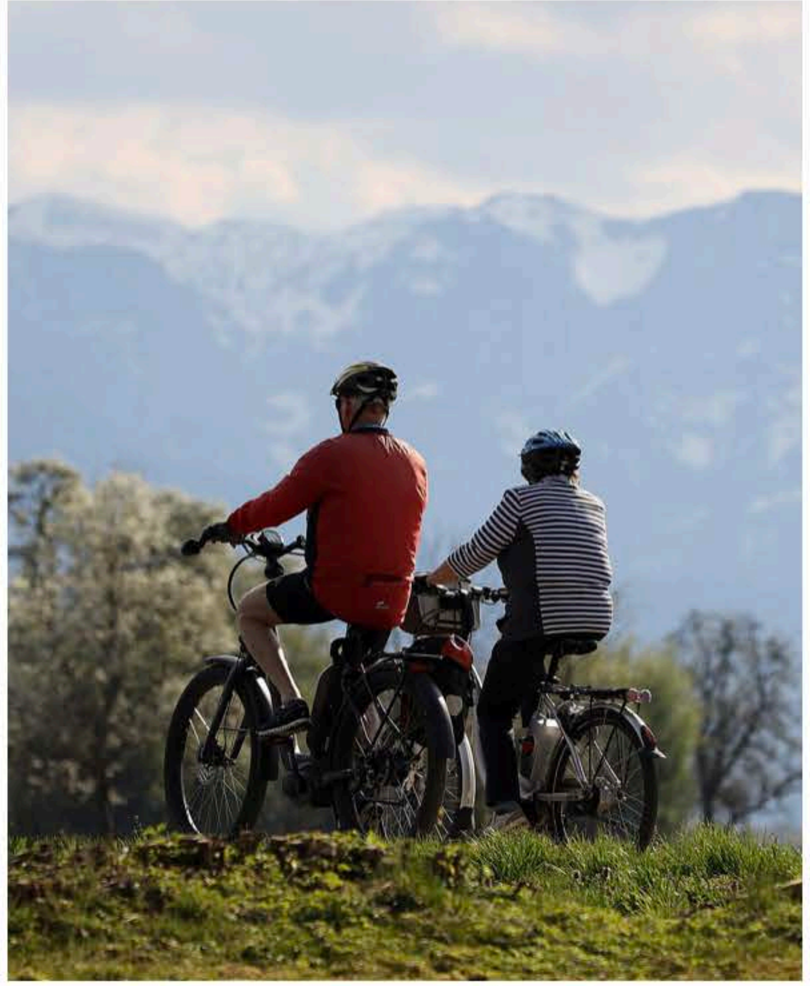

Limitations of Genetic Testing

This slide is considered essential information for genetic counseling.

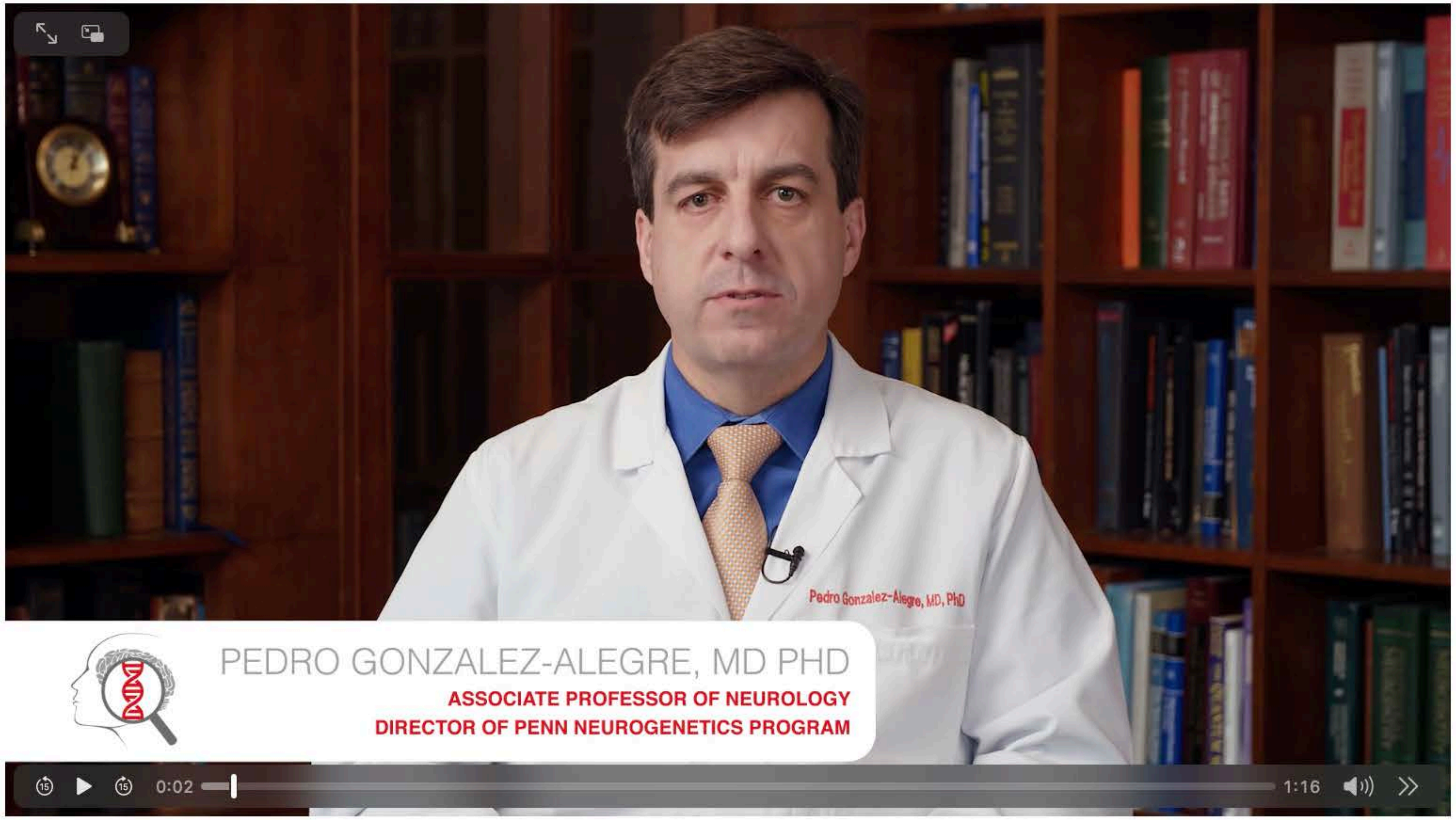

If you would like to read the information provided click *HERE*.

Other Genetic Causes of Parkinson's Disease

In this study, we will only look at twenty-two (22) specific mutations. They are in two genes, *GBA* and *LRRK2*. These mutations are the more common ones in Parkinson's disease. However, they are not the only genetic mutations in Parkinson's disease.

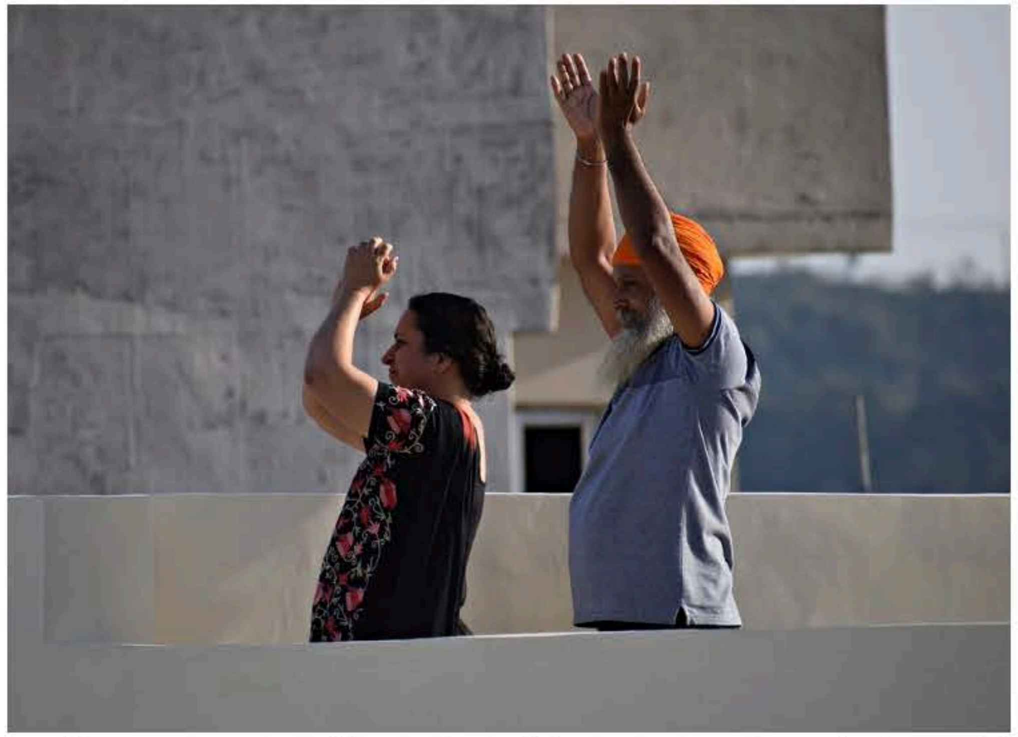

There are other genetic causes of Parkinson's disease. They are beyond this current study. Click here to learn more about the genetics of Parkinson's disease.

Clinical genetic counseling is available for you to learn more. You may discuss a referral with your physician. You can also contact Penn Neurology by calling (215) 829-6500.

This concludes your online pre-test genetic counseling. If you are interested, you may now have genetic testing done as part of this research study.

In this study, the genetic testing will ONLY include twenty-two (22) common mutations in *GBA* or *LRRK2*. You will learn your results during a telephone or video call with the genetic counselor.

During genetic counseling, you will receive either:

- A **positive** result, meaning that we found a genetic mutation related to Parkinson's disease. You will be able to discuss what this result means for you and your family during the results disclosure visit. This result would NOT mean that other people in your family have Parkinson's disease. However, they may have the same mutation. We can refer your family members for genetic counseling to learn more.

- A **negative** result, meaning that none of these 22 mutations were found. You will continue to be treated by your physicians the same as before the genetic testing. This does NOT mean that you do not have Parkinson's disease. It also does not mean that your Parkinson's disease is not genetic. Rather, it means that we did not find the cause within these 22 mutations. You may be able to have more genetic testing in the future. This may be with your physician, a genetic counselor, or other genetics research studies. Referral to clinical (non-research) genetic counseling is also available.

Conclusion

Please click any of the topics below to view them again. You can also use the menu on the left.

You can also contact the study team by clicking the "contact us" button on the bottom of the page.

An introduction to Parkinson’s disease

The role of genetics in Parkinson's disease

Important concepts in genetics

Limitations of genetic testing

Specific genes associated with Parkinson's disease
